# Supplementary material for: Inequalities in cardiovascular risks among Swedish adolescents (ABIS): a prospective cohort study
Source: BMJ Open. 2020 Feb 20;10(2):e030613. doi: 10.1136/bmjopen-2019-030613 (PMC7044991; doi:10.1136/bmjopen-2019-030613)
Supplement: Supplementary data [file bmjopen-2019-030613supp001.pdf]

Supplementary table 1: Comparison of original ABIS cohort, invited subsample, participants and non-participants in the cardiovascular risk study.

	Original cohort N=16349		Invited to subsample N=578		Participants N=298		Non- participants N=240		p[1]	p[2]	p-[3]
	Mean/N	SD/%	Mean/N	SD/%	Mean/N	SD/%	Mean/N	SD/%			
Birthweight (g)	3578.6	560.8	3552.6	553.6	3554.0	574.7	3551.1	530.8	0.27	0.46	0.95
Gender									0.11	0.05	0.23
Boys	8473	51.9 %	279	48.5	136	46.1	143	46.1 %			
Girls	7866	48.1 %	296	51.5	159	53.9	137	48.9 %			
Ethnicity									0.09	0.50	0.01
Swedish	14235	89.3 %	492	87.1 %	266	90.5 %	226	83.4 %			
Other	1711	10.7 %	73	12.9 %	28	9.5 %	45	16.6 %			
Father ow/ob[a]									0.19	0.54	0.51
Not ow/ob	5384	54.0 %	254	56.4 %	146	57.3 %	108	57.3 %			
Overweight	4003	40.1 %	164	36.4 %	94	36.9 %	70	35.9 %			
Obese	586	5.9 %	32	7.1 %	15	5.9 %	17	8.7 %			
Mother ow/ob[a]									0.95	0.22	0.01
Not ow/ob	7310	70.0 %	328	69.6 %	191	72.9 %	137	65.6 %			

	Overweight	2372	22.7 %	107	22.7 %	59	22.5 %	48	23.0 %			
	Obese	759	7.3 %	36	7.6 %	12	4.6 %	24	11.5 %			
Overweight at 2 yrs										0.46	0.75	0.71
	Not ow/ob	6890	85.0 %	307	83.7 %	187	84.2 %	120	82.8 %			
	Ow/ob	1217	15.0 %	60	16.3 %	35	15.8 %	25	17.2 %			
Overweight at 5 yrs										0.35	<0.01	<0.01
	Not ow/ob	5624	83.3 %	287	85.2 %	187	90.3 %	100	76.9 %			
	Ow/ob	1125	16.7 %	50	14.8 %	20	9.7 %	30	23.1 %			
Overweight at 8 yrs										0.76	0.51	0.10
	Not ow/ob	2566	85.1 %	2566	85.1 %	139	86.9 %	50	78.1 %			
	Ow/ob	450	14.9 %	450	14.9 %	21	13.1 %	14	21.9 %			
Overweight at 12 yrs										0.63	0.20	0.08
	Not ow/ob	2836	84.2 %	197	85.3 %	155	87.6 %	42	77.8 %			
	Ow/ob	534	15.8 %	34	14.7 %	22	12.4 %	12	22,2 %			
Maternal education										< 0.01	< 0.01	<0.01
	High	5062	31.8 %	233	41.3 %	160	54.4 %	73	27.0 %			
	Low	10882	68.2 %	331	58.7 %	134	45.6 %	197	73.0 %	197	73.0 %	
Income										< 0.01	< 0.01	< 0.01
	High	3239	20.0 %	153	26.7 %	114	38.6 %	39	14.0 %			
	Middle	9718	60.0 %	301	52.5 %	141	47.8 %	160	57.6 %			
	Low	3239	20.0 %	119	20.8 %	40	13.6 %	79	28.4 %			

p-value[1]= Invited to follow-up vs original cohort, T-test or Chi2 as appropriate
p-value[2]= Participants in follow-up vs original cohort, T-test or Chi2 as appropriate
p-value[3]=Participants vs non-participants in follow-up, T-test or Chi2 as appropriate
[a] Parental height and weight self-reported at child age 1 year
T-test or Chi2 as appropriate
