# Supplementary material for: Inequalities in cardiovascular risks among Swedish adolescents (ABIS): a prospective cohort study
Source: BMJ Open. 2020 Feb 20;10(2):e030613. doi: 10.1136/bmjopen-2019-030613 (PMC7044991; doi:10.1136/bmjopen-2019-030613)
Supplement: Author's manuscript [file bmjopen-2019-030613.draft_revisions.pdf]

BMJ Open

BMJ Open is committed to open peer review. As part of this commitment we make the peer review history of every article we publish publicly available.

When an article is published we post the peer reviewers' comments and the authors' responses online. We also post the versions of the paper that were used during peer review. These are the versions that the peer review comments apply to.

The versions of the paper that follow are the versions that were submitted during the peer review process. They are not the versions of record or the final published versions. They should not be cited or distributed as the published version of this manuscript.

BMJ Open is an open access journal and the full, final, typeset and author-corrected version of record of the manuscript is available on our site with no access controls, subscription charges or pay-per-view fees (http://bmjopen.bmj.com).

If you have any questions on BMJ Open's open peer review process please email info.bmjopen@bmj.com

BMJ Open

Inequalities in Cardiovascular risks among Swedish adolescents – ABIS a prospective cohort study

Journal:	*BMJ Open*
Manuscript ID	bmjopen-2019-030613
Article Type:	Research
Date Submitted by the Author:	07-Jun-2019
Complete List of Authors:	Andersson White, Pär; Linköping University, Dept. of Medicine and Health; Linköping University Hospital, Crown Princess Victoria Children´s Hospital<br>Ludvigsson, Johnny; Linkopings universitet, Div of Pediatrics, Dept of Clin Exp Medicin, Medical Faculty<br>Jones, Michael; Macquarie University<br>Faresjo, Tomas; Linköpings universitet Hälsouniversitetet
Keywords:	PUBLIC HEALTH, EPIDEMIOLOGY, Cardiac Epidemiology < CARDIOLOGY, Paediatric endocrinology < DIABETES & ENDOCRINOLOGY, SOCIAL MEDICINE

SCHOLARONE™
Manuscripts

I, the Submitting Author has the right to grant and does grant on behalf of all authors of the Work (as defined in the below author licence), an exclusive licence and/or a non-exclusive licence for contributions from authors who are: i) UK Crown employees; ii) where BMJ has agreed a CC-BY licence shall apply, and/or iii) in accordance with the terms applicable for US Federal Government officers or employees acting as part of their official duties; on a worldwide, perpetual, irrevocable, royalty-free basis to BMJ Publishing Group Ltd ("BMJ") its licensees and where the relevant Journal is co-owned by BMJ to the co-owners of the Journal, to publish the Work in this journal and any other BMJ products and to exploit all rights, as set out in our licence.

The Submitting Author accepts and understands that any supply made under these terms is made by BMJ to the Submitting Author unless you are acting as an employee on behalf of your employer or a postgraduate student of an affiliated institution which is paying any applicable article publishing charge ("APC") for Open Access articles. Where the Submitting Author wishes to make the Work available on an Open Access basis (and intends to pay the relevant APC), the terms of reuse of such Open Access shall be governed by a Creative Commons licence – details of these licences and which Creative Commons licence will apply to this Work are set out in our licence referred to above.

Other than as permitted in any relevant BMJ Author's Self Archiving Policies, I confirm this Work has not been accepted for publication elsewhere, is not being considered for publication elsewhere and does not duplicate material already published. I confirm all authors consent to publication of this Work and authorise the granting of this licence.

For peer review only

Inequalities in Cardiovascular risks among Swedish adolescents – ABIS a prospective cohort study

Pär Andersson White[1, 2], Johnny Ludvigsson[2, 3], Mike Jones[4], Tomas Faresjö[1]

[1]Dept. of Medicine and Health, Div. of Community Medicine, Linköping University, Sweden.

[2]Crown Princess Victoria Children´s Hospital, Region Östergötland, Linköping, Sweden.

[3]Div. of Pediatrics, Dept. of Clinical and Experimental Medicine, Linköping University, Sweden.

[4]Psychology Department, Macquarie University, North Ryde, NSW, 2109, Australia.

Corresponding author: Pär Andersson White, C/O T Faresjo, Dept. of Medicine and Health, Div. of Community Medicine, Linköping University, SE-581 83 Linköping Sweden, +4610-1030000, par.andersson.white@liu.se

Keywords: Socioeconomic status, Inequalities, Adolescent Overweight, Childhood overweight, Childhood obesity, Blood Pressure, Dyslipidemia, High-Density Lipoproteins, Low-Density Lipoproteins

For peer review only - http://bmjopen.bmj.com/site/about/guidelines.xhtml

For peer review only

Abstract

OBJECTIVES: To investigate if socioeconomic differences are related to indicators of cardiovascular risk such as blood pressure, blood lipids and overweight among Swedish adolescents and if so at what age for overweight and obesity.

DESIGN: Longitudinal follow-up of a prospective birth cohort.

SETTING: All Babies in Southeast Sweden (ABIS) study include data from children born between October 1997 and October 1999 in five counties of south east Sweden. The last follow-up is a sub-sample of ABIS-participators in three major cities of the region.

PARTICIPANTS: A regional subsample n=298 adolescents aged 16-18 years from the ABIS-study, and prospective date from the whole ABIS cohort (n= 2998-7925) for overweight and obesity status at the ages 2, 5, 8 and 12 years.

OUTCOME MEASURES: Blood pressure above the age and length adjusted limit for hypertension, overweight according to the International Obesity Task Force (IOTF) definition, low high-density lipoproteins (HDL) or borderline-high low-density lipoproteins (LDL) according to National Cholesterol Education Program (NCEP) expert panel on cholesterol levels in children.

RESULTS: For three out of four cardiovascular risk outcomes there were significantly increased risk in Low Socioeconomic Status (SES) groups. The SES variables with best prediction of the outcomes measured were Swedish socio-economic classification (SEI) for elevated blood pressure, maternal educational level for overweight and blue-collar city of residence for low high-density lipoprotein (HDL). SES related differences in overweight were found at age 2, 5 and 12 and for obesity at age 2, 5, 8, and 12.

For peer review only

For peer review only - http://bmjopen.bmj.com/site/about/guidelines.xhtml

CONCLUSIONS: Even in a welfare state like Sweden, SES inequalities in cardiovascular risks is evident already in childhood and adolescence. Intervention programs should start early in life.

Strengths and limitations of this study:

- Data derives from a large prospective cohort representative for the general population.
- A standardized procedure for collection of clinical data. The same nurse has done all blood pressure measurements, taken all blood samples and collected data on weight and height.
- The Swedish registers give us valid and reliable data on socioeconomic factors
- The limited sample size of the follow–up group gives some restraints

For peer review only

Introduction

Cardiovascular disease in adults has been shown to be one of the disease groups most correlated with socioeconomic inequalities in the world [1], and this has also been evident in Swedish adults [2]. Longitudinal studies have shown that risk factors for cardiovascular disease develop in childhood and that early signs of cardiovascular disease risks are visible in early adulthood [3]. The risk factors associated with cardiovascular disease includes blood pressure, dyslipidemia and overweight. Of these risk factors, overweight and obesity are the ones that progress most consistently from childhood to adulthood [4]. Adolescent overweight has been shown to be a stronger predictor of cardiovascular mortality in adulthood than adult obesity [5].

Recent studies report lower scores than ideal in a range of cardiovascular health factors (blood pressure, cholesterol and glucose level below the American heart association's cut-offs) in European adolescents with low maternal education [6]. In Finland low socioeconomic status measured by occupation of the father was associated with higher low-density lipoproteins (LDL) levels in boys and higher systolic blood pressure and higher percentage of smokers [7]. However, longitudinal data on the relationships between childhood socioeconomic status (SES) and adolescent's cardiovascular health is still scarce.

An increase in physical activity has been shown to reduce the risk of being overweight and obese as a youth in Sweden and other European countries [8]. Studying the effect of diet on development of overweight and obesity in children and adolescents has yielded no clear conclusions on whether any specific foods are important for the development of obesity [9]. However there is some evidence for intake of certain foods and development of adult

For peer review only - http://bmjopen.bmj.com/site/about/guidelines.xhtml

cardiovascular disease. The INTERHEART study found a positive association between intake of meat, salty foods, fried foods and development of cardiovascular disease and a negative association between intake of fruits and vegetables and cardiovascular disease risk [10].

Previous studies suggest that cardiovascular disease often starts early in life [3 11 12]. The early risk factors that lead to cardiovascular disease have been shown in some populations to be more prevalent in the low SES groups [13 14]. Although Sweden has dropped in the Organization for Economic Co-operation and Development (OECD) list of economic equality from 1st place in the 1980ies to 9th in 2016 [15], Sweden still has a comprehensive public health system with a number of interventions aimed at reducing social inequalities. These include universal and public financed healthcare equally accessible for all. This includes free antenatal consulting and check-ups at Child Health Care Centers which covers advice on nutrition and health from professional nurses for all children at regular intervals up to school age. Other interventions include school-health check-ups and mandatory physical activity in schools and free school lunches for all children. Other support for families with children include paid maternity leave for 390 days and heavily subsidized daycare. Public health interventions include encouragement of attendance at antenatal and postnatal clinics, support of breastfeeding, as well as taxes deliberately aimed at reducing tobacco and alcohol consumption [16].

Aims

The aims of this study were to determine whether social inequalities are related to cardiovascular risk factors including blood pressure, lipids and overweight in Swedish adolescents, and if so, to identify which socioeconomic factors that were most important for the development of each risk factor, and from what age socioeconomic differences in

For peer review only - http://bmjopen.bmj.com/site/about/guidelines.xhtml

For peer review only

overweight and obesity occur using longitudinal data from follow-ups in the ABIS prospective study.

Material and Methods

Study population

The ABIS study (All Babies In southeast Sweden) is a prospective population-based cohort study, initially including 17 055 children born between October 1st, 1997 and October 1st 1999 in Southeast of Sweden in the counties of Östergötland, Jönköping, Kronoberg, Kalmar, Blekinge and Öland. This cohort of children has been followed from birth and are still being followed with respect to biological tests, questionnaires and register data. The initial aim of the ABIS-study was to analyse the development of immune-mediated diseases including autoimmune diseases, type 1 and type 2 diabetes as well as cardiovascular disease, and the effect of different environmental and genetic factors.

We undertook a follow-up of a regional subsample of ABIS-children and also a questionnaire-based longitudinal analysis for the whole cohort. Inclusion criteria for the subsample were age 16-18 years, previous participation in the ABIS-study and living in one of the three major cities of Östergötland County. Out of n=578 children invited n=298 (51.6%) participated. The invited subsample was similar to the original ABIS cohort in respect to a number of variables (birthweight, ethnicity, parental overweight and obesity, child overweight at age 2, 8 and 12 years). However, analysis showed a significantly higher proportions of mothers with high education and families with high income, a lower number with overweight at 5 years and a slight differences (p=0.052) in gender composition with

For peer review only - http://bmjopen.bmj.com/site/about/guidelines.xhtml

boys being underrepresented in the subsample The main reasons for drop-outs among the invited sample were practical i.e. difficulties to arrange health check-ups for our research nurse at some schools that lacked school-nurse facilities. Drop-out analysis also showed evidence of underrepresentation of children with foreign ethnicity, low maternal education and low household income (see supplementary table 1). The participants in our follow-up had a mean age of 16.05 (SD 0.9) years (n=136 males, n=162 females). They were distributed between the cities of Linköping n=160 (54%), Norrköping n=91 (30%) and Motala n=47 (16%). These cities represent different kinds of social environments. The participants from Motala and Norrköping, two towns with a history of heavy industries, were merged together in the analysis and labelled as blue-collar cities. Linköping which is the administrative centre of the county and has one of Sweden's major universities was labelled white-collar city. The study participants filled out a questionnaire about their lifestyle, diet and physical activity. A specially trained research nurse undertook all data collection including blood pressure measurement, questionnaire and collection of blood samples and measurement of anthropometric data.

Questionnaire

The questionnaire included questions concerning social conditions, diet and physical activity. A cardiovascular risk diet index (CRD) was constructed based on the dietary components most associated with myocardial infarction in the INTERHEART study [10]. These factors were: intake of salty foods one time per day or more, intake of deep fried foods three times or more per week, intake of meat two or more times per day, intake of fruit less than once per day and intake of vegetables less than once per day. This score which has a

For peer review only - http://bmjopen.bmj.com/site/about/guidelines.xhtml

range of 0 to 5 was then dichotomized into two groups Low CRD (0-1 items n=101) and High CRD (2 to 5 items n=138).

Degree of physical activity was measured by the question "How many times per week do you do physical activity at least 30 min that is strenuous enough to make you sweat". The answers were then grouped into two groups; 0-2 times a week, >2 times a week.

Blood Pressure

Systolic and diastolic blood pressure was measured manually by the research nurse at the participant's school nursing offices. Study participants rested on a bed at least 5 minutes before measurement, two consecutive measurements of blood pressure were then taken twice in the sitting position and the mean value was calculated. Blood pressures were grouped as hyper- or normotensive according to the guidelines described in the fourth report on the diagnosis, evaluation, and treatment of high blood pressure in children and adolescents [17]. These guidelines took into consideration the adolescents' age in months and height percentile according to the Center of Disease Control and Prevention growth charts [18].

Anthropometrical factors

The participating adolescents' height (cm) and weight (kg) were measured by the research nurse. Data on weight and height were missing for 8 participants. Obesity and body mass index (BMI) was calculated as BMI = weight(kg)/height(m)^2 and dichotomized as overweight/obese or not, using the International obesity task force latest age and gender specific cut-offs defined by Cole et al [19 20] The same procedure was then used for parental-

For peer review only - http://bmjopen.bmj.com/site/about/guidelines.xhtml

reported height and weight measures available from previous ABIS follow-ups at age 2, 5, 8 and 12 years.

Dyslipidemia

Blood samples for blood lipid analysis were collected from 242 (81.2 %) individuals and analyzed for plasma levels of LDL and HDL at the Department of clinical chemistry, Linköping University Hospital, according to accredited procedures. One sample further was missing for LDL after analysis. Test results were converted from SI units to mg/dL. Results were then grouped as normal or low HDL cholesterol in accordance with the cut-off for low HDL cholesterol (<40 mg/dL) set by the Expert panel on integrated guidelines for cardiovascular health and risk reduction in children, which is based on the 75th percentile found by the National cholesterol educational program [21 22]. For LDL only 8 (3.3%) out of 241 had a level above the limit of high LDL. As this number was insufficient for statistical analyses the cut-off of borderline high LDL (>110 mg/dL) was chosen as indicator of increased LDL.

Socioeconomic variables

A five grade Socioeconomic Class variable was constructed based on the criteria used by Swedish Statistics in their Swedish socioeconomic classification index (SEI) [23]. The adolescents were classified according to the highest occupational class of either of the parents. Mother's educational level was derived from the ABIS birth questionnaire and grouped as primary/secondary education or post-secondary education/university. Family disposable income was derived from Statistics Sweden for the year 2012 and grouped into three groups; low income representing the bottom 20 % of disposable incomes in the original cohort (available for n=16196), middle income representing the middle 20 to 80 percentile of income and top income representing the top 20 percent of income.

For peer review only - http://bmjopen.bmj.com/site/about/guidelines.xhtml

White- collar/Blue-collar city

The participants of this ABIS follow-up were recruited from the county of Östergötland. Two cities (Norrköping, Motala) was defined as Blue-collar cities and one (Linköping) was defined as White-collar [24].

Potential confounders

Birthweight was registered in ABIS in the original birth questionnaire. Ethnicity was based on the question "Are you (the mother) born in Sweden" and "Is the father born in Sweden". The adolescents were then grouped as having foreign ethnicity if one of the parents was born outside of Sweden. 24 (9.7 %) out of 248 were categorized as having foreign ethnicity.

Patient and Public Involvement

Patients and public were not involved in the design of this study. The results will be disseminated to the study participants through the ABIS study homepage https://www.abis-studien.se/.

Statistical analysis

Data were analyzed using bivariate logistic regression for each cardiovascular risk factor. As analysis of missing values showed significant differences due to socioeconomic status, adolescents with low maternal educational level had a significantly higher number of missing values in questionnaire data, multiple imputation was used for missing values to reduce this potential bias. A univariate logistic regression was then analyzed for each independent variable and Odds Ratios (OR) with 95% confidence interval were calculated. For further analysis all four SES variables were included on a priori basis while other independent variables were chosen based on reaching statistical significance $p \leq 0.05$ or an

For peer review only - http://bmjopen.bmj.com/site/about/guidelines.xhtml

effect size of OR <0.5 or >2.0 for categorical variables. For continuous variables; height, age and birthweight was standardized and the OR of 1 standard deviation increase of <0.75 or >1.5 was used as selection criteria. This approach was adopted due to limited sample size and, in this context, effect size being as important from a public health perspective as statistical significance.

The degree of discrimination afforded by each socioeconomic variable was quantified by the area under the receiver operating characteristic (ROC) curve defined by taking the logistic model predicted probability of adverse health state as the test measure and the actual state as an imperfect reference standard. Values >0.5 indicate some discrimination with values close to 1.0 indicating excellent discrimination.

A hierarchical approach was adopted in which SES variables were first adjusted for other SES variables, then for other lifestyle factors and finally for adolescent's overweight status.

Bivariate logistic regression was also used in the final analysis on the relationship between overweight/obesity and maternal educational level at four different time points. The first analysis used a cohort approach and included only subjects with complete data from all follow-ups (n = 1070). The second analysis used an available sample approach and included all subjects that had data for any specific age (n=2995-7925).

Statistical analysis was performed using Statistical Package for the Social Sciences v 23 (SPSS, Chicago, IL, USA)

Ethical considerations

The ABIS-study has been approved by the Research Ethics committees of the Faculty of Health Science at Linköping University. Dnr 03-092, Dnr 99-227 and Dnr 99-321. All

For peer review only - http://bmjopen.bmj.com/site/about/guidelines.xhtml

For peer review only

participants in the follow-up gave their informed consent as well as their care-givers for those below 18 years of age.

Results

Blood pressure

The total number of participants that were classified having elevated blood pressure was 49/290 (16.9 %), see table 1.

This was based on 34/290 participants (11.7%) with systolic blood pressures above the limit for hypertension and 17/290 (5.9 %) with diastolic blood pressure above the limit. Two individuals were above both systolic and diastolic hypertension limits.

All SES variables were associated with increased odds ratios (OR) for elevated blood pressure in the lowest SES groups compared with the highest, see table 2. Out of the four SES variables, parental occupation according to SEI had the highest area under the ROC curve, 0.623 compared to 0.537-0.578 for the other SES variables. The lowest social class had an OR of 4.37 (95% confidence interval 1.49, 12.81) compared to the highest class.

Other variables that reached the effect size of an OR of <0.5 or >2 were low physical activity 0-2 times/week which had an OR of 0.39 (95% confidence interval 0.15, 1.05) and overweight status with OR 4.42 (95% confidence interval 2.19, 8.95).

After adjustments for other SES variables, physical activity and overweight status the lowest SEI class had an OR for elevated blood pressure of 4.95 (95% confidence interval 1.30, 18.89), see table 3.

Dyslipidemia

For peer review only - http://bmjopen.bmj.com/site/about/guidelines.xhtml

An HDL level below 40 mg/dL was found in 30 (10.1 %) out of 242 samples. All indicators of low SES showed an increased odd ratio of having low HDL. Out of the four variables, Blue-collar city was associated with the highest area under the ROC curve, 0.652 compared to 0.540-0.627 for the other SES variables. Blue-collar city was associated with an OR of 1.78 (95% confidence interval 0.74, 4.31) of low HDL. Low maternal education and low income were both associated with significantly increased risks of low HDL, low maternal education with an OR of 2.39 (95% confidence interval 1.10, 5.21) and low income with an OR of 3.94 (95% confidence interval 1.39, 11.14) for having low HDL.

Other variables that reached the effect size of OR <0.5 or >2 for low HDL were; gender where boys carried an OR of 2.78 (95% confidence interval 1.09, 7.09), cardiovascular risk diet where high CRD had an OR of 2.41 (95% confidence interval 0.95, 6.15) and overweight with an OR 3.44 (95% confidence interval 1.47, 8.07).

After adjustment for other SES variables, gender, CRD and overweight the OR for Blue-collar city was reduced to 1.09 (95% confidence interval 0.40, 2.93).

For LDL 25/241 (10.4 %) were above the cut-off for borderline-high value (110 mg/dL). The difference between low and high SES groups was small, and no SES variable reached an OR above 2.0, and no variable reached statistical significance $p \leq 0.05$. The area under the ROC curve ranged from 0.573 for SEI to 0.511 for White collar/Blue-collar city.

Overweight

In the regional subsample 46/290 (15.8 %) were overweight or obese, with 7 (2.4 %) obese. All four measures of low SES had an increased odds ratio of being overweight or obese. The best discriminator measured as area under the ROC curve for a single SES variable was 0.641

For peer review only - http://bmjopen.bmj.com/site/about/guidelines.xhtml

for maternal education. The only other variable that met the criteria for being included in the multivariate analysis was diet according to CRD.

After adjustment for CRD and other SES variables maternal education had an odds ratio of 2.39 (95% confidence interval 1.08, 5.30) for being overweight.

Overweight and obesity inequality during childhood

Overweight and obesity differences related to maternal education were measured longitudinally at four different time-points. In the analysis on subjects with complete data (cohort approach) the children to low educated mothers´ had significantly increased odds of being overweight at age 12, but not at age 2, 5 and 8. The analysis based on all available data (available sample approach) showed significant differences at age 2, 5 and 12 but not at age 8. For obesity there were significant differences at age 5 and 12 in the cohort approach, while the available sample approach showed significant differences at all ages, 2, 5, 8 and 12 years, see table 4.

Discussion

There was significantly increased risk of three out of four cardiovascular risk factors due to low SES in Swedish adolescents. Parental occupational class according to SEI was the most important factor for risk of elevated blood pressure. Although overweight was important for elevated blood pressure the independent importance of parental occupational class was further strengthened as it remained after adjustment for other SES factors, physical activity and overweight. For other outcomes we found maternal education to be the best discriminator for overweight, and white/blue collar city for low HDL (<40 mg/dL).

For peer review only - http://bmjopen.bmj.com/site/about/guidelines.xhtml

For peer review only

In the Whitehall studies, occupational class and blood pressure were found to be closely associated in adults [25]. One finding of the Whitehall II study is that workers with low occupational grade have increased blood pressure during the working day, but no difference in resting blood pressure [26]. As blood pressure in this study was measured while the adolescents' were attending school it is possible that it represents the same kind of increase in response to school tasks as that seen during the work day in adult workers.

The impact of maternal education on child overweight has been shown in large multi cohort studies [27]. In addition to being a marker for socioeconomic status like occupation and income the impact of maternal education could involve differences in knowledge. There is evidence that adolescents with mothers with good nutritional knowledge have a healthier diet [28]. High maternal education has also been associated with a higher degree of breakfast eating in adolescents [29]. Further, low parental education has been associated with increased access to sugar sweetened drinks and low vegetable intake [30]. These factors could be part of the explanation of why maternal educational level is more important than other SES factors for overweight status in adolescents.

Blue-collar city was the best discriminator of low HDL status. After adjustment for other SES variables the odds ratio for blue-collar cities was reduced to OR 1.20 (0.46, 3.12), adjusting for diet and overweight further decreased the OR to 1.09 (0.40, 2.93) indicating that most of the risk associated with Blue-collar residence is due to differences in SES, diet and overweight. Thus our results are in line with previous studies [31].

The main limitation of this study is the limited number of participants of the follow-up part (n=298). Even though the sample size of study is somewhat restrained we managed to capture socioeconomic related significant differences in cardiovascular risks. Our sample is

For peer review only - http://bmjopen.bmj.com/site/about/guidelines.xhtml

quite representative concerning overweight status compared to the previous larger samples in ABIS and to other Swedish studies [32]. Although the number of low income families were similar in our invited subsample compared to the original cohort there was an overrepresentation of high maternal education and high income families, this most likely reflects the fact that the subsample is from a more urban population than the rest of the ABIS region and must be taken into consideration when interpreting the result. Among the invited, the number of adolescents with high maternal education at birth and high income families were higher among the participants of the study than in the non-participant group the same was true for high income families. This is a common finding in longitudinal studies, but as previous studies have shown, the effect of this underrepresentation in low SES groups, if anything, is to underestimate inequality slightly in later follow-ups and have little effect on the qualitative conclusion on inequality [33]. The number of adolescents with elevated blood pressure was quite high (16.9%) and as blood pressure was measured only at one visit to school by the research nurse some of the adolescents might have had White-coat rather than true hypertension [17]. However, other studies have shown similar high values in other populations [34]. A recent study in a Swedish cohort also found higher average systolic blood pressure in the age group 14-16 years than was found in the fourth report on the diagnosis, evaluation, and treatment of high blood pressure in children and adolescents [35]. We could not rule out that White-coat hypertension might be related to socioeconomic factors, but have no evidence of that.

The fact that the longitudinal data was based on parental-reported height and weight might affect the accuracy of our results. A German study found that parental reported data tends to overestimate the prevalence of overweight in young children and underestimate it in older children, however the same study found the impact of socioeconomic status on this

For peer review only - http://bmjopen.bmj.com/site/about/guidelines.xhtml

error to be small.[36] Thus, although the exact prevalence of overweight status in our study might differ slightly from the true prevalence the relative difference between SES groups should not.

Different socioeconomic variables can have different effects on health. It is a strength that we investigated not one but four dimensions of socioeconomic status inequality. These dimensions seem to be of varying importance for cardiovascular risk outcomes. Thus our study is an example of the many layers of the social determinants of health that give rise to an individual's risk of disease [37]. Although income, maternal education and occupational status are correlated, it is not surprising that the strongest discriminators for cardiovascular risk factors are occupational class, blue collar city and educational level rather than income in this Swedish cohort, as income inequality still is low compared to most other countries [38]. However, our results show that socioeconomic differences in overweight and obesity could be found already in early childhood also in Sweden.

The implication of adolescent SES differences on adult life cardiovascular health inequalities could be important. Longitudinal data from Finland have shown that cardiovascular risk factors such as elevated blood pressure, serum lipids and obesity tracks to adulthood, increasing the risk of adult abnormalities [39]. Data from the same cohort also shows that adolescent blood pressure and LDL cholesterol are risk factors of adult carotid intima media thickening independent of adult risk factors [40]. A general awareness in primary care is important of the potential risk of elevated blood pressure and other cardiovascular risks in low SES adolescents.

Sweden has for a long time been considered one of the more egalitarian countries in the world and has often been used for comparison in studies on health inequalities as a good

For peer review only - http://bmjopen.bmj.com/site/about/guidelines.xhtml

For peer review only

example [41 42]. Yet during the last two decades this has started to change with economic inequality increasing faster than in other OECD countries and Sweden has gone from a top position in equality to now being the 9th country in income equality out of the 36 countries in OECD [15]. Previous studies have found that for health problems with higher prevalence in low SES groups, like cardiovascular disease in adults, both the over-all prevalence and absolute inequality between high and low SES groups increases with larger economic inequality [1 43]. Our finding that cardiovascular risks in children and adolescents have a SES gradient also in Sweden could have serious public health implication for the future, as the rapid rise of economic inequality might increase absolute inequalities and prevalence of cardiovascular risk factors in children and adolescents. Concerns for health inequalities have increased in recent years spurring the Swedish government to start a Commission for health equality with the aim of finding ways to decrease these inequalities [44].

Conclusion

Despite an extensive welfare system and public health interventions in Sweden, cardiovascular health inequality is evident among Swedish youths and children. Interventions to improve cardiovascular health in children should pay special attention to social inequalities. Policies aimed at decreasing the evident inequalities in cardiovascular disease in adults should start early and include interventions already in childhood and adolescence.

For peer review only

For peer review only - http://bmjopen.bmj.com/site/about/guidelines.xhtml

Funding

The ABIS-study has been supported by Swedish Research Council (K2005-72X-11242-11A and K2008-69X-20826-01-4) and the Swedish Child Diabetes Foundation (Barndiabetesfonden), JDRF Wallenberg Foundation (K 98-99D-12813-01A), Medical Research Council of Southeast Sweden (FORSS) and the Swedish Council for Working Life and Social Research (FAS2004–1775) and Östgöta Brandstodsbolag. The Research and PhD studies Committee (FUN), Linköping University, Sweden (LiU-).

Competing interests

None declared.

Data sharing statement:

Data can be accessed via the Dryad data repository at http:// datadryad. org/.

Author Contributions

TF, JL and PAW were responsible for the study concept and design. JL is the principal investigator for ABIS. PAW did the statistical analysis with advice from MJ. All authors were responsible for analysis and interpretation of data. PAW drafted the manuscript and TF, JL and MJ undertook critical revision of the manuscript. PAW has full access to all of the data in the study, takes responsibility for the integrity of the data and accuracy of the data analysis and had the final responsibility to submit for publication.

For peer review only

References

1. Wilkinson RG, Pickett KE. Income inequality and socioeconomic gradients in mortality. *Am J Public Health* 2008;98(4):699-704. doi: 10.2105/AJPH.2007.109637
2. Toch-Marquardt M, Menvielle G, Eikemo TA, et al. Occupational class inequalities in all-cause and cause-specific mortality among middle-aged men in 14 European populations during the early 2000s. *PLoS One* 2014;9(9):e108072. doi: 10.1371/journal.pone.0108072
3. Berenson GS. Childhood risk factors predict adult risk associated with subclinical cardiovascular disease. The Bogalusa Heart Study. *Am J Cardiol* 2002;90(10C):3L-7L.
4. Srinivasan SR, Bao W, Wattigney WA, et al. Adolescent overweight is associated with adult overweight and related multiple cardiovascular risk factors: the Bogalusa Heart Study. *Metabolism* 1996;45(2):235-40.
5. Must A, Jacques PF, Dallal GE, et al. Long-term morbidity and mortality of overweight adolescents. A follow-up of the Harvard Growth Study of 1922 to 1935. *N Engl J Med* 1992;327(19):1350-5. doi: 10.1056/NEJM199211053271904
6. Henriksson P, Henriksson H, Labayen I, et al. Correlates of ideal cardiovascular health in European adolescents: The HELENA study. *Nutrition, metabolism, and cardiovascular diseases : NMCD* 2018;28(2):187-94. doi: 10.1016/j.numecd.2017.10.018
7. Leino M, Porkka KV, Raitakari OT, et al. Influence of parental occupation on coronary heart disease risk factors in children. The Cardiovascular Risk in Young Finns Study. *Int J Epidemiol* 1996;25(6):1189-95.
8. Janssen I, Katzmarzyk PT, Boyce WF, et al. Comparison of overweight and obesity prevalence in school-aged youth from 34 countries and their relationships with physical activity and dietary patterns. *Obes Rev* 2005;6(2):123-32. doi: 10.1111/j.1467-789X.2005.00176.x
9. Pate RR, O'Neill JR, Liese AD, et al. Factors associated with development of excessive fatness in children and adolescents: a review of prospective studies. *Obes Rev* 2013;14(8):645-58. doi: 10.1111/obr.12035
10. Iqbal R, Anand S, Ounpuu S, et al. Dietary patterns and the risk of acute myocardial infarction in 52 countries: results of the INTERHEART study. *Circulation* 2008;118(19):1929-37. doi: 10.1161/circulationaha.107.738716 [published Online First: 2008/10/22]
11. Mahoney LT, Burns TL, Stanford W, et al. Coronary risk factors measured in childhood and young adult life are associated with coronary artery calcification in young adults: the Muscatine Study. *Journal of the American College of Cardiology* 1996;27(2):277-84. [published Online First: 1996/02/01]
12. Puolakka E, Pahkala K, Laitinen TT, et al. Childhood Socioeconomic Status and Arterial Stiffness in Adulthood: The Cardiovascular Risk in Young Finns Study. *Hypertension* 2017;70(4):729-35. doi: 10.1161/HYPERTENSIONAHA.117.09718
13. Seo YG, Choi MK, Kang JH, et al. Cardiovascular disease risk factor clustering in children and adolescents: a prospective cohort study. *Archives of disease in childhood* 2018;103(10):968-73. doi: 10.1136/archdischild-2017-313226 [published Online First: 2018/04/14]
14. Jabakhanji SB, Boland F, Ward M, et al. Body Mass Index Changes in Early Childhood. *The Journal of pediatrics* 2018;202:106-14. doi: 10.1016/j.jpeds.2018.06.049
15. OECD. Key indicators on the distribution of household disposable income and poverty, 2007, 2015 and 2016 or most recent year [Available from: http://www.oecd.org/social/soc/IDD-Key-Indicators.xlsx].
16. Wettergren B, Blennow M, Hjern A, et al. Child Health Systems in Sweden. *The Journal of pediatrics* 2016;177s:S187-s202. doi: 10.1016/j.jpeds.2016.04.055 [published Online First: 2016/09/27]

For peer review only - http://bmjopen.bmj.com/site/about/guidelines.xhtml

17. National High Blood Pressure Education Program Working Group on High Blood Pressure in C, Adolescents. The fourth report on the diagnosis, evaluation, and treatment of high blood pressure in children and adolescents. *Pediatrics* 2004;114(2 Suppl 4th Report):555-76.
18. Gibbs BG, Forste R. Socioeconomic status, infant feeding practices and early childhood obesity. *Pediatr Obes* 2014;9(2):135-46. doi: 10.1111/j.2047-6310.2013.00155.x
19. Cole TJ, Bellizzi MC, Flegal KM, et al. Establishing a standard definition for child overweight and obesity worldwide: international survey. *BMJ (Clinical research ed)* 2000;320(7244):1240-3.
20. Cole TJ, Lobstein T. Extended international (IOTF) body mass index cut-offs for thinness, overweight and obesity. *Pediatr Obes* 2012;7(4):284-94. doi: 10.1111/j.2047-6310.2012.00064.x
21. National Cholesterol Education Program (NCEP): highlights of the report of the Expert Panel on Blood Cholesterol Levels in Children and Adolescents. *Pediatrics* 1992;89(3):495-501.
22. Expert Panel on Integrated Guidelines for Cardiovascular H, Risk Reduction in C, Adolescents, et al. Expert panel on integrated guidelines for cardiovascular health and risk reduction in children and adolescents: summary report. *Pediatrics* 2011;128 Suppl 5:S213-56. doi: 10.1542/peds.2009-2107C
23. Wijlaars LP, Johnson L, van Jaarsveld CH, et al. Socioeconomic status and weight gain in early infancy. *Int J Obes (Lond)* 2011;35(7):963-70. doi: 10.1038/ijo.2011.88
24. Wennerholm C, Grip B, Johansson A, et al. Cardiovascular disease occurrence in two close but different social environments. *Int J Health Geogr* 2011;10:5. doi: 10.1186/1476-072X-10-5
25. Ferrie JE, Shipley MJ, Davey Smith G, et al. Change in health inequalities among British civil servants: the Whitehall II study. *Journal of epidemiology and community health* 2002;56(12):922-6.
26. Steptoe A, Kunz-Ebrecht S, Owen N, et al. Socioeconomic status and stress-related biological responses over the working day. *Psychosomatic medicine* 2003;65(3):461-70. [published Online First: 2003/05/24]
27. Ruiz M, Goldblatt P, Morrison J, et al. Impact of Low Maternal Education on Early Childhood Overweight and Obesity in Europe. *Paediatr Perinat Epidemiol* 2016;30(3):274-84. doi: 10.1111/ppe.12285
28. Tabbakh T, Freeland-Graves JH. The home environment: A mediator of nutrition knowledge and diet quality in adolescents. *Appetite* 2016;105:46-52. doi: 10.1016/j.appet.2016.05.002 [published Online First: 2016/05/14]
29. Gebremariam MK, Henjum S, Hurum E, et al. Mediators of the association between parental education and breakfast consumption among adolescents : the ESSENS study. *BMC pediatrics* 2017;17(1):61. doi: 10.1186/s12887-017-0811-2 [published Online First: 2017/02/24]
30. Gebremariam MK, Lien N, Torheim LE, et al. Perceived rules and accessibility: measurement and mediating role in the association between parental education and vegetable and soft drink intake. *Nutrition journal* 2016;15(1):76. doi: 10.1186/s12937-016-0196-3 [published Online First: 2016/08/18]
31. Faresjo T, Rahmqvist M. Educational level is a crucial factor for good perceived health in the local community. *Scand J Public Health* 2010;38(6):605-10. doi: 10.1177/1403494810374676
32. Angbratt M, Ekberg J, Walter L, et al. Prediction of obesity from infancy to adolescence. *Acta Paediatr* 2011;100(9):1249-52. doi: 10.1111/j.1651-2227.2011.02326.x
33. Howe LD, Tilling K, Galobardes B, et al. Loss to follow-up in cohort studies: bias in estimates of socioeconomic inequalities. *Epidemiology* 2013;24(1):1-9. doi: 10.1097/EDE.0b013e31827623b1
34. Kagura J, Adair LS, Pisa PT, et al. Association of socioeconomic status change between infancy and adolescence, and blood pressure, in South African young adults: Birth to Twenty Cohort. *BMJ Open* 2016;6(3):e008805. doi: 10.1136/bmjopen-2015-008805

For peer review only

For peer review only - http://bmjopen.bmj.com/site/about/guidelines.xhtml

35. Krmar RT, Holtback U, Bergh A, et al. Oscillometric casual blood pressure normative standards for Swedish children using ABPM to exclude casual hypertension. *Am J Hypertens* 2015;28(4):459-68. doi: 10.1093/ajh/hpu182
36. Brettschneider AK, Ellert U, Schaffrath Rosario A. Comparison of BMI derived from parent-reported height and weight with measured values: results from the German KiGGS study. *International journal of environmental research and public health* 2012;9(2):632-47. doi: 10.3390/ijerph9020632 [published Online First: 2012/04/04]
37. Commission on Soci l Determinants of Health. CSDH Final Report: Closing the Gap in a Generation: Health Equity Through Action on the Social Determinant of Health. . Geneva, Switzerland: : World Health Organization, 2008.
38. OECD. In It Together: Why Less Inequality Benefits All: OECD Publishing.
39. Juhola J, Magnussen CG, Viikari JS, et al. Tracking of serum lipid levels, blood pressure, and body mass index from childhood to adulthood: the Cardiovascular Risk in Young Finns Study. *The Journal of pediatrics* 2011;159(4):584-90. doi: 10.1016/j.jpeds.2011.03.021 [published Online First: 2011/04/26]
40. Raitakari OT, Juonala M, Kahonen M, et al. Cardiovascular risk factors in childhood and carotid artery intima-media thickness in adulthood: the Cardiovascular Risk in Young Finns Study. *Jama* 2003;290(17):2277-83. doi: 10.1001/jama.290.17.2277 [published Online First: 2003/11/06]
41. Leon DA, Vagero D, Olausson PO. Social class differences in infant mortality in Sweden: comparison with England and Wales. *BMJ (Clinical research ed)* 1992;305(6855):687-91.
42. Kunst AE, Mackenbach JP. The size of mortality differences associated with educational level in nine industrialized countries. *Am J Public Health* 1994;84(6):932-7.
43. Pickett KE, Wilkinson RG. Child wellbeing and income inequality in rich societies: ecological cross sectional study. *BMJ (Clinical research ed)* 2007;335(7629):1080. doi: 10.1136/bmj.39377.580162.55 [published Online First: 2007/11/21]
44. Lundberg O. The next stop towards more equity in health in Sweden – How can we close the gap in a generation? 2017 [Available from: http://kommissionjamlikhalsa.se/en/].

For peer review only

For peer review only - http://bmjopen.bmj.com/site/about/guidelines.xhtml

Table 1. Characteristics of adolescents by hypertension and overweight status.

	Normal blood pressure		Elevated blood pressure		Not overweight		Overweight	
	N	%	N	%	N	%	N	%
Total	240	83.0	49	17.0	244	84.1	46	15.9
Sex								
Boy	105	43.8	27	55.1	109	44.7	24	52.2
Girl	135	56.2	22	44.9	135	55.3	22	47.8
Ethnicity								
Swedish	213	89.9	44	91.7	218	90.5	40	88.9
Foreign ethnicity	24	10.1	4	8.3	23	9.5	5	11.1
Physical activity								
– times per week								
>2	138	67.6	31	83.8	149	72.0	21	60.0
0-2	66	32.4	6	16.2	58	28.0	14	40.0
CRD								
0-1 item	88	44.4	14	40.0	94	46.5	8	25.0
2-5 items	110	55.6	21	60.0	108	53.5	24	75.0
BMI group of adolescent								
Not OW	213	88.8	31	63.3				
OW/OB	27	11.2	18	36.7				
Income								
High	94	39.5	16	33.3	99	40.9	12	26.7
Middle	111	46.6	26	54.2	112	46.3	25	55.6
Low	33	13.9	6	12.5	31	12.8	8	17.8
Maternal education								
Tertiary	138	58.2	20	41.7	144	59.8	14	31.1
<Tertiary	99	41.8	28	58.3	97	40.2	31	68.9
SEI								
Class I	59	24.6	5	10.2	60	24.6	4	8.7

For peer review only - http://bmjopen.bmj.com/site/about/guidelines.xhtml

Class II-IV	131	54.6	25	51.0	130	53.3	27	58.7
Class V	50	20.8	19	38.8	54	22.1	15	32.6
City of residence								
White-collar	132	55	23	46.9	137	56.1	18	39.1
Blue-collar	108	45	26	53.1	107	43.9	28	60.9
	M	SD	M	SD	M	SD	M	SD
Age at follow-up (years)	16.02	0.90	16.12	1.04	16.02	0.92	16.18	0.94
Height (m)	1.72	0.09	1.73	0.10	1.73	0.09	1.71	0.09
Birthweight (100g)	35.47	5.63	36.08	5.89	35.32	5.64	37.00	5.68

BMI, Body Mass Index; *CRD*, Cardiovascular risk diet index; *OW/OB*, Overweight or obese; *SEI*, Swedish socioeconomic classification index.

For peer review only

Table 2. Univariate analysis with odds ratios for cardiovascular risk factors and areas under the ROC-curve for SES variables (n = 298).

	Elevated blood pressure		Low HDL		High LDL		Overweight	
	OR	CI	OR	CI	OR	CI	OR	CI
Age at follow-up (Z-years)	1.12	0.83,1.51	1.12	0.79,1.59	0.99	0.63,1.53	1.19	0.87,1.63
Height (Z-m)	1.24	0.91,1.64	1.41	0.85,2.33	1.26	0.85,1.88	0.82	0.59,1.14
Gender								
Girl	1.0		1.0		1.0		1.0	
Boy	1.61	0.86,3.04	2.78	1.09,7.09	1.31	0.57,3.00	1.38	0.73,2.60
Birthweight (Z-100g)	1.11	0.80,1.54	1.75	1.19,2.59	0.72	0.46,1.14	1.37	0.99,1.91
Ethnicity								
Swedish	1.0		1.0		1.0		1.0	
Foreign	0.83	0.27,2.58	1.49	0.53,4.19	0.29	0.04,2.25	1.13	0.41,3.16
Physical activity – times/week								
>2	1.0		1.0		1.0		1.0	
0-2	0.39	0.15,1.05	0.93	0.41,2.26	0.68	0.28,1.67	1.60	0.72,3.55
CRD								
0-1 item	1.0		1.0		1.0		1.0	
2-5 items	1.12	0.49,2.61	2.41	0.95,6.15	1.41	0.63,3.16	2.80	1.06,7.41
BMI group of adolescent								
Not OW	1.0		1.0		1.0			
OW/OB	4.42	2.19,8.95	3.44	1.47,8.07	3.20	1.28,7.98		
Income (ROC)	0.537		0.627		0.545		0.580	
High	1.0		1.0		1.0		1.0	

For peer review only - http://bmjopen.bmj.com/site/about/guidelines.xhtml

Middle	1.35	0.68,2.67	0.88	0.36,2.16	1.08	0.42,2.77	1.90	0.91,3.97
Low	1.11	0.41,3.05	3.94	1.39,11.14	1.73	0.46,6.54	2.21	0.82,5.95
Maternal education (ROC)								
	0.578		0.610		0.564		0.641	
Tertiary	1.0		1.0		1.0		1.0	
<Tertiary	1.87	0.99,3.53	2.39	1.10,5.21	1.67	0.77,3.59	3.22	1.61,6.44
SEI								
(ROC)	0.623		0.540		0.573		0.602	
Class I	1.0		1.0		1.0		1.0	
Class II-IV	2.20	0.80,6.05	1.33	0.48,3.66	1.91	0.60,6.09	3.21	1.08,9.57
Class V	4.37	1.49,12.81	1.45	0.48,4.48	1.41	0.24,8.28	4.28	1.34,13.73
City of residence (ROC)	0.541		0.652		0.511		0.585	
White-collar	1.0		1.0		1.0		1.0	
Blue-collar	1.39	0.76,2.56	1.78	0.74,4.31	1.10	0.51,2.41	2.00	1.06,3.78

BMI, Body Mass Index; *CRD*, Cardiovascular risk diet index; *CI*, 95% confidence interval; *OR*, odds ratio; *OW/OB*, Overweight or obese; *ROC*, area under the receiver operating characteristic curve; *SEI*, Swedish socioeconomic classification index.

For peer review only

For peer review only - http://bmjopen.bmj.com/site/about/guidelines.xhtml

Table 3. Multivariate analysis with odds ratios for cardiovascular risk factors. Adjusted for SES and other explanatory variables (n = 298).

		Elevated blood pressure					
		Model 1		Model 2		Model 3	
		OR	CI	OR	CI	OR	CI
Income	High	1.00		1.00		1.00	
	Middle	0.85	0.39,1.85	0.85	0.38,1.89	0.75	0.32,1.75
	Low	0.60	0.20,1.81	0.69	0.22,2.12	0.60	0.17,1.96
Maternal education	Tertiary	1.00		1.00		1.00	
	<Tertiary	1.29	0.60,2.77	1.15	0.52,2.54	0.88	0.37,2.08
SEI	Class I	1.00		1.00		1.00	
	Class II-IV	2.21	0.77,6.34	2.25	0.78,6.52	2.09	0.70,6.27
	Class V	4.18	1.22,14.41	4.43	1,26,15.58	4.95	1.30,18.89
City of residence	White-collar	1.00		1.00		1.00	
	Blue-collar	1.12	0.58,2.14	1.18	0.61,2.30	1.05	0.52,2.13

		Low HDL (<0.40 mg/dL).					
		Model 1		Model 2		Model 3	
		OR	CI	OR	CI	OR	CI
Income	High	1.00		1.00		1.00	
	Middle	0.98	0.31,3.08	0.81	0.29,2.29	0.82	0.28,2.40
	Low	3.16	0.97,10.35	3.59	0.96,13.38	3.76	0.95,14.99
Maternal education	Tertiary	1.00		1.00		1.00	
	<Tertiary	2.56	0.99,6.62	2.13	0.80,5.71	1.85	0.67,5.14
SEI	Class I	1.00		1.00		1.00	
	Class II-IV	0.88	0.32,2.48	0.79	0.28,2.27	0.73	0.25,2.12
	Class V	0.53	0.14,2.07	0.45	0.11,1.92	0.43	0.10,1.92
City of residence	White-collar	1.00		1.00		1.00	
	Blue-collar	1.41	0.55,3.60	1.20	0.46,3.12	1.09	0.40,2.93

High LDL (>110 mg/dL)

For peer review only

		Model 1		Model 2		Model 3	
		OR	CI	OR	CI	OR	CI
Income	High	1.00		1.00		1.00	
	Middle	0.91	0.32,2.56	0.88	0.31,2.51	0.84	0.29,2.49
	Low	1.48	0.35,6.21	1.47	0.34,6.29	1.41	0.31,6.39
Maternal education	Tertiary	1.00		1.00		1.00	
	<Tertiary	1.86	0.70,4.93	1.81	0.68,4.79	1.58	0.58,4.32
SEI	Class I	1.00		1.00		1.00	
	Class II-IV	1.69	0.49,5.77	1.68	0.49,5.79	1.52	0.43,5.40
	Class V	0.92	0.10,8.56	0.97	0.10,9.06	0.90	0.09,9.54
City of residence	White-collar	1.00		1.00		1.00	
	Blue-collar	0.89	0.37,2.15	0.90	0.37,2.18	0.83	0.33,2.09

		Overweight status			
		Model 1		Model 2	
		OR	CI	OR	CI
Income	High	1.00		1.00	
	Middle	1.16	0.52,2.60	1.12	0.50,2.54
	Low	1.21	0.42,3.54	1.20	0.40,3.56
Maternal education	Tertiary	1.00		1.00	
	<Tertiary	2.63	1.20,5.78	2.39	1.08,5.30
SEI	Class I	1.00		1.00	
	Class II-IV	2.34	0.75,7.28	2.09	0.66,6.60
	Class V	1.90	0.52,6.98	1.63	0.43,6.25
City of residence	White-collar	1.00		1.00	
	Blue-collar	1.39	0.70,2.73	1.39	0.70,2.76

BMI, Body Mass Index; *CI*, 95% confidence interval; *OR*, odds ratio; *SEI*, Swedish socioeconomic classification index.

Model 1 includes all SES variables. Model 2 includes all SES and other variables excluding overweight status that reached the selection criteria in univariate analysis ($p \leq 0.05$ or OR <0.5 or >2.0 for categorical and <0.75 or >1.5 for continuous variables): physical activity for elevated blood pressure; gender, birthweight and cardiovascular risk diet for low HDL; ethnicity for LDL; cardiovascular risk diet for overweight. Model 3 includes all SES, other explanatory variables and overweight status.

For peer review only - http://bmjopen.bmj.com/site/about/guidelines.xhtml

Table 4. Association between maternal education level at birth and child overweight or obesity at four ages.

Time point:	2 years	5 years	8 years	12 years
Cohort approach				
Age: mean (SD)	2.72 (0.28)	5.32 (0.30)	7.86 (0.34)	12.61 (0.87)
Sample size available	1070	1070	1070	1070
Overweight: n (%)	149 (13.9%)	146 (13.6%)	157 (14.7%)	168 (15.7%)
Obese: n (%)	19 (1.8%)	27 (2.5%)	21 (2.0%)	21 (2.0%)
Tertiary v < Tertiary education: overweight*	0.95 (0.67, 1.34)	1.42 (1.00, 2.03)	1.36 (0.96, 1.92)	1.81 (1.28, 2.55)
Tertiary v < Tertiary education: obesity*	0.41 (0.15, 1.08)	3.21 (1.29, 8.02)	1.46 (0.60, 3.56)	3.89 (1.30, 11.63)
Available sample approach				
Age: mean (SD)	2.73 (0.30)	5.34 (0.31)	7.86 (0.38)	12.40 (0.91)
Sample size available	7925	6602	2998	3337
Overweight: n (%)	1187 (15.0%)	1096 (16.6%)	446 (14.9%)	527 (15.8%)
Obese: n (%)	191 (2.4%)	273 (4.1%)	71 (2.4%)	75 (2.2%)
Tertiary v < Tertiary education: overweight*	1.18 (1.03, 1.34)	1.30 (1.13, 1.50)	1.16 (0.94, 1.42)	1.70 (1.39, 2.06)
Tertiary v < Tertiary education: obesity*	1.56 (1.12, 2.17)	1.52 (1.15, 1.99)	2.28 (1.31, 3.95)	2.16 (1.29, 3.61)

*Odds ratio (95% confidence interval)

Note: The cohort approach utilises only participants with all necessary data recorded at all four ages whereas the available sample approach uses any participant with all necessary data recorded on at least one age.

For peer review only

Supplementary table 1: Comparison of original ABIS cohort, invited subsample, participants and non-participants in the cardiovascular risk study.

	Original cohort		Invited to subsample		Participants		Non- participants		p[1]	p[2]	p-[3]
	N=16349		N=578		N=298		N=240				
	Mean/N	SD/%	Mean/N	SD/%	Mean/N	SD/%	Mean/N	SD/%			
Birthweight (g)	3578.6	560.8	3552.6	553.6	3554.0	574.7	3551.1	530.8	0.27	0.46	0.95
Gender									0.11	0.05	0.23
Boys	8473	51.9 %	279	48.5	136	46.1	143	46.1 %			
Girls	7866	48.1 %	296	51.5	159	53.9	137	48.9 %			
Ethnicity									0.09	0.50	0.01
Swedish	14235	89.3 %	492	87.1 %	266	90.5 %	226	83.4 %			
Other	1711	10.7 %	73	12.9 %	28	9.5 %	45	16.6 %			
Father ow/ob[a]									0.19	0.54	0.51
Not ow/ob	5384	54.0 %	254	56.4 %	146	57.3 %	108	57.3 %			
Overweight	4003	40.1 %	164	36.4 %	94	36.9 %	70	35.9 %			
Obese	586	5.9 %	32	7.1 %	15	5.9 %	17	8.7 %			
Mother ow/ob[a]									0.95	0.22	0.01
Not ow/ob	7310	70.0 %	328	69.6 %	191	72.9 %	137	65.6 %			

For peer review only

Overweight	2372	22.7 %	107	22.7 %	59	22.5 %	48	23.0 %			
Obese	759	7.3 %	36	7.6 %	12	4.6 %	24	11.5 %			
Overweight at 2 yrs									0.46	0.75	0.71
Not ow/ob	6890	85.0 %	307	83.7 %	187	84.2 %	120	82.8 %			
Ow/ob	1217	15.0 %	60	16.3 %	35	15.8 %	25	17.2 %			
Overweight at 5 yrs									0.35	<0.01	<0.01
Not ow/ob	5624	83.3 %	287	85.2 %	187	90.3 %	100	76.9 %			
Ow/ob	1125	16.7 %	50	14.8 %	20	9.7 %	30	23.1 %			
Overweight at 8 yrs									0.76	0.51	0.10
Not ow/ob	2566	85.1 %	2566	85.1 %	139	86.9 %	50	78.1 %			
Ow/ob	450	14.9 %	450	14.9 %	21	13.1 %	14	21.9 %			
Overweight at 12 yrs									0.63	0.20	0.08
Not ow/ob	2836	84.2 %	197	85.3 %	155	87.6 %	42	77.8 %			
Ow/ob	534	15.8 %	34	14.7 %	22	12.4 %	12	22,2 %			
Maternal education									< 0.01	< 0.01	<0.01
High	5062	31.8 %	233	41.3 %	160	54.4 %	73	27.0 %			
Low	10882	68.2 %	331	58.7 %	134	45.6 %	197	73.0 %	197	73.0 %	
Income									< 0.01	< 0.01	< 0.01
High	3239	20.0 %	153	26.7 %	114	38.6 %	39	14.0 %			
Middle	9718	60.0 %	301	52.5 %	141	47.8 %	160	57.6 %			
Low	3239	20.0 %	119	20.8 %	40	13.6 %	79	28.4 %			

For peer review only

p-value[1]= Invited to follow-up vs original cohort, T-test or Chi2 as appropriate
p-value[2]= Participants in follow-up vs original cohort, T-test or Chi2 as appropriate
p-value[3]=Participants vs non-participants in follow-up, T-test or Chi2 as appropriate
[a] Parental height and weight self-reported at child age 1 year
T-test or Chi2 as appropriate

For peer review only

Reporting checklist for cohort study.

Based on the STROBE cohort guidelines.

Instructions to authors

Complete this checklist by entering the page numbers from your manuscript where readers will find each of the items listed below.

Your article may not currently address all the items on the checklist. Please modify your text to include the missing information. If you are certain that an item does not apply, please write "n/a" and provide a short explanation.

Upload your completed checklist as an extra file when you submit to a journal.

In your methods section, say that you used the STROBE cohort reporting guidelines, and cite them as:

von Elm E, Altman DG, Egger M, Pocock SJ, Gotzsche PC, Vandenbroucke JP. The Strengthening the Reporting of Observational Studies in Epidemiology (STROBE) Statement: guidelines for reporting observational studies.

		Reporting Item	Page Number
Title	#1a	Indicate the study's design with a commonly used term in the title or the abstract	1
Abstract	#1b	Provide in the abstract an informative and balanced summary of what was done and what was found	2
Background / rationale	#2	Explain the scientific background and rationale for the investigation being reported	4
Objectives	#3	State specific objectives, including any prespecified hypotheses	5
Study design	#4	Present key elements of study design early in the paper	6
Setting	#5	Describe the setting, locations, and relevant dates, including periods of recruitment, exposure, follow-up, and data collection	6-7

For peer review only

Eligibility criteria	#6a	Give the eligibility criteria, and the sources and methods of selection of participants. Describe methods of follow-up.	6-7
	#6b	For matched studies, give matching criteria and number of exposed and unexposed	n/a
Variables	#7	Clearly define all outcomes, exposures, predictors, potential confounders, and effect modifiers. Give diagnostic criteria, if applicable	7-10
Data sources / measurement	#8	For each variable of interest give sources of data and details of methods of assessment (measurement). Describe comparability of assessment methods if there is more than one group. Give information separately for for exposed and unexposed groups if applicable.	7-10
Bias	#9	Describe any efforts to address potential sources of bias	10
Study size	#10	Explain how the study size was arrived at	5
Quantitative variables	#11	Explain how quantitative variables were handled in the analyses. If applicable, describe which groupings were chosen, and why	7-10
Statistical methods	#12a	Describe all statistical methods, including those used to control for confounding	10
	#12b	Describe any methods used to examine subgroups and interactions	N/A
	#12c	Explain how missing data were addressed	10
	#12d	If applicable, explain how loss to follow-up was addressed	6-7
	#12e	Describe any sensitivity analyses	N/A
Participants	#13a	Report numbers of individuals at each stage of study—eg numbers potentially eligible, examined for eligibility, confirmed eligible, included in the study, completing follow-up, and analysed. Give information separately for for exposed and unexposed groups if applicable.	12-13
	#13b	Give reasons for non-participation at each stage	7
	#13c	Consider use of a flow diagram	N/A

For peer review only

Descriptive data	#14a	Give characteristics of study participants (eg demographic, clinical, social) and information on exposures and potential confounders. Give information separately for exposed and unexposed groups if applicable.	Table 1
	#14b	Indicate number of participants with missing data for each variable of interest	Table 1
	#14c	Summarise follow-up time (eg, average and total amount)	6
Outcome data	#15	Report numbers of outcome events or summary measures over time. Give information separately for exposed and unexposed groups if applicable.	12-13
Main results	#16a	Give unadjusted estimates and, if applicable, confounder-adjusted estimates and their precision (eg, 95% confidence interval). Make clear which confounders were adjusted for and why they were included	10-13
	#16b	Report category boundaries when continuous variables were categorized	8-10
	#16c	If relevant, consider translating estimates of relative risk into absolute risk for a meaningful time period	N/A
Other analyses	#17	Report other analyses done—e.g., analyses of subgroups and interactions, and sensitivity analyses	N/A
Key results	#18	Summarise key results with reference to study objectives	14
Limitations	#19	Discuss limitations of the study, taking into account sources of potential bias or imprecision. Discuss both direction and magnitude of any potential bias.	15-16
Interpretation	#20	Give a cautious overall interpretation considering objectives, limitations, multiplicity of analyses, results from similar studies, and other relevant evidence.	17-18
Generalisability	#21	Discuss the generalisability (external validity) of the study results	15-16
Funding	#22	Give the source of funding and the role of the funders for the present study and, if applicable, for the original study on which the present article is based	18

For peer review only

The STROBE checklist is distributed under the terms of the Creative Commons Attribution License CC-BY. This checklist was completed on 22. March 2019 using https://www.goodreports.org/, a tool made by the EQUATOR Network in collaboration with Penelope.ai

For peer review only

BMJ Open

Inequalities in Cardiovascular risks among Swedish adolescents – ABIS a prospective cohort study

Journal:	*BMJ Open*
Manuscript ID	bmjopen-2019-030613.R1
Article Type:	Original research
Date Submitted by the Author:	04-Nov-2019
Complete List of Authors:	Andersson White, Pär; Linköping University, Dept. of Medicine and Health; Linköping University Hospital, Crown Princess Victoria Children´s Hospital<br>Ludvigsson, Johnny; Linkopings universitet, Div of Pediatrics, Dept of Clin Exp Medicin, Medical Faculty<br>Jones, Michael; Macquarie University<br>Faresjo, Tomas; Linköpings universitet Hälsouniversitetet
**Primary Subject Heading**:	Paediatrics
Secondary Subject Heading:	Epidemiology, Cardiovascular medicine, Public health
Keywords:	PUBLIC HEALTH, EPIDEMIOLOGY, Cardiac Epidemiology < CARDIOLOGY, Paediatric endocrinology < DIABETES & ENDOCRINOLOGY, SOCIAL MEDICINE

SCHOLARONE™
Manuscripts

I, the Submitting Author has the right to grant and does grant on behalf of all authors of the Work (as defined in the below author licence), an exclusive licence and/or a non-exclusive licence for contributions from authors who are: i) UK Crown employees; ii) where BMJ has agreed a CC-BY licence shall apply, and/or iii) in accordance with the terms applicable for US Federal Government officers or employees acting as part of their official duties; on a worldwide, perpetual, irrevocable, royalty-free basis to BMJ Publishing Group Ltd ("BMJ") its licensees and where the relevant Journal is co-owned by BMJ to the co-owners of the Journal, to publish the Work in this journal and any other BMJ products and to exploit all rights, as set out in our licence.

The Submitting Author accepts and understands that any supply made under these terms is made by BMJ to the Submitting Author unless you are acting as an employee on behalf of your employer or a postgraduate student of an affiliated institution which is paying any applicable article publishing charge ("APC") for Open Access articles. Where the Submitting Author wishes to make the Work available on an Open Access basis (and intends to pay the relevant APC), the terms of reuse of such Open Access shall be governed by a Creative Commons licence – details of these licences and which Creative Commons licence will apply to this Work are set out in our licence referred to above.

Other than as permitted in any relevant BMJ Author's Self Archiving Policies, I confirm this Work has not been accepted for publication elsewhere, is not being considered for publication elsewhere and does not duplicate material already published. I confirm all authors consent to publication of this Work and authorise the granting of this licence.

For peer review only

Inequalities in Cardiovascular risks among Swedish adolescents – ABIS a prospective cohort study

Pär Andersson White[1, 2], Johnny Ludvigsson[2, 3], Michael P. Jones[3], Tomas Faresjö[1]

[1]Dept. of Medicine and Health, Div. of Community Medicine, Linköping University, Sweden.

[2]Crown Princess Victoria Children´s Hospital and Div. of Pediatrics, Dept. of Clinical and Experimental Medicine, Linköping University, Sweden.

[3]Psychology Department, Macquarie University, North Ryde, NSW, 2109, Australia.

Corresponding author: Pär Andersson White, C/O T Faresjo, Dept. of Medicine and Health, Div. of Community Medicine, Linköping University, SE-581 83 Linköping Sweden, +4610-1030000, par.andersson.white@liu.se

Keywords: Socioeconomic status, Inequalities, Adolescent Overweight, Childhood overweight, Childhood obesity, Blood Pressure, Dyslipidemia, High-Density Lipoproteins, Low-Density Lipoproteins, ABIS

For peer review only

Abstract

OBJECTIVES: To investigate if Socioeconomic Status (SES) is related to cardiovascular risk factors among Swedish adolescents. Identify the most important SES variable for the development of each cardiovascular risk factor. Investigate at what age SES inequality in overweight and obesity occurs.

DESIGN: Longitudinal follow-up of a prospective birth cohort.

SETTING: All Babies in Southeast Sweden (ABIS) study includes data from children born between October 1997 and October 1999 in five counties of south east Sweden.

PARTICIPANTS: A regional ABIS-study subsample from three major cities of the region n=298 adolescents aged 16-18 years, and prospective data from the whole ABIS cohort for overweight and obesity status at the ages 2, 5, 8 and 12 years (n= 2998-7925).

OUTCOME MEASURES: Blood pressure above the hypertension limit, overweight/obesity according to the International Obesity Task Force (IOTF) definition, low high-density lipoproteins (HDL) or borderline-high low-density lipoproteins (LDL) according to National Cholesterol Education Program (NCEP) expert panel on cholesterol levels in children.

RESULTS: For three out of four cardiovascular risk outcomes (elevated blood pressure, low HDL and overweight/obesity) there were increased risk in one or more of the low SES groups ($p < 0.05$). The best predictor was parental occupational class (SEI) for elevated blood pressure (Area under the ROC curve 0.623), maternal educational level for overweight (Area under the ROC curve 0.641) and blue-collar city of residence for low high-density lipoprotein (Area under the ROC curve 0.641). SES related differences in overweight/obesity were found at age 2, 5 and 12 and for obesity at age 2, 5, 8, and 12 years (all $p<0.05$).

For peer review only - http://bmjopen.bmj.com/site/about/guidelines.xhtml

For peer review only

CONCLUSIONS: Even in a welfare state like Sweden, SES inequalities in cardiovascular risks are evident already in childhood and adolescence. Intervention programs to reduce cardiovascular risk based on social inequality should start early in life.

Strengths and limitations of this study:

- Data derives from a large prospective cohort representative for the general population.
- A standardized procedure for collection of clinical data. The same nurse has done all blood pressure measurements, taken all blood samples and collected data on weight and height.
- The Swedish registers give us valid and reliable data on household income.
- The limited sample size of the follow–up group gives some restraints.

For peer review only

Introduction

Cardiovascular disease in adults has been shown to be one of the disease groups most correlated with socioeconomic inequalities in the world [1], and this has also been evident in Swedish adults [2]. Longitudinal studies have shown that risk factors for cardiovascular disease develop in childhood and that early signs of cardiovascular disease risks are visible in early adulthood [3-5]. The risk factors associated with cardiovascular disease include blood pressure, dyslipidemia and overweight. Of these risk factors, overweight and obesity are the ones that progress most consistently from childhood to adulthood. Adolescent overweight has been shown to be a stronger predictor of cardiovascular mortality in adulthood than adult overweight [6].

Recent studies report lower scores than ideal in a range of cardiovascular health factors (blood pressure, cholesterol and glucose level below the American heart association's cut-offs) in European adolescents with low maternal education [7]. In Korea high income was protective against cardiovascular disease risk factors in and in Finland low socioeconomic status measured by occupation of the father was associated with higher low-density lipoproteins (LDL) levels in boys and higher systolic blood pressure and higher percentage of smokers [8 9]. However, longitudinal data on the relationships between childhood socioeconomic status (SES) and adolescent's cardiovascular health is still scarce.

An increase in physical activity has been shown to reduce the risk of being overweight and obese as a youth in Sweden and other European countries [10]. Studying the effect of diet on development of overweight and obesity in children and adolescents has yielded no clear conclusions on whether any specific foods are important for the development of obesity [11]. However, there is some evidence for intake of certain foods and development of adult

For peer review only - http://bmjopen.bmj.com/site/about/guidelines.xhtml

cardiovascular disease. The INTERHEART study found a positive association between intake of meat, salty foods, fried foods and development of cardiovascular disease and a negative association between intake of fruits and vegetables and cardiovascular disease risk [12].

Although Sweden has dropped in the Organization for Economic Co-operation and Development (OECD) list of economic equality from 1st place in the 1980ies to 9th in 2016 [13], Sweden still has a comprehensive public health system with a number of interventions aimed at reducing social inequalities. These include universal and public financed healthcare equally accessible for all. This includes free antenatal consulting and check-ups at Child Health Care Centers which covers advice on nutrition and health from professional nurses for all children at regular intervals up to school age. Other interventions include school-health check-ups and mandatory physical activity in schools and free school lunches for all children. Other support for families with children include paid maternity leave for 480 days and heavily subsidized daycare. Public health interventions include encouragement of attendance at antenatal and postnatal clinics, support of breastfeeding, as well as taxes deliberately aimed at reducing tobacco and alcohol consumption[14 15].

Aims

The aims of this study were to determine whether social inequalities are related to cardiovascular risk factors including blood pressure, lipids and overweight in Swedish adolescents, and if so, to identify which socioeconomic factors that were most important for the development of each risk factor, and at what ages socioeconomic differences in overweight and obesity occur using longitudinal data from follow-ups in the ABIS prospective study.

For peer review only - http://bmjopen.bmj.com/site/about/guidelines.xhtml

Material and Methods

Study population

The ABIS study (All Babies In southeast Sweden) is a prospective population-based cohort study, initially including 17 055 children born between October 1st, 1997 and October 1st 1999 in Southeast of Sweden in the counties of Östergötland, Jönköping, Kronoberg, Kalmar, and Blekinge. The ABIS region is inhabited by approx. 1.25 million out of Sweden's 10 million inhabitants[16]. The region incorporates three of the biggest cities of the country, Linköping (5th), Norrköping (9th) and Jönköping (10th), but also large rural areas, both farmland and forest areas. In terms of income inequality, the counties of the south eastern Sweden are placed close to the average of the country between the most unequal areas (the capital Stockholm) and the northern areas with the lowest income inequality[17]. This cohort of children has been followed from birth and are still being followed with respect to biological samples, questionnaires and register data. The initial aim of the ABIS-study was to analyse the development of immune-mediated diseases including autoimmune diseases, type 1 and type 2 diabetes as well as cardiovascular disease, and the effect of different environmental and genetic factors.

We undertook a follow-up of a regional subsample of ABIS-children and also made a questionnaire-based longitudinal analysis for the whole cohort. Inclusion criteria for the of the three major cities of Östergötland County. Out of n=578 children invited n=298 (51.6%) participated. The main reasons for non-participation among the invited sample were practical i.e. difficulties to arrange health check-ups for our research nurse at some schools that lacked school-nurse facilities. The study participants filled out a questionnaire about their lifestyle, diet and physical activity. A specially trained research nurse undertook all

For peer review only - http://bmjopen.bmj.com/site/about/guidelines.xhtml

data collection including blood pressure measurement, questionnaire and collection of blood samples and measurement of anthropometric data.

Blood Pressure

Systolic and diastolic blood pressure was measured manually by the research nurse at the participant's school nursing offices. Study participants rested on a bed at least 5 minutes before measurement, two consecutive measurements of blood pressure were then taken in the sitting position and the mean value was calculated. Blood pressures were grouped as hyper- or normotensive according to the guidelines described in the fourth report on the diagnosis, evaluation, and treatment of high blood pressure in children and adolescents [18]. These guidelines define hypertension as an average systolic and/or diastolic blood pressure ≥95th percentile for gender, age and height percentile according to the Center of Disease Control and Preventions growth charts on three or more occasions [19].

Overweight/obesity

The participating adolescents' height (cm) and weight (kg) were measured by the research nurse. Data on weight and height were missing for 8 participants. Body mass index (BMI) was calculated as BMI = weight(kg)/height(m)^2 and dichotomized as overweight/obese or not, using the International obesity task force latest age and gender specific cut-offs defined by Cole et al [20 21]. The same procedure was then used for parental-reported height and weight of the child available from previous ABIS follow-ups at age 2, 5, 8 and 12 years.

Dyslipidemia

Blood samples for blood lipid analysis were collected from 242 (81.2 %) individuals and analyzed for plasma levels of LDL and HDL at the Department of clinical chemistry, Linköping

For peer review only - http://bmjopen.bmj.com/site/about/guidelines.xhtml

University Hospital, according to accredited procedures. One sample was missing data on LDL after analysis. Test results were converted from SI units to mg/dL. Results were then grouped as normal or low HDL cholesterol in accordance with the cut-off for low HDL cholesterol (<40 mg/dL) set by the Expert panel on integrated guidelines for cardiovascular health and risk reduction in children, which is based on the 10th percentile found by the National cholesterol educational program (NCEP) [22 23]. For LDL only 8 (3.3%) out of 241 had a level above the limit of high LDL (the 95th percentile in NCEP). As this number was insufficient for statistical analyses the cut-off of borderline high LDL (>110 mg/dL, the 75th percentile in NCEP) was chosen as indicator of increased LDL.

Socioeconomic variables

A five grade Socioeconomic Class (SEI) variable was constructed based on parents occupation and the occupational groups used by Statistics Sweden in their Swedish socioeconomic classification index[24]. The definitions for each class was; Class I Professionals (SEI 54-60), Class II Intermediate non-manual employees (SEI 44-46), Class III Assistant non-manual employees (SEI 33-36), Class IV Skilled workers (SEI 21-22) and Class V Unskilled workers (SEI 11-12). Occupations in SEI 76-87 which includes non-professional self-employees including farmers were given a Class I-V based on their educational attainment. The adolescents were classified according to the highest occupational class of either of their parents. Mother's educational level was derived from the ABIS birth questionnaire and grouped as primary/secondary education or post-secondary education/university. Family disposable income was derived from Statistics Sweden for the year 2012 and grouped into three groups; low income representing the bottom 20 % of disposable incomes in the

For peer review only - http://bmjopen.bmj.com/site/about/guidelines.xhtml

original cohort (available for n=16196), middle income representing the middle 20 to 80 percentile of income and top income representing the top 20 percent of income.

The three included cities in this study represent different social history and different socioeconomic environments[25]. The participants from the city of Motala and Norrköping, are towns with a mainly industrial history, and were merged in the analysis and labelled as blue-collar cities. The city of Linköping has a history as a political and ecclesiastical administrative centre of the county, was labelled as white-collar city. Today this city has a workforce of mainly civil servants including high tech industries and one of Sweden’s major universities. Previous studies of these cities has exhibit marked public health differences, including higher mortality in cardiovascular disease among adults in the blue-collar cities and difference in life-expectancy[26 27]

Covariates

Birthweight was reported by the parents in the original ABIS birth questionnaire. Age was calculated using the date of answering the questionnaire and the participants birth date. Height was measured at follow-up by a research nurse. Ethnicity was based on the question “Are you (the mother) born in Sweden” and “Is the father born in Sweden”. The adolescents were then grouped as having foreign ethnicity if one of the parents was born outside of Sweden. A cardiovascular risk diet index (CRD) was constructed based on the dietary components most associated with myocardial infarction in the INTERHEART study [12]. These factors were: intake of salty foods one time per day or more, intake of deep-fried foods three times or more per week, intake of meat two or more times per day, intake of fruit less than once per day and intake of vegetables less than once per day. This score which has a

For peer review only - http://bmjopen.bmj.com/site/about/guidelines.xhtml

range of 0 to 5 was then dichotomized into two groups Low CRD (0-1 items n=101) and High CRD (2 to 5 items n=138). Degree of physical activity was measured by the question “How many times per week do you do physical activity at least 30 min that is strenuous enough to make you sweat”. The answers were then grouped into two groups; 0-2 times a week, >2 times a week.

Patient and Public Involvement

Patients and public were not involved in the design of this study. The results will be disseminated to the study participants through the ABIS study homepage https://www.abis-studien.se/.

Statistical analysis

Data were analyzed using bivariate logistic regression for each cardiovascular risk factor. As analysis of missing values showed significant differences due to socioeconomic status, adolescents with low maternal educational level had a significantly higher number of missing values in questionnaire data, multiple imputation was used for missing values to reduce this potential bias. Multiple imputation was employed as implemented in the SPSS statistics program. A Markov Chain Monte Carlo was employed with predictive models selected according to the measurement scale of each dependent variable specified in five imputation samples. A univariate logistic regression was then analyzed for each independent variable (SES variables and covariates) and Odds Ratios (OR) with 95% confidence interval for each dependent variable (elevated blood pressure, low-HDL, high-LDL and overweight/obesity at last follow-up) were calculated. For further multivariate

For peer review only - http://bmjopen.bmj.com/site/about/guidelines.xhtml

analysis all four SES variables (maternal education, income, SEI, white/blue collar city) were included on a priori basis while other independent variables were chosen based on reaching statistical significance $p \leq 0.05$ or an effect size of OR <0.5 or >2.0 for categorical variables. For continuous variables; height, age and birthweight, was standardized and the OR for 1 standard deviation increase of <0.75 or >1.5 was used as selection criteria. This approach was adopted due to limited sample size and, in this context, effect size being as important from a public health perspective as statistical significance.

The degree of discrimination afforded by each socioeconomic variable was quantified by the area under the receiver operating characteristic (ROC) curve defined by taking the logistic model predicted probability of adverse health state as the test measure and the actual state as an imperfect reference standard. Values >0.5 indicate some discrimination with values close to 1.0 indicating excellent discrimination.

A hierarchical approach was adopted in which SES variables were first adjusted for other SES variables, then for other independent variables reaching the selection criteria and finally in three of the four outcomes (elevated blood pressure, low LDL and high LDL) for adolescent's overweight status.

Bivariate logistic regression was also used in the final analysis on the relationship between overweight/obesity and maternal educational level at four different time points. The first analysis used a cohort approach and included only subjects with complete data from all follow-ups (n = 1070). The second analysis used an available sample approach and included all subjects that had data for any specific age (n=2995-7925).

Statistical analysis was performed using Statistical Package for the Social Sciences v 23 (SPSS, Chicago, IL, USA).

For peer review only - http://bmjopen.bmj.com/site/about/guidelines.xhtml

Ethical considerations

The ABIS-study has been approved by the Research Ethics committees of the Faculty of Health Science at Linköping University and Lund university. Dnr 03-092, Dnr 99-227 and Dnr 99-321. All participants in the follow-up gave their informed consent as well as their caregivers consent for those below 18 years of age.

Results

The participants in our follow-up had a mean age of 16.05 (SD 0.9) years (n=136 males, n=162 females). They were distributed between the cities of Linköping n=160 (54%), Norrköping n=91 (30%) and Motala n=47 (16%). 24 (9.7 %) out of 248 were categorized as having foreign ethnicity. The invited subsample was similar to the original ABIS cohort in respect to a number of variables (birthweight, ethnicity, parental overweight and obesity, child overweight at age 2, 8 and 12 years). However, comparison of this ABIS follow-up with the original ABIS cohort showed a higher proportions of mothers with high education and families with high income, a lower number with overweight at 5 years. There was also a slight difference (p=0.052) in gender composition with boys being underrepresented in the subsample. Non-participation analysis also showed underrepresentation of children with foreign ethnicity, low maternal education and low household income (see supplementary table 1).

The total number of participants that were classified as having elevated blood pressure was 49/290 (16.9 %), see table 1. This was based on 34/290 participants (11.7%) with systolic blood pressures above the limit for hypertension and 17/290 (5.9 %) with diastolic blood pressure above the limit. Two individuals were above both systolic and diastolic hypertension limits. An HDL level below 40 mg/dL was found in 30 (10.1 %) out of 242

For peer review only - http://bmjopen.bmj.com/site/about/guidelines.xhtml

samples. For LDL 25/241 (10.4 %) were above the cut-off for borderline-high value (110 mg/dL).

Elevated blood pressure

All SES variables were associated with increased odds ratios (OR) for elevated blood pressure in the lowest SES groups compared with the highest, see table 2. Out of the four SES variables, parental occupation class variable SEI had the highest area under the ROC curve, 0.623 compared to 0.537-0.578 for the other SES variables. The lowest social class had an OR of 4.37 (95% confidence interval 1.49, 12.81) compared to the highest class.

After adjustments for all other SES variables, physical activity and overweight status the lowest SEI class had an OR for elevated blood pressure of 4.95 (95% confidence interval 1.30, 18.89), see table 3.

Dyslipidemia

All indicators of low SES showed an increased odd ratio of having low HDL. Out of the four variables, Blue-collar city was associated with the highest area under the ROC curve, 0.652 compared to 0.540-0.627 for the other SES variables. Blue-collar city was associated with an OR of 1.78 (95% confidence interval 0.74, 4.31) of low HDL. Low maternal education and low income were both associated with significantly increased risks of low HDL, low maternal education with an OR of 2.39 (95% confidence interval 1.10, 5.21) and low income with an OR of 3.94 (95% confidence interval 1.39, 11.14) for having low HDL.

After adjustment for all other SES variables, gender, CRD and overweight the OR for Blue-collar city was reduced to 1.09 (95% confidence interval 0.40, 2.93).

For peer review only - http://bmjopen.bmj.com/site/about/guidelines.xhtml

The difference between low and high SES groups was small, and no SES variable reached an OR above 2.0, and no variable reached statistical significance $p \leq 0.05$. The area under the ROC curve ranged from 0.573 for SEI to 0.511.

Overweight/obesity

In the regional subsample 46/290 (15.8 %) were overweight or obese, with 7 (2.4 %) obese. All four measures of low SES had an increased odds ratio of being overweight or obese. The best discriminator measured as area under the ROC curve for a single SES variable was 0.641 for maternal education. The only other variable that met the criteria for being included in the multivariate analysis was diet according to CRD.

After adjustment for all other SES variables and CRD maternal education had an odds ratio of 2.39 (95% confidence interval 1.08, 5.30) for being overweight.

Overweight and obesity inequality during childhood

Overweight and obesity differences related to maternal education were measured longitudinally at four different time-points. In the analysis on subjects with complete data (cohort approach) the children to low educated mothers´ had significantly increased odds of being overweight/obese at age 12, but not at age 2, 5 and 8. The analysis based on all available data (available sample approach) showed significant differences at age 2, 5 and 12 but not at age 8. For obesity there were significant differences at age 5 and 12 in the cohort approach, while the available sample approach showed significant differences at all ages, 2, 5, 8 and 12 years, see table 4.

For peer review only - http://bmjopen.bmj.com/site/about/guidelines.xhtml

Discussion

There was significantly increased risk of three out of four cardiovascular risk factors due to low SES in Swedish adolescents. Parental occupational class according to SEI was the most important factor for risk of elevated blood pressure. Although overweight was important for elevated blood pressure the independent importance of parental occupational class was further strengthened as it remained after adjustment for all other SES factors, physical activity and overweight. For other outcomes we found maternal education to be the best discriminator for overweight, and white/blue collar city for low HDL (<40 mg/dL).

In the Whitehall studies, occupational class and blood pressure were found to be closely associated in adults [28]. One finding of the Whitehall II study is that workers with low occupational grade have increased blood pressure during the working day, but no difference in resting blood pressure [29]. As blood pressure in this study was measured while the adolescents' were attending school it is possible that it represents the same kind of increase in response to school tasks in low SES adolescents as that seen during the work day in adult workers.

The impact of maternal education on child overweight has been shown in large multi cohort studies [30]. In addition to being a marker for socioeconomic status like occupation and income the impact of maternal education could involve differences in knowledge. There is evidence that adolescents with mothers with good nutritional knowledge have a healthier diet [31]. High maternal education has also been associated with a higher degree of breakfast eating in adolescents [32]. Further, low parental education has been associated with increased access to sugar sweetened drinks and low vegetable intake [33]. These factors could be part of

For peer review only - http://bmjopen.bmj.com/site/about/guidelines.xhtml

the explanation of why maternal educational level is more important than other SES factors for overweight status in adolescents.

Blue-collar city was the best discriminator of low HDL status. After adjustment for other SES variables the odds ratio for blue-collar cities was reduced to OR 1.20 (0.46, 3.12), adjusting for diet and overweight further decreased the OR to 1.09 (0.40, 2.93) indicating that most of the risk associated with Blue-collar residence is due to differences in SES, diet and overweight. Thus our results are in line with previous studies [25].

The main limitation of this study is the limited number of participants of the follow-up part (n=298). Even though the sample size of study is somewhat restrained we managed to capture socioeconomic related significant differences in cardiovascular risks. Our sample is quite representative concerning overweight status compared to the previous larger samples in ABIS and to other Swedish studies [34]. Although the number of low income families were similar in our invited subsample compared to the original cohort there was an overrepresentation of high maternal education and high income families, this most likely reflects the fact that the subsample is from a more urban population than the rest of the ABIS region and must be taken into consideration when interpreting the result. Among the invited, the number of adolescents with high maternal education at birth and high income families were higher among the participants of the study than in the non-participant group the same was true for high income families. This is a common finding in longitudinal studies, but as previous studies have shown, the effect of this underrepresentation in low SES groups, if anything, is to underestimate inequality slightly in later follow-ups and have little effect on the qualitative conclusion on inequality [35]. The number of adolescents with elevated blood pressure was quite high (16.9%) and as blood pressure was measured only at

For peer review only - http://bmjopen.bmj.com/site/about/guidelines.xhtml

one visit to school by the research nurse some of the adolescents might have had White-coat rather than true hypertension [18]. However, other studies have shown similarly high values in other populations [36]. A recent study in a Swedish cohort also found higher average systolic blood pressure in the age group 14-16 years than was found in the fourth report on the diagnosis, evaluation, and treatment of high blood pressure in children and adolescents [37]. We could not rule out that White-coat hypertension might be related to socioeconomic factors but have no evidence of that.

The fact that the longitudinal data was based on parental-reported height and weight might affect the accuracy of our results. A German study found that parental reported data tends to overestimate the prevalence of overweight in young children and underestimate it in older children, however the same study found the impact of socioeconomic status on this error to be small.[38] Thus, although the exact prevalence of overweight status in our study might differ slightly from the true prevalence the relative difference between SES groups should not.

Different socioeconomic variables can have different effects on health. It is a strength that we investigated not one but four dimensions of socioeconomic status inequality. These dimensions seem to be of varying importance for cardiovascular risk outcomes. Thus our study is an example of the many layers of the social determinants of health that give rise to an individual's risk of disease [39]. Although income, maternal education and occupational status are correlated, it is not surprising that the strongest discriminators for cardiovascular risk factors are occupational class, blue collar city and educational level rather than income in this Swedish cohort, as income inequality still is low compared to most other countries [40].

For peer review only

However, our results show that socioeconomic differences in overweight and obesity could be found already in early childhood also in Sweden.

The implication of adolescent SES differences on adult life cardiovascular health inequalities could be important. Longitudinal data from Finland have shown that cardiovascular risk factors such as elevated blood pressure, serum lipids and obesity tracks to adulthood, increasing the risk of adult abnormalities [41]. Data from the same cohort also shows that adolescent blood pressure and LDL cholesterol are risk factors of adult carotid intima media thickening independent of adult risk factors [42]. A general awareness in primary care is important of the potential risk of elevated blood pressure and other cardiovascular risks in low SES adolescents.

Sweden has for a long time been considered one of the more egalitarian countries in the world and has often been used for comparison in studies on health inequalities as a good example [43 44]. Yet during the last two decades this has started to change with economic inequality increasing faster than in other OECD countries and Sweden has gone from a top position in equality to now being the 9th country in income equality out of the 36 countries in OECD [13]. Previous studies have found that for health problems with higher prevalence in low SES groups, like cardiovascular disease in adults, both the over-all prevalence and absolute inequality between high and low SES groups increases with larger economic inequality [1 45]. Our finding that cardiovascular risks in children and adolescents have a SES gradient also in Sweden could have serious public health implication for the future, as the rapid rise of economic inequality might increase absolute inequalities and prevalence of cardiovascular risk factors in children and adolescents. Concerns for health inequalities have

For peer review only - http://bmjopen.bmj.com/site/about/guidelines.xhtml

increased in recent years spurring the Swedish government to start a Commission for health equality with the aim of finding ways to decrease these inequalities [46].

Conclusion

Despite an extensive welfare system and public health interventions in Sweden, cardiovascular health inequality is evident among Swedish youths and children. Preventive health services and clinicians must be aware of the increased risk of cardiovascular risk factors in low SES children and adolescents and improve prevention programs in this group. Policies aimed at decreasing the evident inequalities in cardiovascular disease in adults should start early and include interventions already in childhood and adolescence.

For peer review only

For peer review only - http://bmjopen.bmj.com/site/about/guidelines.xhtml

Funding

The ABIS-study has been supported by Swedish Research Council (K2005-72X-11242-11A and K2008-69X-20826-01-4) and the Swedish Child Diabetes Foundation (Barndiabetesfonden), JDRF Wallenberg Foundation (K 98-99D-12813-01A), Medical Research Council of Southeast Sweden (FORSS) and the Swedish Council for Working Life and Social Research (FAS2004–1775) and Östgöta Brandstodsbolag. The Research and PhD studies Committee (FUN), Linköping University, Sweden (LiU-).

Competing interests

None declared.

Data sharing statement:

Data from the ABIS registry, including study protocols, report forms and consents may be obtained through requests to the PI and project coordinator Senior professor Johnny Ludvigsson email: Johnny.Ludvigsson@liu.se

Author Contributions

TF, JL and PAW were responsible for the study concept and design. JL is the principal investigator for ABIS. PAW did the statistical analysis with advice from MJ. All authors were responsible for analysis and interpretation of data. PAW drafted the manuscript and TF, JL and MJ undertook critical revision of the manuscript. PAW has full access to all of the data in the study, takes responsibility for the integrity of the data and accuracy of the data analysis and had the final responsibility to submit for publication.

For peer review only

References

1. Wilkinson RG, Pickett KE. Income inequality and socioeconomic gradients in mortality. *Am J Public Health* 2008;98(4):699-704. doi: 10.2105/AJPH.2007.109637
2. Toch-Marquardt M, Menvielle G, Eikemo TA, et al. Occupational class inequalities in all-cause and cause-specific mortality among middle-aged men in 14 European populations during the early 2000s. *PLoS One* 2014;9(9):e108072. doi: 10.1371/journal.pone.0108072
3. Berenson GS. Childhood risk factors predict adult risk associated with subclinical cardiovascular disease. The Bogalusa Heart Study. *Am J Cardiol* 2002;90(10C):3L-7L.
4. Mahoney LT, Burns TL, Stanford W, et al. Coronary risk factors measured in childhood and young adult life are associated with coronary artery calcification in young adults: the Muscatine Study. *Journal of the American College of Cardiology* 1996;27(2):277-84. [published Online First: 1996/02/01]
5. Puolakka E, Pahkala K, Laitinen TT, et al. Childhood Socioeconomic Status and Arterial Stiffness in Adulthood: The Cardiovascular Risk in Young Finns Study. *Hypertension* 2017;70(4):729-35. doi: 10.1161/HYPERTENSIONAHA.117.09718
6. Must A, Jacques PF, Dallal GE, et al. Long-term morbidity and mortality of overweight adolescents. A follow-up of the Harvard Growth Study of 1922 to 1935. *N Engl J Med* 1992;327(19):1350-5. doi: 10.1056/NEJM199211053271904
7. Henriksson P, Henriksson H, Labayen I, et al. Correlates of ideal cardiovascular health in European adolescents: The HELENA study. *Nutrition, metabolism, and cardiovascular diseases : NMCD* 2018;28(2):187-94. doi: 10.1016/j.numecd.2017.10.018
8. Leino M, Porkka KV, Raitakari OT, et al. Influence of parental occupation on coronary heart disease risk factors in children. The Cardiovascular Risk in Young Finns Study. *Int J Epidemiol* 1996;25(6):1189-95.
9. Seo YG, Choi MK, Kang JH, et al. Cardiovascular disease risk factor clustering in children and adolescents: a prospective cohort study. *Archives of disease in childhood* 2018;103(10):968-73. doi: 10.1136/archdischild-2017-313226 [published Online First: 2018/04/14]
10. Janssen I, Katzmarzyk PT, Boyce WF, et al. Comparison of overweight and obesity prevalence in school-aged youth from 34 countries and their relationships with physical activity and dietary patterns. *Obes Rev* 2005;6(2):123-32. doi: 10.1111/j.1467-789X.2005.00176.x
11. Pate RR, O'Neill JR, Liese AD, et al. Factors associated with development of excessive fatness in children and adolescents: a review of prospective studies. *Obes Rev* 2013;14(8):645-58. doi: 10.1111/obr.12035
12. Iqbal R, Anand S, Ounpuu S, et al. Dietary patterns and the risk of acute myocardial infarction in 52 countries: results of the INTERHEART study. *Circulation* 2008;118(19):1929-37. doi: 10.1161/circulationaha.107.738716 [published Online First: 2008/10/22]
13. OECD. Key indicators on the distribution of household disposable income and poverty, 2007, 2015 and 2016 or most recent year. http://www.oecd.org/social/soc/IDD-Key-Indicators.xlsx, 2018.
14. Wettergren B, Blennow M, Hjern A, et al. Child Health Systems in Sweden. *The Journal of pediatrics* 2016;177s:S187-s202. doi: 10.1016/j.jpeds.2016.04.055 [published Online First: 2016/09/27]
15. Försäkringskassan. When the child is born. [Available from: https://www.forsakringskassan.se/privatpers/foralder/nar_barnet_ar_fott/ accessed 4 november 2019.
16. Statistics Sweden. Population in the country, counties and municipalities on 31 March, 2019 and population change in January–March 2019. [Available from: https://www.scb.se/en/finding-statistics/statistics-by-subject-area/population/population-composition/population-statistics/pong/tables-and-graphs/quarterly-population-statistics--municipalities-counties-and-the-whole-country/quarter-1-2019/ accessed 4 november 2019.

For peer review only - http://bmjopen.bmj.com/site/about/guidelines.xhtml

17. Statistics Sweden. Income and tax statistics. [Available from: https://www.scb.se/en/finding-statistics/statistics-by-subject-area/household-finances/income-and-income-distribution/income-and-tax-statistics/ accessed 4 november 2019.
18. National High Blood Pressure Education Program Working Group on High Blood Pressure in C, Adolescents. The fourth report on the diagnosis, evaluation, and treatment of high blood pressure in children and adolescents. *Pediatrics* 2004;114(2 Suppl 4th Report):555-76.
19. Gibbs BG, Forste R. Socioeconomic status, infant feeding practices and early childhood obesity. *Pediatr Obes* 2014;9(2):135-46. doi: 10.1111/j.2047-6310.2013.00155.x
20. Cole TJ, Bellizzi MC, Flegal KM, et al. Establishing a standard definition for child overweight and obesity worldwide: international survey. *BMJ (Clinical research ed)* 2000;320(7244):1240-3.
21. Cole TJ, Lobstein T. Extended international (IOTF) body mass index cut-offs for thinness, overweight and obesity. *Pediatr Obes* 2012;7(4):284-94. doi: 10.1111/j.2047-6310.2012.00064.x
22. National Cholesterol Education Program (NCEP): highlights of the report of the Expert Panel on Blood Cholesterol Levels in Children and Adolescents. *Pediatrics* 1992;89(3):495-501.
23. Expert Panel on Integrated Guidelines for Cardiovascular H, Risk Reduction in C, Adolescents, et al. Expert panel on integrated guidelines for cardiovascular health and risk reduction in children and adolescents: summary report. *Pediatrics* 2011;128 Suppl 5:S213-56. doi: 10.1542/peds.2009-2107C
24. Statistics Sweden. Socioekonomisk indelning [SEI], MIS 1982:4 SEI. [Available from: https://www.scb.se/contentassets/22544e89c6f34ce7ac2e6fefbda407ef/mis1982_4_sei.pdf/ accessed 4 november 2019.
25. Faresjo T, Rahmqvist M. Educational level is a crucial factor for good perceived health in the local community. *Scand J Public Health* 2010;38(6):605-10. doi: 10.1177/1403494810374676
26. Wennerholm C, Grip B, Johansson A, et al. Cardiovascular disease occurrence in two close but different social environments. *Int J Health Geogr* 2011;10:5. doi: 10.1186/1476-072X-10-5
27. Karlen J, Faresjo T, Ludvigsson J. Could the social environment trigger the induction of diabetes related autoantibodies in young children? *Scand J Public Health* 2012;40(2):177-82. doi: 10.1177/1403494811435491 [published Online First: 2012/02/09]
28. Ferrie JE, Shipley MJ, Davey Smith G, et al. Change in health inequalities among British civil servants: the Whitehall II study. *Journal of epidemiology and community health* 2002;56(12):922-6.
29. Steptoe A, Kunz-Ebrecht S, Owen N, et al. Socioeconomic status and stress-related biological responses over the working day. *Psychosomatic medicine* 2003;65(3):461-70. [published Online First: 2003/05/24]
30. Ruiz M, Goldblatt P, Morrison J, et al. Impact of Low Maternal Education on Early Childhood Overweight and Obesity in Europe. *Paediatr Perinat Epidemiol* 2016;30(3):274-84. doi: 10.1111/ppe.12285
31. Tabbakh T, Freeland-Graves JH. The home environment: A mediator of nutrition knowledge and diet quality in adolescents. *Appetite* 2016;105:46-52. doi: 10.1016/j.appet.2016.05.002 [published Online First: 2016/05/14]
32. Gebremariam MK, Henjum S, Hurum E, et al. Mediators of the association between parental education and breakfast consumption among adolescents : the ESSENS study. *BMC pediatrics* 2017;17(1):61. doi: 10.1186/s12887-017-0811-2 [published Online First: 2017/02/24]
33. Gebremariam MK, Lien N, Torheim LE, et al. Perceived rules and accessibility: measurement and mediating role in the association between parental education and vegetable and soft drink intake. *Nutrition journal* 2016;15(1):76. doi: 10.1186/s12937-016-0196-3 [published Online First: 2016/08/18]
34. Angbratt M, Ekberg J, Walter L, et al. Prediction of obesity from infancy to adolescence. *Acta Paediatr* 2011;100(9):1249-52. doi: 10.1111/j.1651-2227.2011.02326.x

For peer review only - http://bmjopen.bmj.com/site/about/guidelines.xhtml

35. Howe LD, Tilling K, Galobardes B, et al. Loss to follow-up in cohort studies: bias in estimates of socioeconomic inequalities. *Epidemiology* 2013;24(1):1-9. doi: 10.1097/EDE.0b013e31827623b1
36. Kagura J, Adair LS, Pisa PT, et al. Association of socioeconomic status change between infancy and adolescence, and blood pressure, in South African young adults: Birth to Twenty Cohort. *BMJ Open* 2016;6(3):e008805. doi: 10.1136/bmjopen-2015-008805
37. Krmar RT, Holtback U, Bergh A, et al. Oscillometric casual blood pressure normative standards for Swedish children using ABPM to exclude casual hypertension. *Am J Hypertens* 2015;28(4):459-68. doi: 10.1093/ajh/hpu182
38. Brettschneider AK, Ellert U, Schaffrath Rosario A. Comparison of BMI derived from parent-reported height and weight with measured values: results from the German KiGGS study. *International journal of environmental research and public health* 2012;9(2):632-47. doi: 10.3390/ijerph9020632 [published Online First: 2012/04/04]
39. Commission on Social Determinants of Health. CSDH Final Report: Closing the Gap in a Generation: Health Equity Through Action on the Social Determinant of Health. Geneva, World Health Organization, 2008.
40. OECD. In It Together: Why Less Inequality Benefits All. Paris, OECD Publishing, 2015. https://doi.org/10.1787/9789264235120-en/.
41. Juhola J, Magnussen CG, Viikari JS, et al. Tracking of serum lipid levels, blood pressure, and body mass index from childhood to adulthood: the Cardiovascular Risk in Young Finns Study. *The Journal of pediatrics* 2011;159(4):584-90. doi: 10.1016/j.jpeds.2011.03.021 [published Online First: 2011/04/26]
42. Raitakari OT, Juonala M, Kahonen M, et al. Cardiovascular risk factors in childhood and carotid artery intima-media thickness in adulthood: the Cardiovascular Risk in Young Finns Study. *Jama* 2003;290(17):2277-83. doi: 10.1001/jama.290.17.2277 [published Online First: 2003/11/06]
43. Leon DA, Vagero D, Olausson PO. Social class differences in infant mortality in Sweden: comparison with England and Wales. *BMJ (Clinical research ed)* 1992;305(6855):687-91.
44. Kunst AE, Mackenbach JP. The size of mortality differences associated with educational level in nine industrialized countries. *Am J Public Health* 1994;84(6):932-7.
45. Pickett KE, Wilkinson RG. Child wellbeing and income inequality in rich societies: ecological cross sectional study. *BMJ (Clinical research ed)* 2007;335(7629):1080. doi: 10.1136/bmj.39377.580162.55 [published Online First: 2007/11/21]
46. Lundberg O. The next step towards more equity in health in Sweden: how can we close the gap in a generation? *Scand J Public Health* 2018;46(22_suppl):19-27. doi: 10.1177/1403494818765702 [published Online First: 2018/06/05]

For peer review only - http://bmjopen.bmj.com/site/about/guidelines.xhtml

Table 1. Characteristics of adolescents by hypertension and overweight status.

	Normal blood pressure		Elevated blood pressure		Not overweight		Overweight	
	N	%	N	%	N	%	N	%
Total	240	83.0	49	17.0	244	84.1	46	15.9
Sex								
Boy	105	43.8	27	55.1	109	44.7	24	52.2
Girl	135	56.2	22	44.9	135	55.3	22	47.8
Ethnicity								
Swedish	213	89.9	44	91.7	218	90.5	40	88.9
Foreign ethnicity	24	10.1	4	8.3	23	9.5	5	11.1
Physical activity								
– times per week								
>2	138	67.6	31	83.8	149	72.0	21	60.0
0-2	66	32.4	6	16.2	58	28.0	14	40.0
CRD								
0-1 item	88	44.4	14	40.0	94	46.5	8	25.0
2-5 items	110	55.6	21	60.0	108	53.5	24	75.0
BMI group of adolescent								
Not OW	213	88.8	31	63.3				
OW/OB	27	11.2	18	36.7				
Income								
High	94	39.5	16	33.3	99	40.9	12	26.7
Middle	111	46.6	26	54.2	112	46.3	25	55.6
Low	33	13.9	6	12.5	31	12.8	8	17.8
Maternal education								
Tertiary	138	58.2	20	41.7	144	59.8	14	31.1
<Tertiary	99	41.8	28	58.3	97	40.2	31	68.9
SEI								
Class I	59	24.6	5	10.2	60	24.6	4	8.7

For peer review only

For peer review only - http://bmjopen.bmj.com/site/about/guidelines.xhtml

Class II-IV	131	54.6	25	51.0	130	53.3	27	58.7
Class V	50	20.8	19	38.8	54	22.1	15	32.6
City of residence								
White-collar	132	55	23	46.9	137	56.1	18	39.1
Blue-collar	108	45	26	53.1	107	43.9	28	60.9
	M	SD	M	SD	M	SD	M	SD
Age at follow-up (years)	16.02	0.90	16.12	1.04	16.02	0.92	16.18	0.94
Height (m)	1.72	0.09	1.73	0.10	1.73	0.09	1.71	0.09
Birthweight (100g)	35.47	5.63	36.08	5.89	35.32	5.64	37.00	5.68

BMI, Body Mass Index; *CRD*, Cardiovascular risk diet index; *OW/OB*, Overweight or obese; *SEI*, Swedish socioeconomic classification index.

For peer review only

For peer review only - http://bmjopen.bmj.com/site/about/guidelines.xhtml

Table 2. Univariate analysis with odds ratios for cardiovascular risk factors and areas under the ROC-curve for SES variables (n = 298).

	Elevated blood pressure		Low HDL		High LDL		Overweight	
	OR	CI	OR	CI	OR	CI	OR	CI
Age at follow-up (Z-years)	1.12	0.83,1.51	1.12	0.79,1.59	0.99	0.63,1.53	1.19	0.87,1.63
Height (Z-m)	1.24	0.91,1.64	1.41	0.85,2.33	1.26	0.85,1.88	0.82	0.59,1.14
Gender								
Girl	1.0		1.0		1.0		1.0	
Boy	1.61	0.86,3.04	2.78	1.09,7.09	1.31	0.57,3.00	1.38	0.73,2.60
Birthweight (Z-100g)	1.11	0.80,1.54	1.75	1.19,2.59	0.72	0.46,1.14	1.37	0.99,1.91
Ethnicity								
Swedish	1.0		1.0		1.0		1.0	
Foreign	0.83	0.27,2.58	1.49	0.53,4.19	0.29	0.04,2.25	1.13	0.41,3.16
Physical activity – times/week								
>2	1.0		1.0		1.0		1.0	
0-2	0.39	0.15,1.05	0.93	0.41,2.26	0.68	0.28,1.67	1.60	0.72,3.55
CRD								
0-1 item	1.0		1.0		1.0		1.0	
2-5 items	1.12	0.49,2.61	2.41	0.95,6.15	1.41	0.63,3.16	2.80	1.06,7.41
BMI group of adolescent								
Not OW	1.0		1.0		1.0			
OW/OB	4.42	2.19,8.95	3.44	1.47,8.07	3.20	1.28,7.98		
Income								
(ROC)	0.537		0.627		0.545		0.580	
High	1.0		1.0		1.0		1.0	

For peer review only - http://bmjopen.bmj.com/site/about/guidelines.xhtml

Middle	1.35	0.68,2.67	0.88	0.36,2.16	1.08	0.42,2.77	1.90	0.91,3.97
Low	1.11	0.41,3.05	3.94	1.39,11.14	1.73	0.46,6.54	2.21	0.82,5.95
Maternal education (ROC)								
	0.578		0.610		0.564		0.641	
Tertiary	1.0		1.0		1.0		1.0	
<Tertiary	1.87	0.99,3.53	2.39	1.10,5.21	1.67	0.77,3.59	3.22	1.61,6.44
SEI								
(ROC)	0.623		0.540		0.573		0.602	
Class I	1.0		1.0		1.0		1.0	
Class II-IV	2.20	0.80,6.05	1.33	0.48,3.66	1.91	0.60,6.09	3.21	1.08,9.57
Class V	4.37	1.49,12.81	1.45	0.48,4.48	1.41	0.24,8.28	4.28	1.34,13.73
City of residence (ROC)	0.541		0.652		0.511		0.585	
White-collar	1.0		1.0		1.0		1.0	
Blue-collar	1.39	0.76,2.56	1.78	0.74,4.31	1.10	0.51,2.41	2.00	1.06,3.78

BMI, Body Mass Index; *CRD*, Cardiovascular risk diet index; *CI*, 95% confidence interval; *OR*, odds ratio; *OW/OB*, Overweight or obese; *ROC*, area under the receiver operating characteristic curve; *SEI*, Swedish socioeconomic classification index.

For peer review only

For peer review only - http://bmjopen.bmj.com/site/about/guidelines.xhtml

Table 3. Multivariate analysis with odds ratios for cardiovascular risk factors. Adjusted for SES and other explanatory variables (n = 298).

		Elevated blood pressure					
		Model 1		Model 2		Model 3	
		OR	CI	OR	CI	OR	CI
Income	High	1.00		1.00		1.00	
	Middle	0.85	0.39,1.85	0.85	0.38,1.89	0.75	0.32,1.75
	Low	0.60	0.20,1.81	0.69	0.22,2.12	0.60	0.17,1.96
Maternal education	Tertiary	1.00		1.00		1.00	
	<Tertiary	1.29	0.60,2.77	1.15	0.52,2.54	0.88	0.37,2.08
SEI	Class I	1.00		1.00		1.00	
	Class II-IV	2.21	0.77,6.34	2.25	0.78,6.52	2.09	0.70,6.27
	Class V	4.18	1.22,14.41	4.43	1,26,15.58	4.95	1.30,18.89
City of residence	White-collar	1.00		1.00		1.00	
	Blue-collar	1.12	0.58,2.14	1.18	0.61,2.30	1.05	0.52,2.13

		Low HDL (<0.40 mg/dL).					
		Model 1		Model 2		Model 3	
		OR	CI	OR	CI	OR	CI
Income	High	1.00		1.00		1.00	
	Middle	0.98	0.31,3.08	0.81	0.29,2.29	0.82	0.28,2.40
	Low	3.16	0.97,10.35	3.59	0.96,13.38	3.76	0.95,14.99
Maternal education	Tertiary	1.00		1.00		1.00	
	<Tertiary	2.56	0.99,6.62	2.13	0.80,5.71	1.85	0.67,5.14
SEI	Class I	1.00		1.00		1.00	
	Class II-IV	0.88	0.32,2.48	0.79	0.28,2.27	0.73	0.25,2.12
	Class V	0.53	0.14,2.07	0.45	0.11,1.92	0.43	0.10,1.92
City of residence	White-collar	1.00		1.00		1.00	
	Blue-collar	1.41	0.55,3.60	1.20	0.46,3.12	1.09	0.40,2.93

High LDL (>110 mg/dL)

For peer review only - http://bmjopen.bmj.com/site/about/guidelines.xhtml

		Model 1		Model 2		Model 3	
		OR	CI	OR	CI	OR	CI
Income	High	1.00		1.00		1.00	
	Middle	0.91	0.32,2.56	0.88	0.31,2.51	0.84	0.29,2.49
	Low	1.48	0.35,6.21	1.47	0.34,6.29	1.41	0.31,6.39
Maternal education	Tertiary	1.00		1.00		1.00	
	<Tertiary	1.86	0.70,4.93	1.81	0.68,4.79	1.58	0.58,4.32
SEI	Class I	1.00		1.00		1.00	
	Class II-IV	1.69	0.49,5.77	1.68	0.49,5.79	1.52	0.43,5.40
	Class V	0.92	0.10,8.56	0.97	0.10,9.06	0.90	0.09,9.54
City of residence	White-collar	1.00		1.00		1.00	
	Blue-collar	0.89	0.37,2.15	0.90	0.37,2.18	0.83	0.33,2.09

		Overweight status			
		Model 1		Model 2	
		OR	CI	OR	CI
Income	High	1.00		1.00	
	Middle	1.16	0.52,2.60	1.12	0.50,2.54
	Low	1.21	0.42,3.54	1.20	0.40,3.56
Maternal education	Tertiary	1.00		1.00	
	<Tertiary	2.63	1.20,5.78	2.39	1.08,5.30
SEI	Class I	1.00		1.00	
	Class II-IV	2.34	0.75,7.28	2.09	0.66,6.60
	Class V	1.90	0.52,6.98	1.63	0.43,6.25
City of residence	White-collar	1.00		1.00	
	Blue-collar	1.39	0.70,2.73	1.39	0.70,2.76

BMI, Body Mass Index; *CI*, 95% confidence interval; *OR*, odds ratio; *SEI*, Swedish socioeconomic classification index.

Model 1 includes all SES variables. Model 2 includes all SES and other variables excluding overweight status that reached the selection criteria in univariate analysis ($p \leq 0.05$ or OR <0.5 or >2.0 for categorical and <0.75 or >1.5 for continuous variables): physical activity for elevated blood pressure; gender, birthweight and cardiovascular risk diet for low HDL; ethnicity for LDL; cardiovascular risk diet for overweight. Model 3 includes all SES, other explanatory variables and overweight status.

For peer review only - http://bmjopen.bmj.com/site/about/guidelines.xhtml

Table 4. Association between maternal education level at birth and child overweight or obesity at four ages.

Time point:	2 years	5 years	8 years	12 years
Cohort approach				
Age: mean (SD)	2.72 (0.28)	5.32 (0.30)	7.86 (0.34)	12.61 (0.87)
Sample size available	1070	1070	1070	1070
Overweight: n (%)	149 (13.9%)	146 (13.6%)	157 (14.7%)	168 (15.7%)
Obese: n (%)	19 (1.8%)	27 (2.5%)	21 (2.0%)	21 (2.0%)
Tertiary v < Tertiary education: overweight*	0.95 (0.67, 1.34)	1.42 (1.00, 2.03)	1.36 (0.96, 1.92)	1.81 (1.28, 2.55)
Tertiary v < Tertiary education: obesity*	0.41 (0.15, 1.08)	3.21 (1.29, 8.02)	1.46 (0.60, 3.56)	3.89 (1.30, 11.63)
Available sample approach				
Age: mean (SD)	2.73 (0.30)	5.34 (0.31)	7.86 (0.38)	12.40 (0.91)
Sample size available	7925	6602	2998	3337
Overweight: n (%)	1187 (15.0%)	1096 (16.6%)	446 (14.9%)	527 (15.8%)
Obese: n (%)	191 (2.4%)	273 (4.1%)	71 (2.4%)	75 (2.2%)
Tertiary v < Tertiary education: overweight*	1.18 (1.03, 1.34)	1.30 (1.13, 1.50)	1.16 (0.94, 1.42)	1.70 (1.39, 2.06)
Tertiary v < Tertiary education: obesity*	1.56 (1.12, 2.17)	1.52 (1.15, 1.99)	2.28 (1.31, 3.95)	2.16 (1.29, 3.61)

*Odds ratio (95% confidence interval)

Note: The cohort approach utilises only participants with all necessary data recorded at all four ages whereas the available sample approach uses any participant with all necessary data recorded on at least one age.

For peer review only

Supplementary table 1: Comparison of original ABIS cohort, invited subsample, participants and non-participants in the cardiovascular risk study.

	Original cohort		Invited to subsample		Participants		Non- participants		p[1]	p[2]	p-[3]
	N=16349		N=578		N=298		N=240				
	Mean/N	SD/%	Mean/N	SD/%	Mean/N	SD/%	Mean/N	SD/%			
Birthweight (g)	3578.6	560.8	3552.6	553.6	3554.0	574.7	3551.1	530.8	0.27	0.46	0.95
Gender									0.11	0.05	0.23
Boys	8473	51.9 %	279	48.5	136	46.1	143	46.1 %			
Girls	7866	48.1 %	296	51.5	159	53.9	137	48.9 %			
Ethnicity									0.09	0.50	0.01
Swedish	14235	89.3 %	492	87.1 %	266	90.5 %	226	83.4 %			
Other	1711	10.7 %	73	12.9 %	28	9.5 %	45	16.6 %			
Father ow/ob[a]									0.19	0.54	0.51
Not ow/ob	5384	54.0 %	254	56.4 %	146	57.3 %	108	57.3 %			
Overweight	4003	40.1 %	164	36.4 %	94	36.9 %	70	35.9 %			
Obese	586	5.9 %	32	7.1 %	15	5.9 %	17	8.7 %			
Mother ow/ob[a]									0.95	0.22	0.01
Not ow/ob	7310	70.0 %	328	69.6 %	191	72.9 %	137	65.6 %			

For peer review only

	Overweight	2372	22.7 %	107	22.7 %	59	22.5 %	48	23.0 %			
	Obese	759	7.3 %	36	7.6 %	12	4.6 %	24	11.5 %			
Overweight at 2 yrs										0.46	0.75	0.71
	Not ow/ob	6890	85.0 %	307	83.7 %	187	84.2 %	120	82.8 %			
	Ow/ob	1217	15.0 %	60	16.3 %	35	15.8 %	25	17.2 %			
Overweight at 5 yrs										0.35	<0.01	<0.01
	Not ow/ob	5624	83.3 %	287	85.2 %	187	90.3 %	100	76.9 %			
	Ow/ob	1125	16.7 %	50	14.8 %	20	9.7 %	30	23.1 %			
Overweight at 8 yrs										0.76	0.51	0.10
	Not ow/ob	2566	85.1 %	2566	85.1 %	139	86.9 %	50	78.1 %			
	Ow/ob	450	14.9 %	450	14.9 %	21	13.1 %	14	21.9 %			
Overweight at 12 yrs										0.63	0.20	0.08
	Not ow/ob	2836	84.2 %	197	85.3 %	155	87.6 %	42	77.8 %			
	Ow/ob	534	15.8 %	34	14.7 %	22	12.4 %	12	22,2 %			
Maternal education										< 0.01	< 0.01	<0.01
	High	5062	31.8 %	233	41.3 %	160	54.4 %	73	27.0 %			
	Low	10882	68.2 %	331	58.7 %	134	45.6 %	197	73.0 %	197	73.0 %	
Income										< 0.01	< 0.01	< 0.01
	High	3239	20.0 %	153	26.7 %	114	38.6 %	39	14.0 %			
	Middle	9718	60.0 %	301	52.5 %	141	47.8 %	160	57.6 %			
	Low	3239	20.0 %	119	20.8 %	40	13.6 %	79	28.4 %			

For peer review only

p-value[1]= Invited to follow-up vs original cohort, T-test or Chi2 as appropriate
p-value[2]= Participants in follow-up vs original cohort, T-test or Chi2 as appropriate
p-value[3]=Participants vs non-participants in follow-up, T-test or Chi2 as appropriate
[a] Parental height and weight self-reported at child age 1 year
T-test or Chi2 as appropriate

For peer review only

Reporting checklist for cohort study.

Based on the STROBE cohort guidelines.

Instructions to authors

Complete this checklist by entering the page numbers from your manuscript where readers will find each of the items listed below.

Your article may not currently address all the items on the checklist. Please modify your text to include the missing information. If you are certain that an item does not apply, please write "n/a" and provide a short explanation.

Upload your completed checklist as an extra file when you submit to a journal.

In your methods section, say that you used the STROBE cohort reporting guidelines, and cite them as:

von Elm E, Altman DG, Egger M, Pocock SJ, Gotzsche PC, Vandenbroucke JP. The Strengthening the Reporting of Observational Studies in Epidemiology (STROBE) Statement: guidelines for reporting observational studies.

		Reporting Item	Page Number
Title	#1a	Indicate the study’s design with a commonly used term in the title or the abstract	1
Abstract	#1b	Provide in the abstract an informative and balanced summary of what was done and what was found	2
Background / rationale	#2	Explain the scientific background and rationale for the investigation being reported	4
Objectives	#3	State specific objectives, including any prespecified hypotheses	5
Study design	#4	Present key elements of study design early in the paper	6
Setting	#5	Describe the setting, locations, and relevant dates, including periods of recruitment, exposure, follow-up, and data collection	6-7

For peer review only

Eligibility criteria	#6a	Give the eligibility criteria, and the sources and methods of selection of participants. Describe methods of follow-up.	6-7
	#6b	For matched studies, give matching criteria and number of exposed and unexposed	n/a
Variables	#7	Clearly define all outcomes, exposures, predictors, potential confounders, and effect modifiers. Give diagnostic criteria, if applicable	7-10
Data sources / measurement	#8	For each variable of interest give sources of data and details of methods of assessment (measurement). Describe comparability of assessment methods if there is more than one group. Give information separately for for exposed and unexposed groups if applicable.	7-10
Bias	#9	Describe any efforts to address potential sources of bias	10
Study size	#10	Explain how the study size was arrived at	5
Quantitative variables	#11	Explain how quantitative variables were handled in the analyses. If applicable, describe which groupings were chosen, and why	7-10
Statistical methods	#12a	Describe all statistical methods, including those used to control for confounding	10
	#12b	Describe any methods used to examine subgroups and interactions	N/A
	#12c	Explain how missing data were addressed	10
	#12d	If applicable, explain how loss to follow-up was addressed	6-7
	#12e	Describe any sensitivity analyses	N/A
Participants	#13a	Report numbers of individuals at each stage of study—eg numbers potentially eligible, examined for eligibility, confirmed eligible, included in the study, completing follow-up, and analysed. Give information separately for for exposed and unexposed groups if applicable.	12-13
	#13b	Give reasons for non-participation at each stage	7
	#13c	Consider use of a flow diagram	N/A

For peer review only

Descriptive data	#14a	Give characteristics of study participants (eg demographic, clinical, social) and information on exposures and potential confounders. Give information separately for exposed and unexposed groups if applicable.	Table 1
	#14b	Indicate number of participants with missing data for each variable of interest	Table 1
	#14c	Summarise follow-up time (eg, average and total amount)	6
Outcome data	#15	Report numbers of outcome events or summary measures over time. Give information separately for exposed and unexposed groups if applicable.	12-13
Main results	#16a	Give unadjusted estimates and, if applicable, confounder-adjusted estimates and their precision (eg, 95% confidence interval). Make clear which confounders were adjusted for and why they were included	10-13
	#16b	Report category boundaries when continuous variables were categorized	8-10
	#16c	If relevant, consider translating estimates of relative risk into absolute risk for a meaningful time period	N/A
Other analyses	#17	Report other analyses done—e.g., analyses of subgroups and interactions, and sensitivity analyses	N/A
Key results	#18	Summarise key results with reference to study objectives	14
Limitations	#19	Discuss limitations of the study, taking into account sources of potential bias or imprecision. Discuss both direction and magnitude of any potential bias.	15-16
Interpretation	#20	Give a cautious overall interpretation considering objectives, limitations, multiplicity of analyses, results from similar studies, and other relevant evidence.	17-18
Generalisability	#21	Discuss the generalisability (external validity) of the study results	15-16
Funding	#22	Give the source of funding and the role of the funders for the present study and, if applicable, for the original study on which the present article is based	18

For peer review only

The STROBE checklist is distributed under the terms of the Creative Commons Attribution License CC-BY. This checklist was completed on 22. March 2019 using https://www.goodreports.org/, a tool made by the EQUATOR Network in collaboration with Penelope.ai

For peer review only

Inequalities in Cardiovascular risks among Swedish adolescents – ABIS a prospective cohort study

Journal:	*BMJ Open*
Manuscript ID	bmjopen-2019-030613.R2
Article Type:	Original research
Date Submitted by the Author:	05-Dec-2019
Complete List of Authors:	Andersson White, Pär; Linköping University, Dept. of Medicine and Health; Linköping University Hospital, Crown Princess Victoria Children´s Hospital<br>Ludvigsson, Johnny; Linkopings universitet, Div of Pediatrics, Dept of Clin Exp Medicin, Medical Faculty<br>Jones, Michael; Macquarie University<br>Faresjo, Tomas; Linköpings universitet Hälsouniversitetet
**Primary Subject Heading**:	Paediatrics
Secondary Subject Heading:	Epidemiology, Cardiovascular medicine, Public health
Keywords:	PUBLIC HEALTH, EPIDEMIOLOGY, Cardiac Epidemiology < CARDIOLOGY, Paediatric endocrinology < DIABETES & ENDOCRINOLOGY, SOCIAL MEDICINE

SCHOLARONE™ Manuscripts

I, the Submitting Author has the right to grant and does grant on behalf of all authors of the Work (as defined in the below author licence), an exclusive licence and/or a non-exclusive licence for contributions from authors who are: i) UK Crown employees; ii) where BMJ has agreed a CC-BY licence shall apply, and/or iii) in accordance with the terms applicable for US Federal Government officers or employees acting as part of their official duties; on a worldwide, perpetual, irrevocable, royalty-free basis to BMJ Publishing Group Ltd ("BMJ") its licensees and where the relevant Journal is co-owned by BMJ to the co-owners of the Journal, to publish the Work in this journal and any other BMJ products and to exploit all rights, as set out in our licence.

The Submitting Author accepts and understands that any supply made under these terms is made by BMJ to the Submitting Author unless you are acting as an employee on behalf of your employer or a postgraduate student of an affiliated institution which is paying any applicable article publishing charge ("APC") for Open Access articles. Where the Submitting Author wishes to make the Work available on an Open Access basis (and intends to pay the relevant APC), the terms of reuse of such Open Access shall be governed by a Creative Commons licence – details of these licences and which Creative Commons licence will apply to this Work are set out in our licence referred to above.

Other than as permitted in any relevant BMJ Author's Self Archiving Policies, I confirm this Work has not been accepted for publication elsewhere, is not being considered for publication elsewhere and does not duplicate material already published. I confirm all authors consent to publication of this Work and authorise the granting of this licence.

For peer review only

Inequalities in Cardiovascular risks among Swedish adolescents – ABIS a prospective cohort study

Pär Andersson White[1, 2], Johnny Ludvigsson[2], Michael P. Jones[3], Tomas Faresjö[1]

[1]Dept. of Medicine and Health, Div. of Community Medicine, Linköping University, Sweden.

[2]Crown Princess Victoria Children´s Hospital and Div. of Pediatrics, Dept. of Clinical and Experimental Medicine, Linköping University, Sweden.

[3]Psychology Department, Macquarie University, North Ryde, NSW, 2109, Australia.

Corresponding author: Pär Andersson White, C/O T Faresjo, Dept. of Medicine and Health, Div. of Community Medicine, Linköping University, SE-581 83 Linköping Sweden, +4610-1030000, par.andersson.white@liu.se

Keywords: Socioeconomic status, Inequalities, Adolescent Overweight, Childhood overweight, Childhood obesity, Blood Pressure, Dyslipidemia, High-Density Lipoproteins, Low-Density Lipoproteins, ABIS

For peer review only - http://bmjopen.bmj.com/site/about/guidelines.xhtml

Abstract

OBJECTIVES: To investigate if Socioeconomic Status (SES) is predictive of cardiovascular risk factors among Swedish adolescents. Identify the most important SES variable for the development of each cardiovascular risk factor. Investigate at what age SES inequality in overweight and obesity occurs.

DESIGN: Longitudinal follow-up of a prospective birth cohort.

SETTING: All Babies in Southeast Sweden (ABIS) study includes data from children born between October 1997 and October 1999 in five counties of south east Sweden.

PARTICIPANTS: A regional ABIS-study subsample from three major cities of the region n=298 adolescents aged 16-18 years, and prospective data from the whole ABIS cohort for overweight and obesity status at the ages 2, 5, 8 and 12 years (n= 2998-7925).

OUTCOME MEASURES: Blood pressure above the hypertension limit, overweight/obesity according to the International Obesity Task Force (IOTF) definition, low high-density lipoproteins (HDL) or borderline-high low-density lipoproteins (LDL) according to National Cholesterol Education Program (NCEP) expert panel on cholesterol levels in children.

RESULTS: For three out of four cardiovascular risk outcomes (elevated blood pressure, low HDL and overweight/obesity) there were increased risk in one or more of the low SES groups ($p < 0.05$). The best predictor was parental occupational class (SEI) for elevated blood pressure (Area under the ROC curve 0.623), maternal educational level for overweight (Area under the ROC curve 0.641) and blue-collar city of residence for low HDL (Area under the ROC curve 0.641). SES related differences in overweight/obesity were found at age 2, 5 and 12 and for obesity at age 2, 5, 8, and 12 years (all $p<0.05$).

For peer review only

CONCLUSIONS: Even in a welfare state like Sweden, SES inequalities in cardiovascular risks are evident already in childhood and adolescence. Intervention programs to reduce cardiovascular risk based on social inequality should start early in life.

Strengths and limitations of this study:

- Data derives from a large prospective cohort representative for the general population.
- A standardized procedure for collection of clinical data.
- The Swedish National registers give us valid and reliable data on household income.
- The limited sample size of the follow–up group gives some restraints.

For peer review only

For peer review only - http://bmjopen.bmj.com/site/about/guidelines.xhtml

Introduction

Cardiovascular disease in adults has been shown to be one of the disease groups most correlated with socioeconomic inequalities in the world [1], and this has also been evident in Swedish adults [2]. Longitudinal studies have shown that risk factors for cardiovascular disease develop in childhood and that early signs of cardiovascular disease risks are visible in early adulthood [3-5]. The risk factors associated with cardiovascular disease include blood pressure, dyslipidemia and overweight. Of these risk factors, overweight and obesity are the ones that progress most consistently from childhood to adulthood [6]. Adolescent overweight has been shown to be a stronger predictor of cardiovascular mortality in adulthood than adult overweight [7].

Recent studies report lower scores than ideal in a range of cardiovascular health factors (blood pressure, cholesterol and glucose level below the American heart association's cut-offs) in European adolescents with low maternal education [8]. In Korea high income was protective against cardiovascular disease risk factors and in Finland low socioeconomic status (SES) measured by occupation of the father was associated with higher low-density lipoprotein (LDL) levels in boys and higher systolic blood pressure and higher percentage of smokers [9 10]. However, longitudinal data on the relationships between childhood SES and adolescent's cardiovascular health is still scarce.

An increase in physical activity has been shown to reduce the risk of being overweight and obese as a youth in Sweden and other European countries [11]. Studying the effect of diet on development of overweight and obesity in children and adolescents has yielded no clear conclusions on whether any specific foods are important for the development of obesity [12]. However, there is some evidence for intake of certain foods and development of adult

For peer review only - http://bmjopen.bmj.com/site/about/guidelines.xhtml

cardiovascular disease. The INTERHEART study found a positive association between intake of meat, salty foods, fried foods and development of cardiovascular disease and a negative association between intake of fruits and vegetables and cardiovascular disease risk [13].

Sweden currently ranks as 9th in the Organization for Economic Co-operation and Development (OECD) list of economic equality [14]. It has a comprehensive public health system with a number of interventions aimed at reducing social inequalities. These include universal and public financed healthcare equally accessible for all. The healthcare system provides free antenatal consulting and check-ups at Child Health Care Centers which covers advice on nutrition and health from professional nurses for all children at regular intervals up to school age. Other interventions include school-health check-ups and mandatory physical activity in schools and free school lunches for all children. Other support for families with children includes paid maternity leave for 480 days and heavily subsidized daycare [15 16]. Can we despite these interventions and the relatively low-income inequality, find SES related differences in cardiovascular risk among Swedish adolescents?

Aims

The aims of this study were to determine whether SES is predictive of cardiovascular risk factors in Swedish adolescents and to identify which socioeconomic factors that were most important for the development of each risk factor. Further, to analyze at what ages socioeconomic differences in overweight and obesity occur using longitudinal data from follow-ups in the prospective All Babies In Southeast Sweden (ABIS) study.

Material and Methods

For peer review only

For peer review only - http://bmjopen.bmj.com/site/about/guidelines.xhtml

Study population

The ABIS study is a prospective population-based cohort study, initially including 17 055 children born between October 1st, 1997 and October 1st 1999 in southeast Sweden in the counties of Östergötland, Jönköping, Kronoberg, Kalmar, and Blekinge. The ABIS region is inhabited by approx. 1.25 million out of Sweden's 10 million inhabitants[17]. The region incorporates three of the biggest cities of the country, Linköping (5th), Norrköping (9th) and Jönköping (10th), but also large rural areas, both farmland and forest areas. In terms of income inequality, the counties of southeast Sweden are placed close to the average of the country between the most unequal areas (the capital Stockholm) and the northern areas with the lowest income inequality[18]. This cohort of children has been followed from birth and are still being followed with respect to biological samples, questionnaires and register data. The initial aim of the ABIS study was to analyse the development of immune-mediated diseases including autoimmune diseases, type 1 and type 2 diabetes as well as cardiovascular disease, and the effect of different environmental and genetic factors.

We undertook a follow-up of a regional subsample of ABIS children and also made a questionnaire-based longitudinal analysis for the whole cohort. Inclusion criteria for the regional subsample was residence in one of the three major cities of Östergötland County. Out of n=578 children invited n=298 (51.6%) participated. The main reasons for non-participation among the invited sample were practical i.e. difficulties to arrange health check-ups for our research nurse at some schools that lacked school-nurse facilities. The study participants filled out a questionnaire about their lifestyle, diet and physical activity. A specially trained research nurse undertook all data collection including blood pressure

For peer review only - http://bmjopen.bmj.com/site/about/guidelines.xhtml

measurement, questionnaire and collection of blood samples and measurement of anthropometric data.

Blood Pressure

Systolic and diastolic blood pressure was measured manually by the research nurse at the participant's school nursing offices. Study participants rested on a bed at least 5 minutes before measurement, two consecutive measurements of blood pressure were then taken in the sitting position and the mean value was calculated. Blood pressures were grouped as hyper- or normotensive according to the guidelines described in the fourth report on the diagnosis, evaluation, and treatment of high blood pressure in children and adolescents [19]. These guidelines define hypertension as an average systolic and/or diastolic blood pressure ≥95th percentile for gender, age and height percentile according to the Center of Disease Control and Preventions growth charts on three or more occasions [20].

Overweight/obesity

The participating adolescents' height (cm) and weight (kg) were measured by the research nurse. Data on weight and height were missing for 8 participants. Body mass index (BMI) was calculated as BMI = weight(kg)/height(m)^2 and dichotomized as overweight/obese or not, using the International obesity task force latest age and gender specific cut-offs defined by Cole et al [21 22]. The same procedure was then used for parental-reported height and weight of the child available from previous ABIS follow-ups at age 2, 5, 8 and 12 years.

Dyslipidemia

Blood samples for blood lipid analysis were collected from 242 (81.2 %) individuals and analyzed for plasma levels of LDL and HDL at the Department of clinical chemistry, Linköping

For peer review only - http://bmjopen.bmj.com/site/about/guidelines.xhtml

University Hospital, according to accredited procedures. One sample was missing data on LDL after analysis. Test results were converted from SI units to mg/dL. Results were then grouped as normal or low HDL cholesterol in accordance with the cut-off for low HDL cholesterol (<40 mg/dL) set by the Expert panel on integrated guidelines for cardiovascular health and risk reduction in children, which is based on the 10th percentile found by the National cholesterol educational program (NCEP) [6 23]. For LDL only 8 (3.3%) out of 241 had a level above the limit of high LDL (the 95th percentile in NCEP). As this number was insufficient for statistical analyses the cut-off of borderline high LDL (>110 mg/dL, the 75th percentile in NCEP) was chosen as indicator of increased LDL.

Socioeconomic variables

A five grade Socioeconomic Class (SEI) variable was constructed based on parents occupation and the occupational groups used by Statistics Sweden in their Swedish socioeconomic classification index[24]. The definitions for each class was; Class I Professionals (SEI 54-60), Class II Intermediate non-manual employees (SEI 44-46), Class III Assistant non-manual employees (SEI 33-36), Class IV Skilled workers (SEI 21-22) and Class V Unskilled workers (SEI 11-12). Occupations in SEI 76-87 which includes non-professional self-employees including farmers were given a Class I-V based on their educational attainment. The adolescents were classified according to the highest occupational class of either of their parents. Mother's educational level was derived from the ABIS birth questionnaire and grouped as primary/secondary education or post-secondary education/university. Family disposable income was derived from Statistics Sweden for the year 2012 and grouped into three groups; low income representing the bottom 20 % of household disposable incomes in

For peer review only - http://bmjopen.bmj.com/site/about/guidelines.xhtml

the original cohort (available for n=16196), middle income representing the middle 20 to 80 percentiles of income and top income representing the top 20 percent of income.

The three included cities in this study represent different social history and different socioeconomic environments [25]. The participants from the city of Motala and Norrköping, are towns with a mainly industrial history, and were merged in the analysis and labelled as blue-collar cities. The city of Linköping has a history as a political and ecclesiastical administrative centre of the county, was labelled as white-collar city. Today this city has a workforce of mainly civil servants including high tech industries and one of Sweden's major universities. Previous studies of these cities has exhibit marked public health differences, including higher mortality in cardiovascular disease among adults in the blue-collar cities and difference in life-expectancy [26 27].

Covariates

Age was calculated using the date of answering the questionnaire and the participants birth date. Height was measured at follow-up by a research nurse. Ethnicity was based on the question "Are you (the mother) born in Sweden" and "Is the father born in Sweden". The adolescents were then grouped as having foreign ethnicity if one of the parents was born outside of Sweden. A cardiovascular risk diet index (CRD) was constructed based on the dietary components most associated with myocardial infarction in the INTERHEART study [13]. These factors were: intake of salty foods one time per day or more, intake of deep-fried foods three times or more per week, intake of meat two or more times per day, intake of fruit less than once per day and intake of vegetables less than once per day. This score which has a range of 0 to 5 was then dichotomized into two groups Low CRD (0-1 items

For peer review only - http://bmjopen.bmj.com/site/about/guidelines.xhtml

n=101) and High CRD (2 to 5 items n=138). Degree of physical activity was measured by the question “How many times per week do you do physical activity at least 30 min that is strenuous enough to make you sweat”. The answers were then grouped into two groups; 0-2 times a week, >2 times a week.

Patient and Public Involvement

Patients and public were not involved in the design of this study. The results will be disseminated to the study participants through the ABIS study homepage https://www.abis-studien.se/.

Statistical analysis

Data were analyzed using bivariate logistic regression for each cardiovascular risk factor. As analysis of missing values showed significant differences due to SES, adolescents with low maternal educational level had a significantly higher number of missing values in questionnaire data, multiple imputation was used for missing values to reduce this potential bias. Multiple imputation was employed as implemented in the SPSS statistics program. A Markov Chain Monte Carlo was employed with predictive models selected according to the measurement scale of each dependent variable specified in five imputation samples. A univariate logistic regression was then analyzed for each independent variable (SES variables and covariates) and Odds Ratios (OR) with 95% confidence interval for each dependent variable (elevated blood pressure, low-HDL, high-LDL and overweight/obesity at last follow-up) were calculated. For further multivariate analysis all four SES variables (maternal education, income, SEI, white/blue collar city) were included on a priori basis while other

For peer review only - http://bmjopen.bmj.com/site/about/guidelines.xhtml

independent variables were chosen based on reaching statistical significance $p \leq 0.05$ or an effect size of OR <0.5 or >2.0 for categorical variables. For continuous variables; height and age, was standardized and the OR for 1 standard deviation increase of <0.75 or >1.5 was used as selection criteria. This approach was adopted due to limited sample size and, in this context, effect size being as important from a public health perspective as statistical significance.

The degree of discrimination afforded by each socioeconomic variable was quantified by the area under the receiver operating characteristic (ROC) curve defined by taking the logistic model predicted probability of adverse health state as the test measure and the actual state as an imperfect reference standard. Values >0.5 indicate some discrimination with values close to 1.0 indicating excellent discrimination.

A hierarchical approach was adopted in which SES variables were first adjusted for other SES variables, then for other independent variables reaching the selection criteria and finally in three of the four outcomes (elevated blood pressure, low LDL and high LDL) for adolescent's overweight status.

Bivariate logistic regression was also used in the final analysis on the relationship between overweight/obesity and maternal educational level at four different time points. The first analysis used a cohort approach and included only subjects with complete data from all follow-ups (n = 1070). The second analysis used an available sample approach and included all subjects that had data for any specific age (n=2995-7925).

Statistical analysis was performed using Statistical Package for the Social Sciences v 23 (SPSS, Chicago, IL, USA).

For peer review only

Ethical considerations

The ABIS-study has been approved by the Research Ethics committees of the Faculty of Health Science at Linköping University and Lund university. Dnr 03-092, Dnr 99-227 and Dnr 99-321. All participants in the follow-up gave their informed consent as well as their caregivers consent for those below 18 years of age.

Results

The participants in our follow-up had a mean age of 16.05 (SD 0.9) years (n=136 males, n=162 females). They were distributed between the cities of Linköping n=160 (54%), Norrköping n=91 (30%) and Motala n=47 (16%). 24 (9.7 %) out of 248 were categorized as having foreign ethnicity. The invited subsample was similar to the original ABIS cohort in respect to a number of variables (birthweight, ethnicity, parental overweight and obesity, child overweight at age 2, 8 and 12 years). However, comparison of this ABIS follow-up with the original ABIS cohort showed a higher proportion of mothers with high education and families with high income and a lower proportion with overweight at 5 years. There was also a slight difference (p=0.052) in gender composition with boys being underrepresented in the subsample. Non-participation analysis also showed underrepresentation of children with foreign ethnicity, low maternal education and low household income (see supplementary table 1).

The total number of participants that were classified as having elevated blood pressure was 49/290 (16.9 %), see table 1. This was based on 34/290 participants (11.7%) with systolic blood pressures above the limit for hypertension and 17/290 (5.9 %) with diastolic blood pressure above the limit. Two individuals were above both systolic and diastolic hypertension limits. An HDL level below 40 mg/dL was found in 30 (12.4 %) out of 242

For peer review only - http://bmjopen.bmj.com/site/about/guidelines.xhtml

samples. For LDL 25/241 (10.4 %) were above the cut-off for borderline-high value (110 mg/dL).

Elevated blood pressure

All SES variables were associated with increased odds ratios (OR) for elevated blood pressure in the lowest SES groups compared with the highest, see table 2. Out of the four SES variables, the parental occupational class variable SEI had the highest area under the ROC curve, 0.623 compared to 0.537-0.578 for the other SES variables. The lowest social class had an OR of 4.37 (95% confidence interval 1.49, 12.81) compared to the highest class.

After adjustments for all other SES variables, physical activity and overweight status the lowest SEI class had an OR for elevated blood pressure of 4.95 (95% confidence interval 1.30, 18.89), see table 3.

Dyslipidemia

All indicators of low SES showed an increased odd ratio of having low HDL. Out of the four variables, white/blue-collar city was associated with the highest area under the ROC curve, 0.652 compared to 0.540-0.627 for the other SES variables. Blue-collar city was associated with an OR of 1.78 (95% confidence interval 0.74, 4.31) of low HDL. Low maternal education and low income were both associated with significantly increased risks of low HDL, low maternal education with an OR of 2.39 (95% confidence interval 1.10, 5.21) and low income with an OR of 3.94 (95% confidence interval 1.39, 11.14) for having low HDL.

After adjustment for all other SES variables, gender, CRD and overweight the OR for blue-collar city was reduced to 1.22 (95% confidence interval 0.46, 3.27).

For peer review only - http://bmjopen.bmj.com/site/about/guidelines.xhtml

For LDL the difference between low and high SES groups were small, no SES variable reached an OR above 2.0, and no variable reached statistical significance $p \leq 0.05$. The area under the ROC curve ranged from 0.573 for SEI to 0.511 for white/blue-collar city.

Overweight/obesity

In the regional subsample 46/290 (15.8 %) were overweight or obese, with 7 (2.4 %) obese. All four measures of low SES had an increased odds ratio of being overweight or obese. The best discriminator measured as area under the ROC curve for a single SES variable was 0.641 for maternal education. The only other variable that met the criteria for being included in the multivariate analysis was diet according to CRD.

After adjustment for all other SES variables and CRD maternal education had an odds ratio of 2.39 (95% confidence interval 1.08, 5.30) for being overweight.

Overweight and obesity inequality during childhood

Overweight and obesity differences related to maternal education were measured longitudinally at four different time-points. In the analysis on subjects with complete data (cohort approach) the children to low educated mothers´ had significantly increased odds of being overweight/obese at age 12, but not at age 2, 5 and 8. The analysis based on all available data (available sample approach) showed significant differences at age 2, 5 and 12 but not at age 8. For obesity there were significant differences at age 5 and 12 in the cohort approach, while the available sample approach showed significant differences at all ages, 2, 5, 8 and 12 years, see table 4.

For peer review only - http://bmjopen.bmj.com/site/about/guidelines.xhtml

Discussion

There was significantly increased risk of three out of four cardiovascular risk factors due to low SES in Swedish adolescents. Parental occupational class according to SEI was the most important factor for risk of elevated blood pressure. Although overweight was important for elevated blood pressure the independent importance of parental occupational class was further strengthened as it remained after adjustment for all other SES factors, physical activity and overweight. For other outcomes we found maternal education to be the best discriminator for overweight and white/blue collar city for low HDL (<40 mg/dL).

In the Whitehall studies, occupational class and blood pressure were found to be closely associated in adults [28]. One finding of the Whitehall II study is that workers with low occupational grade have increased blood pressure during the working day, but no difference in resting blood pressure [29]. As blood pressure in this study was measured while the adolescents' were attending school it is possible that it represents the same kind of increase in response to school tasks in low SES adolescents as that seen during the work day in adult workers.

The impact of maternal education on child overweight has been shown in large multi cohort studies [30]. In addition to being a marker for SES like occupation and income the impact of maternal education could involve differences in knowledge. There is evidence that adolescents with mothers with good nutritional knowledge have a healthier diet [31]. High maternal education has also been associated with a higher degree of breakfast eating in adolescents [32]. Further, low parental education has been associated with increased access to sugar sweetened drinks and low vegetable intake [33]. These factors could be part of the

For peer review only - http://bmjopen.bmj.com/site/about/guidelines.xhtml

explanation of why maternal educational level is more important than other SES factors for overweight status in adolescents.

White/blue-collar city was the best discriminator of low HDL status. After adjustment for other SES variables the odds ratio for blue-collar cities was reduced to OR 1.35 (0.53, 3.48), adjusting for diet and overweight further decreased the OR to 1.22 (0.46, 3.27) indicating that most of the risk associated with blue-collar residence is due to differences in SES, diet and overweight. Thus, our results are in line with previous studies [25].

The main limitation of this study is the limited number of participants of the follow-up part (n=298). Even though the sample size of study is somewhat restrained we managed to capture SES related significant differences in cardiovascular risks. Our sample is quite representative concerning overweight status compared to the previous larger samples in ABIS and to other Swedish studies [34]. Although the number of low income families were similar in our invited subsample compared to the original cohort there was an overrepresentation of high maternal education and high income families, this most likely reflects the fact that the subsample is from a more urban population than the rest of the ABIS region and must be taken into consideration when interpreting the result. Among the invited, the number of adolescents with high maternal education at birth and high income families were higher among the participants of the study than in the non-participant group and the same was true for high income families. This is a common finding in longitudinal studies, but as previous studies have shown, the effect of this underrepresentation in low SES groups, if anything, is to underestimate inequality slightly in later follow-ups and have little effect on the qualitative conclusion on inequality [35]. The number of adolescents with elevated blood pressure was quite high (16.9%) and as blood pressure was measured only at

For peer review only - http://bmjopen.bmj.com/site/about/guidelines.xhtml

one visit to school by the research nurse some of the adolescents might have had White-coat rather than true hypertension [19]. However, other studies have shown similarly high values in other populations [36]. A recent study in a Swedish cohort also found higher average systolic blood pressure in the age group 14-16 years than was found in the fourth report on the diagnosis, evaluation, and treatment of high blood pressure in children and adolescents [37]. We could not rule out that White-coat hypertension might be related to socioeconomic factors but have no evidence of that.

The fact that the longitudinal data was based on parental-reported height and weight might affect the accuracy of our results. A German study found that parental reported data tends to overestimate the prevalence of overweight in young children and underestimate it in older children, however the same study found the impact of SES on this error to be small.[38] Thus, although the exact prevalence of overweight status in our study might differ slightly from the true prevalence the relative difference between SES groups should not.

Different socioeconomic variables can have different effects on health. It is a strength that we investigated not one but four dimensions of SES inequality. These dimensions seem to be of varying importance for cardiovascular risk outcomes. Thus our study is an example of the many layers of the social determinants of health that give rise to an individual's risk of disease [39]. Although income, maternal education and occupational status are correlated, it is not surprising that the strongest discriminators for cardiovascular risk factors are occupational class, blue collar city and educational level rather than income in this Swedish cohort, as income inequality still is low compared to most other countries [40]. However, our results show that socioeconomic differences in overweight and obesity could be found already in early childhood also in Sweden.

For peer review only - http://bmjopen.bmj.com/site/about/guidelines.xhtml

For peer review only

The implication of adolescent SES differences on adult life cardiovascular health inequalities could be important. Longitudinal data from Finland have shown that cardiovascular risk factors such as elevated blood pressure, serum lipids and obesity tracks to adulthood, increasing the risk of adult abnormalities [41]. Data from the same cohort also shows that adolescent blood pressure and LDL cholesterol are risk factors of adult carotid intima media thickening independent of adult risk factors [42]. A general awareness in primary care is important of the potential risk of elevated blood pressure and other cardiovascular risks in low SES adolescents.

Sweden has for a long time been considered one of the more egalitarian countries in the world and has often been used for comparison in studies on health inequalities as a good example [43 44]. Yet during the last two decades this has started to change with economic inequality increasing faster than in other OECD countries and Sweden has gone from a top position in equality to now being the 9th country in income equality out of the 36 countries in OECD [14]. Previous studies have found that for health problems with higher prevalence in low SES groups, like cardiovascular disease in adults, both the over-all prevalence and absolute inequality between high and low SES groups increases with larger economic inequality [1 45]. Our finding that cardiovascular risks in children and adolescents have a SES gradient also in Sweden could have serious public health implication for the future, as the rapid rise of economic inequality might increase absolute inequalities and prevalence of cardiovascular risk factors in children and adolescents. Concerns for health inequalities have increased in recent years spurring the Swedish government to start a Commission for health equality with the aim of finding ways to decrease these inequalities [46].

Conclusion

For peer review only - http://bmjopen.bmj.com/site/about/guidelines.xhtml

Despite an extensive welfare system and public health interventions in Sweden, cardiovascular health inequality is evident among Swedish youths and children. Preventive health services and clinicians must be aware of the increased risk of cardiovascular risk factors in low SES children and adolescents and improve prevention programs in this group. Policies aimed at decreasing the evident inequalities in cardiovascular disease in adults should start early and include interventions already in childhood and adolescence.

For peer review only

For peer review only - http://bmjopen.bmj.com/site/about/guidelines.xhtml

Funding

The ABIS-study has been supported by Swedish Research Council (K2005-72X-11242-11A and K2008-69X-20826-01-4) and the Swedish Child Diabetes Foundation (Barndiabetesfonden), JDRF Wallenberg Foundation (K 98-99D-12813-01A), Medical Research Council of Southeast Sweden (FORSS) and the Swedish Council for Working Life and Social Research (FAS2004–1775) and Östgöta Brandstodsbolag. The Research and PhD studies Committee (FUN), Linköping University, Sweden (LiU-).

Competing interests

None declared.

Data sharing statement:

Data from the ABIS registry, including study protocols, report forms and consents may be obtained through requests to the PI and project coordinator Senior professor Johnny Ludvigsson email: Johnny.Ludvigsson@liu.se

Author Contributions

TF, JL and PAW were responsible for the study concept and design. JL is the principal investigator for ABIS. PAW did the statistical analysis with advice from MJ. All authors were responsible for analysis and interpretation of data. PAW drafted the manuscript and TF, JL and MJ undertook critical revision of the manuscript. PAW has full access to all of the data in the study, takes responsibility for the integrity of the data and accuracy of the data analysis and had the final responsibility to submit for publication.

For peer review only

References

1. Wilkinson RG, Pickett KE. Income inequality and socioeconomic gradients in mortality. *Am J Public Health* 2008;98(4):699-704. doi: 10.2105/AJPH.2007.109637
2. Toch-Marquardt M, Menvielle G, Eikemo TA, et al. Occupational class inequalities in all-cause and cause-specific mortality among middle-aged men in 14 European populations during the early 2000s. *PLoS One* 2014;9(9):e108072. doi: 10.1371/journal.pone.0108072
3. Berenson GS. Childhood risk factors predict adult risk associated with subclinical cardiovascular disease. The Bogalusa Heart Study. *Am J Cardiol* 2002;90(10C):3L-7L.
4. Mahoney LT, Burns TL, Stanford W, et al. Coronary risk factors measured in childhood and young adult life are associated with coronary artery calcification in young adults: the Muscatine Study. *Journal of the American College of Cardiology* 1996;27(2):277-84. [published Online First: 1996/02/01]
5. Puolakka E, Pahkala K, Laitinen TT, et al. Childhood Socioeconomic Status and Arterial Stiffness in Adulthood: The Cardiovascular Risk in Young Finns Study. *Hypertension* 2017;70(4):729-35. doi: 10.1161/HYPERTENSIONAHA.117.09718
6. Expert Panel on Integrated Guidelines for Cardiovascular H, Risk Reduction in C, Adolescents, et al. Expert panel on integrated guidelines for cardiovascular health and risk reduction in children and adolescents: summary report. *Pediatrics* 2011;128 Suppl 5:S213-56. doi: 10.1542/peds.2009-2107C
7. Must A, Jacques PF, Dallal GE, et al. Long-term morbidity and mortality of overweight adolescents. A follow-up of the Harvard Growth Study of 1922 to 1935. *N Engl J Med* 1992;327(19):1350-5. doi: 10.1056/NEJM199211053271904
8. Henriksson P, Henriksson H, Labayen I, et al. Correlates of ideal cardiovascular health in European adolescents: The HELENA study. *Nutrition, metabolism, and cardiovascular diseases : NMCD* 2018;28(2):187-94. doi: 10.1016/j.numecd.2017.10.018
9. Leino M, Porkka KV, Raitakari OT, et al. Influence of parental occupation on coronary heart disease risk factors in children. The Cardiovascular Risk in Young Finns Study. *Int J Epidemiol* 1996;25(6):1189-95.
10. Seo YG, Choi MK, Kang JH, et al. Cardiovascular disease risk factor clustering in children and adolescents: a prospective cohort study. *Archives of disease in childhood* 2018;103(10):968-73. doi: 10.1136/archdischild-2017-313226 [published Online First: 2018/04/14]
11. Janssen I, Katzmarzyk PT, Boyce WF, et al. Comparison of overweight and obesity prevalence in school-aged youth from 34 countries and their relationships with physical activity and dietary patterns. *Obes Rev* 2005;6(2):123-32. doi: 10.1111/j.1467-789X.2005.00176.x
12. Pate RR, O'Neill JR, Liese AD, et al. Factors associated with development of excessive fatness in children and adolescents: a review of prospective studies. *Obes Rev* 2013;14(8):645-58. doi: 10.1111/obr.12035
13. Iqbal R, Anand S, Ounpuu S, et al. Dietary patterns and the risk of acute myocardial infarction in 52 countries: results of the INTERHEART study. *Circulation* 2008;118(19):1929-37. doi: 10.1161/circulationaha.107.738716 [published Online First: 2008/10/22]
14. OECD. Key indicators on the distribution of household disposable income and poverty, 2007, 2015 and 2016 or most recent year. [Available from: http://www.oecd.org/social/soc/IDD-Key-Indicators.xlsx accessed 4 november 2019.
15. Wettergren B, Blennow M, Hjern A, et al. Child Health Systems in Sweden. *The Journal of pediatrics* 2016;177s:S187-s202. doi: 10.1016/j.jpeds.2016.04.055 [published Online First: 2016/09/27]
16. Försäkringskassan. When the child is born [Available from: https://www.forsakringskassan.se/privatpers/foralder/nar_barnet_ar_fott/ accessed 4 november 2019.

For peer review only - http://bmjopen.bmj.com/site/about/guidelines.xhtml

17. Statistics Sweden. Population in the country, counties and municipalities on 31 March, 2019 and population change in January–March 2019 [Available from: https://www.scb.se/en/finding-statistics/statistics-by-subject-area/population/population-composition/population-statistics/pong/tables-and-graphs/quarterly-population-statistics--municipalities-counties-and-the-whole-country/quarter-1-2019/ accessed 4 november 2019.
18. Statistics Sweden. Income and tax statistics [Available from: https://www.scb.se/en/finding-statistics/statistics-by-subject-area/household-finances/income-and-income-distribution/income-and-tax-statistics/ accessed 4 november 2019.
19. National High Blood Pressure Education Program Working Group on High Blood Pressure in C, Adolescents. The fourth report on the diagnosis, evaluation, and treatment of high blood pressure in children and adolescents. *Pediatrics* 2004;114(2 Suppl 4th Report):555-76.
20. Gibbs BG, Forste R. Socioeconomic status, infant feeding practices and early childhood obesity. *Pediatr Obes* 2014;9(2):135-46. doi: 10.1111/j.2047-6310.2013.00155.x
21. Cole TJ, Bellizzi MC, Flegal KM, et al. Establishing a standard definition for child overweight and obesity worldwide: international survey. *BMJ (Clinical research ed)* 2000;320(7244):1240-3.
22. Cole TJ, Lobstein T. Extended international (IOTF) body mass index cut-offs for thinness, overweight and obesity. *Pediatr Obes* 2012;7(4):284-94. doi: 10.1111/j.2047-6310.2012.00064.x
23. National Cholesterol Education Program (NCEP): highlights of the report of the Expert Panel on Blood Cholesterol Levels in Children and Adolescents. *Pediatrics* 1992;89(3):495-501.
24. Statistics Sweden. Socioekonomisk indelning [SEI], MIS 1982:4 SEI. [Available from: https://www.scb.se/contentassets/22544e89c6f34ce7ac2e6fefbda407ef/mis1982_4_sei.pdf/ accessed 4 november 2019.
25. Faresjo T, Rahmqvist M. Educational level is a crucial factor for good perceived health in the local community. *Scand J Public Health* 2010;38(6):605-10. doi: 10.1177/1403494810374676
26. Wennerholm C, Grip B, Johansson A, et al. Cardiovascular disease occurrence in two close but different social environments. *Int J Health Geogr* 2011;10:5. doi: 10.1186/1476-072X-10-5
27. Karlen J, Faresjo T, Ludvigsson J. Could the social environment trigger the induction of diabetes related autoantibodies in young children? *Scand J Public Health* 2012;40(2):177-82. doi: 10.1177/1403494811435491 [published Online First: 2012/02/09]
28. Ferrie JE, Shipley MJ, Davey Smith G, et al. Change in health inequalities among British civil servants: the Whitehall II study. *Journal of epidemiology and community health* 2002;56(12):922-6.
29. Steptoe A, Kunz-Ebrecht S, Owen N, et al. Socioeconomic status and stress-related biological responses over the working day. *Psychosomatic medicine* 2003;65(3):461-70. [published Online First: 2003/05/24]
30. Ruiz M, Goldblatt P, Morrison J, et al. Impact of Low Maternal Education on Early Childhood Overweight and Obesity in Europe. *Paediatr Perinat Epidemiol* 2016;30(3):274-84. doi: 10.1111/ppe.12285
31. Tabbakh T, Freeland-Graves JH. The home environment: A mediator of nutrition knowledge and diet quality in adolescents. *Appetite* 2016;105:46-52. doi: 10.1016/j.appet.2016.05.002 [published Online First: 2016/05/14]
32. Gebremariam MK, Henjum S, Hurum E, et al. Mediators of the association between parental education and breakfast consumption among adolescents : the ESSENS study. *BMC pediatrics* 2017;17(1):61. doi: 10.1186/s12887-017-0811-2 [published Online First: 2017/02/24]
33. Gebremariam MK, Lien N, Torheim LE, et al. Perceived rules and accessibility: measurement and mediating role in the association between parental education and vegetable and soft drink intake. *Nutrition journal* 2016;15(1):76. doi: 10.1186/s12937-016-0196-3 [published Online First: 2016/08/18]

For peer review only - http://bmjopen.bmj.com/site/about/guidelines.xhtml

34. Angbratt M, Ekberg J, Walter L, et al. Prediction of obesity from infancy to adolescence. *Acta Paediatr* 2011;100(9):1249-52. doi: 10.1111/j.1651-2227.2011.02326.x
35. Howe LD, Tilling K, Galobardes B, et al. Loss to follow-up in cohort studies: bias in estimates of socioeconomic inequalities. *Epidemiology* 2013;24(1):1-9. doi: 10.1097/EDE.0b013e31827623b1
36. Kagura J, Adair LS, Pisa PT, et al. Association of socioeconomic status change between infancy and adolescence, and blood pressure, in South African young adults: Birth to Twenty Cohort. *BMJ Open* 2016;6(3):e008805. doi: 10.1136/bmjopen-2015-008805
37. Krmar RT, Holtback U, Bergh A, et al. Oscillometric casual blood pressure normative standards for Swedish children using ABPM to exclude casual hypertension. *Am J Hypertens* 2015;28(4):459-68. doi: 10.1093/ajh/hpu182
38. Brettschneider AK, Ellert U, Schaffrath Rosario A. Comparison of BMI derived from parent-reported height and weight with measured values: results from the German KiGGS study. *International journal of environmental research and public health* 2012;9(2):632-47. doi: 10.3390/ijerph9020632 [published Online First: 2012/04/04]
39. Commission on Social Determinants of Health. CSDH Final Report: Closing the Gap in a Generation: Health Equity Through Action on the Social Determinant of Health. Geneva, World Health Organization, 2008.
40. OECD. In It Together: Why Less Inequality Benefits All. Paris, OECD Publishing, 2015. https://doi.org/10.1787/9789264235120-en/.
41. Juhola J, Magnussen CG, Viikari JS, et al. Tracking of serum lipid levels, blood pressure, and body mass index from childhood to adulthood: the Cardiovascular Risk in Young Finns Study. *The Journal of pediatrics* 2011;159(4):584-90. doi: 10.1016/j.jpeds.2011.03.021 [published Online First: 2011/04/26]
42. Raitakari OT, Juonala M, Kahonen M, et al. Cardiovascular risk factors in childhood and carotid artery intima-media thickness in adulthood: the Cardiovascular Risk in Young Finns Study. *Jama* 2003;290(17):2277-83. doi: 10.1001/jama.290.17.2277 [published Online First: 2003/11/06]
43. Leon DA, Vagero D, Olausson PO. Social class differences in infant mortality in Sweden: comparison with England and Wales. *BMJ (Clinical research ed)* 1992;305(6855):687-91.
44. Kunst AE, Mackenbach JP. The size of mortality differences associated with educational level in nine industrialized countries. *Am J Public Health* 1994;84(6):932-7.
45. Pickett KE, Wilkinson RG. Child wellbeing and income inequality in rich societies: ecological cross sectional study. *BMJ (Clinical research ed)* 2007;335(7629):1080. doi: 10.1136/bmj.39377.580162.55 [published Online First: 2007/11/21]
46. Lundberg O. The next step towards more equity in health in Sweden: how can we close the gap in a generation? *Scand J Public Health* 2018;46(22_suppl):19-27. doi: 10.1177/1403494818765702 [published Online First: 2018/06/05].

For peer review only

For peer review only - http://bmjopen.bmj.com/site/about/guidelines.xhtml

Table 1. Characteristics of adolescents by hypertension and overweight status.

	Normal blood pressure		Elevated blood pressure		Not overweight		Overweight	
	N	%	N	%	N	%	N	%
Total	240	83.0	49	17.0	244	84.1	46	15.9
Sex								
Boy	105	43.8	27	55.1	109	44.7	24	52.2
Girl	135	56.2	22	44.9	135	55.3	22	47.8
Ethnicity								
Swedish	213	89.9	44	91.7	218	90.5	40	88.9
Foreign ethnicity	24	10.1	4	8.3	23	9.5	5	11.1
Physical activity								
– times per week								
>2	138	67.6	31	83.8	149	72.0	21	60.0
0-2	66	32.4	6	16.2	58	28.0	14	40.0
CRD								
0-1 item	88	44.4	14	40.0	94	46.5	8	25.0
2-5 items	110	55.6	21	60.0	108	53.5	24	75.0
BMI group of adolescent								
Not OW	213	88.8	31	63.3				
OW/OB	27	11.2	18	36.7				
Income								
High	94	39.5	16	33.3	99	40.9	12	26.7
Middle	111	46.6	26	54.2	112	46.3	25	55.6
Low	33	13.9	6	12.5	31	12.8	8	17.8
Maternal education								
Tertiary	138	58.2	20	41.7	144	59.8	14	31.1
<Tertiary	99	41.8	28	58.3	97	40.2	31	68.9
SEI								
Class I	59	24.6	5	10.2	60	24.6	4	8.7

For peer review only

For peer review only - http://bmjopen.bmj.com/site/about/guidelines.xhtml

Class II-IV	131	54.6	25	51.0	130	53.3	27	58.7
Class V	50	20.8	19	38.8	54	22.1	15	32.6
City of residence								
White-collar	132	55	23	46.9	137	56.1	18	39.1
Blue-collar	108	45	26	53.1	107	43.9	28	60.9
	M	SD	M	SD	M	SD	M	SD
Age at follow-up (years)	16.02	0.90	16.12	1.04	16.02	0.92	16.18	0.94
Height (m)	1.72	0.09	1.73	0.10	1.73	0.09	1.71	0.09

BMI, Body Mass Index; *CRD*, Cardiovascular risk diet index; *OW/OB*, Overweight or obese; *SEI*, Swedish socioeconomic classification index.

For peer review only

Table 2. Univariate analysis with odds ratios for cardiovascular risk factors and areas under the ROC-curve for SES variables (n = 298).

	Elevated blood pressure		Low HDL		High LDL		Overweight	
	OR	CI	OR	CI	OR	CI	OR	CI
Age at follow-up (Z-years)	1.12	0.83,1.51	1.12	0.79,1.59	0.99	0.63,1.53	1.19	0.87,1.63
Height (Z-m)	1.24	0.91,1.64	1.41	0.85,2.33	1.26	0.85,1.88	0.82	0.59,1.14
Gender								
Girl	1.0		1.0		1.0		1.0	
Boy	1.61	0.86,3.04	2.78	1.09,7.09	1.31	0.57,3.00	1.38	0.73,2.60
Ethnicity								
Swedish	1.0		1.0		1.0		1.0	
Foreign	0.83	0.27,2.58	1.49	0.53,4.19	0.29	0.04,2.25	1.13	0.41,3.16
Physical activity								
– times/week								
>2	1.0		1.0		1.0		1.0	
0-2	0.39	0.15,1.05	0.93	0.41,2.26	0.68	0.28,1.67	1.60	0.72,3.55
CRD								
0-1 item	1.0		1.0		1.0		1.0	
2-5 items	1.12	0.49,2.61	2.41	0.95,6.15	1.41	0.63,3.16	2.80	1.06,7.41
BMI group of adolescent								
Not OW	1.0		1.0		1.0			
OW/OB	4.42	2.19,8.95	3.44	1.47,8.07	3.20	1.28,7.98		
Income								
(ROC)	0.537		0.627		0.545		0.580	
High	1.0		1.0		1.0		1.0	
Middle	1.35	0.68,2.67	0.88	0.36,2.16	1.08	0.42,2.77	1.90	0.91,3.97

For peer review only

Low	1.11	0.41,3.05	3.94	1.39,11.14	1.73	0.46,6.54	2.21	0.82,5.95
Maternal education (ROC)	0.578		0.610		0.564		0.641	
Tertiary	1.0		1.0		1.0		1.0	
<Tertiary	1.87	0.99,3.53	2.39	1.10,5.21	1.67	0.77,3.59	3.22	1.61,6.44
SEI								
(ROC)	0.623		0.540		0.573		0.602	
Class I	1.0		1.0		1.0		1.0	
Class II-IV	2.20	0.80,6.05	1.33	0.48,3.66	1.91	0.60,6.09	3.21	1.08,9.57
Class V	4.37	1.49,12.81	1.45	0.48,4.48	1.41	0.24,8.28	4.28	1.34,13.73
City of residence (ROC)	0.541		0.652		0.511		0.585	
White-collar	1.0		1.0		1.0		1.0	
Blue-collar	1.39	0.76,2.56	1.78	0.74,4.31	1.10	0.51,2.41	2.00	1.06,3.78

BMI, Body Mass Index; *CRD*, Cardiovascular risk diet index; *CI*, 95% confidence interval; *OR*, odds ratio; *OW/OB*, Overweight or obese; *ROC*, area under the receiver operating characteristic curve; *SEI*, Swedish socioeconomic classification index.

For peer review only - http://bmjopen.bmj.com/site/about/guidelines.xhtml

Table 3. Multivariate analysis with odds ratios for cardiovascular risk factors. Adjusted for SES and other explanatory variables (n = 298).

		Elevated blood pressure					
		Model 1		Model 2		Model 3	
		OR	CI	OR	CI	OR	CI
Income	High	1.00		1.00		1.00	
	Middle	0.85	0.39,1.85	0.85	0.38,1.89	0.75	0.32,1.75
	Low	0.60	0.20,1.81	0.69	0.22,2.12	0.60	0.17,1.96
Maternal education	Tertiary	1.00		1.00		1.00	
	<Tertiary	1.29	0.60,2.77	1.15	0.52,2.54	0.88	0.37,2.08
SEI	Class I	1.00		1.00		1.00	
	Class II-IV	2.21	0.77,6.34	2.25	0.78,6.52	2.09	0.70,6.27
	Class V	4.18	1.22,14.41	4.43	1,26,15.58	4.95	1.30,18.89
City of residence	White-collar	1.00		1.00		1.00	
	Blue-collar	1.12	0.58,2.14	1.18	0.61,2.30	1.05	0.52,2.13

		Low HDL (<0.40 mg/dL).					
		Model 1		Model 2		Model 3	
		OR	CI	OR	CI	OR	CI
Income	High	1.00		1.00		1.00	
	Middle	0.98	0.31,3.08	0.72	0.26,1.98	0.73	0.26,2.20
	Low	3.16	0.97,10.35	3.28	0.92,11.67	3.41	0.89,13.06
Maternal education	Tertiary	1.00		1.00		1.00	
	<Tertiary	2.56	0.99,6.62	2.29	0.86,6.09	2.00	0.73,5.50
SEI	Class I	1.00		1.00		1.00	
	Class II-IV	0.88	0.32,2.48	0.82	0.29,2.36	0.76	0.26,2.10
	Class V	0.53	0.14,2.07	0.41	0.10,1.73	0.41	0.09,1.75
City of residence	White-collar	1.00		1.00		1.00	
	Blue-collar	1.41	0.55,3.60	1.35	0.53,3.48	1.22	0.46,3.27

High LDL (>110 mg/dL)

For peer review only

		Model 1		Model 2		Model 3	
		OR	CI	OR	CI	OR	CI
Income	High	1.00		1.00		1.00	
	Middle	0.91	0.32,2.56	0.88	0.31,2.51	0.84	0.29,2.49
	Low	1.48	0.35,6.21	1.47	0.34,6.29	1.41	0.31,6.39
Maternal education	Tertiary	1.00		1.00		1.00	
	<Tertiary	1.86	0.70,4.93	1.81	0.68,4.79	1.58	0.58,4.32
SEI	Class I	1.00		1.00		1.00	
	Class II-IV	1.69	0.49,5.77	1.68	0.49,5.79	1.52	0.43,5.40
	Class V	0.92	0.10,8.56	0.97	0.10,9.06	0.90	0.09,9.54
City of residence	White-collar	1.00		1.00		1.00	
	Blue-collar	0.89	0.37,2.15	0.90	0.37,2.18	0.83	0.33,2.09

		Overweight status			
		Model 1		Model 2	
		OR	CI	OR	CI
Income	High	1.00		1.00	
	Middle	1.16	0.52,2.60	1.12	0.50,2.54
	Low	1.21	0.42,3.54	1.20	0.40,3.56
Maternal education	Tertiary	1.00		1.00	
	<Tertiary	2.63	1.20,5.78	2.39	1.08,5.30
SEI	Class I	1.00		1.00	
	Class II-IV	2.34	0.75,7.28	2.09	0.66,6.60
	Class V	1.90	0.52,6.98	1.63	0.43,6.25
City of residence	White-collar	1.00		1.00	
	Blue-collar	1.39	0.70,2.73	1.39	0.70,2.76

BMI, Body Mass Index; *CI*, 95% confidence interval; *OR*, odds ratio; *SEI*, Swedish socioeconomic classification index.

Model 1 includes all SES variables. Model 2 includes all SES and other variables excluding overweight status that reached the selection criteria in univariate analysis ($p \leq 0.05$ or OR <0.5 or >2.0 for categorical and <0.75 or >1.5 for continuous variables): physical activity for elevated blood pressure; gender and cardiovascular risk diet for low HDL; ethnicity for LDL; cardiovascular risk diet for overweight. Model 3 includes all SES, other explanatory variables and overweight status.

For peer review only - http://bmjopen.bmj.com/site/about/guidelines.xhtml

Table 4. Association between maternal education level at birth and child overweight or obesity at four ages.

Time point:	2 years	5 years	8 years	12 years
Cohort approach				
Age: mean (SD)	2.72 (0.28)	5.32 (0.30)	7.86 (0.34)	12.61 (0.87)
Sample size available	1070	1070	1070	1070
Overweight: n (%)	149 (13.9%)	146 (13.6%)	157 (14.7%)	168 (15.7%)
Obese: n (%)	19 (1.8%)	27 (2.5%)	21 (2.0%)	21 (2.0%)
Tertiary v < Tertiary education: overweight*	0.95 (0.67, 1.34)	1.42 (1.00, 2.03)	1.36 (0.96, 1.92)	1.81 (1.28, 2.55)
Tertiary v < Tertiary education: obesity*	0.41 (0.15, 1.08)	3.21 (1.29, 8.02)	1.46 (0.60, 3.56)	3.89 (1.30, 11.63)
Available sample approach				
Age: mean (SD)	2.73 (0.30)	5.34 (0.31)	7.86 (0.38)	12.40 (0.91)
Sample size available	7925	6602	2998	3337
Overweight: n (%)	1187 (15.0%)	1096 (16.6%)	446 (14.9%)	527 (15.8%)
Obese: n (%)	191 (2.4%)	273 (4.1%)	71 (2.4%)	75 (2.2%)
Tertiary v < Tertiary education: overweight*	1.18 (1.03, 1.34)	1.30 (1.13, 1.50)	1.16 (0.94, 1.42)	1.70 (1.39, 2.06)
Tertiary v < Tertiary education: obesity*	1.56 (1.12, 2.17)	1.52 (1.15, 1.99)	2.28 (1.31, 3.95)	2.16 (1.29, 3.61)

*Odds ratio (95% confidence interval)

Note: The cohort approach utilises only participants with all necessary data recorded at all four ages whereas the available sample approach uses any participant with all necessary data recorded on at least one age.

For peer review only

Supplementary table 1: Comparison of original ABIS cohort, invited subsample, participants and non-participants in the cardiovascular risk study.

	Original cohort		Invited to subsample		Participants		Non- participants		p[1]	p[2]	p-[3]
	N=16349		N=578		N=298		N=240				
	Mean/N	SD/%	Mean/N	SD/%	Mean/N	SD/%	Mean/N	SD/%			
Birthweight (g)	3578.6	560.8	3552.6	553.6	3554.0	574.7	3551.1	530.8	0.27	0.46	0.95
Gender									0.11	0.05	0.23
Boys	8473	51.9 %	279	48.5	136	46.1	143	46.1 %			
Girls	7866	48.1 %	296	51.5	159	53.9	137	48.9 %			
Ethnicity									0.09	0.50	0.01
Swedish	14235	89.3 %	492	87.1 %	266	90.5 %	226	83.4 %			
Other	1711	10.7 %	73	12.9 %	28	9.5 %	45	16.6 %			
Father ow/ob[a]									0.19	0.54	0.51
Not ow/ob	5384	54.0 %	254	56.4 %	146	57.3 %	108	57.3 %			
Overweight	4003	40.1 %	164	36.4 %	94	36.9 %	70	35.9 %			
Obese	586	5.9 %	32	7.1 %	15	5.9 %	17	8.7 %			
Mother ow/ob[a]									0.95	0.22	0.01
Not ow/ob	7310	70.0 %	328	69.6 %	191	72.9 %	137	65.6 %			

For peer review only

Overweight	2372	22.7 %	107	22.7 %	59	22.5 %	48	23.0 %			
Obese	759	7.3 %	36	7.6 %	12	4.6 %	24	11.5 %			
Overweight at 2 yrs									0.46	0.75	0.71
Not ow/ob	6890	85.0 %	307	83.7 %	187	84.2 %	120	82.8 %			
Ow/ob	1217	15.0 %	60	16.3 %	35	15.8 %	25	17.2 %			
Overweight at 5 yrs									0.35	<0.01	<0.01
Not ow/ob	5624	83.3 %	287	85.2 %	187	90.3 %	100	76.9 %			
Ow/ob	1125	16.7 %	50	14.8 %	20	9.7 %	30	23.1 %			
Overweight at 8 yrs									0.76	0.51	0.10
Not ow/ob	2566	85.1 %	2566	85.1 %	139	86.9 %	50	78.1 %			
Ow/ob	450	14.9 %	450	14.9 %	21	13.1 %	14	21.9 %			
Overweight at 12 yrs									0.63	0.20	0.08
Not ow/ob	2836	84.2 %	197	85.3 %	155	87.6 %	42	77.8 %			
Ow/ob	534	15.8 %	34	14.7 %	22	12.4 %	12	22,2 %			
Maternal education									< 0.01	< 0.01	<0.01
High	5062	31.8 %	233	41.3 %	160	54.4 %	73	27.0 %			
Low	10882	68.2 %	331	58.7 %	134	45.6 %	197	73.0 %	197	73.0 %	
Income									< 0.01	< 0.01	< 0.01
High	3239	20.0 %	153	26.7 %	114	38.6 %	39	14.0 %			
Middle	9718	60.0 %	301	52.5 %	141	47.8 %	160	57.6 %			
Low	3239	20.0 %	119	20.8 %	40	13.6 %	79	28.4 %			

For peer review only

p-value[1]= Invited to follow-up vs original cohort, T-test or Chi2 as appropriate
p-value[2]= Participants in follow-up vs original cohort, T-test or Chi2 as appropriate
p-value[3]=Participants vs non-participants in follow-up, T-test or Chi2 as appropriate
[a] Parental height and weight self-reported at child age 1 year
T-test or Chi2 as appropriate

For peer review only

Reporting checklist for cohort study.

Based on the STROBE cohort guidelines.

Instructions to authors

Complete this checklist by entering the page numbers from your manuscript where readers will find each of the items listed below.

Your article may not currently address all the items on the checklist. Please modify your text to include the missing information. If you are certain that an item does not apply, please write "n/a" and provide a short explanation.

Upload your completed checklist as an extra file when you submit to a journal.

In your methods section, say that you used the STROBE cohort reporting guidelines, and cite them as:

von Elm E, Altman DG, Egger M, Pocock SJ, Gotzsche PC, Vandenbroucke JP. The Strengthening the Reporting of Observational Studies in Epidemiology (STROBE) Statement: guidelines for reporting observational studies.

		Reporting Item	Page Number
Title	#1a	Indicate the study’s design with a commonly used term in the title or the abstract	1
Abstract	#1b	Provide in the abstract an informative and balanced summary of what was done and what was found	2
Background / rationale	#2	Explain the scientific background and rationale for the investigation being reported	4
Objectives	#3	State specific objectives, including any prespecified hypotheses	5
Study design	#4	Present key elements of study design early in the paper	6
Setting	#5	Describe the setting, locations, and relevant dates, including periods of recruitment, exposure, follow-up, and data collection	6-7

For peer review only

Eligibility criteria	#6a	Give the eligibility criteria, and the sources and methods of selection of participants. Describe methods of follow-up.	6-7
	#6b	For matched studies, give matching criteria and number of exposed and unexposed	n/a
Variables	#7	Clearly define all outcomes, exposures, predictors, potential confounders, and effect modifiers. Give diagnostic criteria, if applicable	7-10
Data sources / measurement	#8	For each variable of interest give sources of data and details of methods of assessment (measurement). Describe comparability of assessment methods if there is more than one group. Give information separately for for exposed and unexposed groups if applicable.	7-10
Bias	#9	Describe any efforts to address potential sources of bias	10
Study size	#10	Explain how the study size was arrived at	5
Quantitative variables	#11	Explain how quantitative variables were handled in the analyses. If applicable, describe which groupings were chosen, and why	7-10
Statistical methods	#12a	Describe all statistical methods, including those used to control for confounding	10
	#12b	Describe any methods used to examine subgroups and interactions	N/A
	#12c	Explain how missing data were addressed	10
	#12d	If applicable, explain how loss to follow-up was addressed	6-7
	#12e	Describe any sensitivity analyses	N/A
Participants	#13a	Report numbers of individuals at each stage of study—eg numbers potentially eligible, examined for eligibility, confirmed eligible, included in the study, completing follow-up, and analysed. Give information separately for for exposed and unexposed groups if applicable.	12-13
	#13b	Give reasons for non-participation at each stage	7
	#13c	Consider use of a flow diagram	N/A

For peer review only

Descriptive data	#14a	Give characteristics of study participants (eg demographic, clinical, social) and information on exposures and potential confounders. Give information separately for exposed and unexposed groups if applicable.	Table 1
	#14b	Indicate number of participants with missing data for each variable of interest	Table 1
	#14c	Summarise follow-up time (eg, average and total amount)	6
Outcome data	#15	Report numbers of outcome events or summary measures over time. Give information separately for exposed and unexposed groups if applicable.	12-13
Main results	#16a	Give unadjusted estimates and, if applicable, confounder-adjusted estimates and their precision (eg, 95% confidence interval). Make clear which confounders were adjusted for and why they were included	10-13
	#16b	Report category boundaries when continuous variables were categorized	8-10
	#16c	If relevant, consider translating estimates of relative risk into absolute risk for a meaningful time period	N/A
Other analyses	#17	Report other analyses done—e.g., analyses of subgroups and interactions, and sensitivity analyses	N/A
Key results	#18	Summarise key results with reference to study objectives	14
Limitations	#19	Discuss limitations of the study, taking into account sources of potential bias or imprecision. Discuss both direction and magnitude of any potential bias.	15-16
Interpretation	#20	Give a cautious overall interpretation considering objectives, limitations, multiplicity of analyses, results from similar studies, and other relevant evidence.	17-18
Generalisability	#21	Discuss the generalisability (external validity) of the study results	15-16
Funding	#22	Give the source of funding and the role of the funders for the present study and, if applicable, for the original study on which the present article is based	18

For peer review only

The STROBE checklist is distributed under the terms of the Creative Commons Attribution License CC-BY. This checklist was completed on 22. March 2019 using https://www.goodreports.org/, a tool made by the EQUATOR Network in collaboration with Penelope.ai

For peer review only

BMJ Open

Inequalities in Cardiovascular risks among Swedish adolescents – ABIS a prospective cohort study

Journal:	*BMJ Open*
Manuscript ID	bmjopen-2019-030613.R3
Article Type:	Original research
Date Submitted by the Author:	08-Jan-2020
Complete List of Authors:	Andersson White, Pär; Linköping University, Dept. of Medicine and Health; Linköping University Hospital, Crown Princess Victoria Children´s Hospital<br>Ludvigsson, Johnny; Linkopings universitet, Div of Pediatrics, Dept of Clin Exp Medicin, Medical Faculty<br>Jones, Michael; Macquarie University<br>Faresjo, Tomas; Linköpings universitet Hälsouniversitetet
**Primary Subject Heading**:	Paediatrics
Secondary Subject Heading:	Epidemiology, Cardiovascular medicine, Public health
Keywords:	PUBLIC HEALTH, EPIDEMIOLOGY, Cardiac Epidemiology < CARDIOLOGY, Paediatric endocrinology < DIABETES & ENDOCRINOLOGY, SOCIAL MEDICINE

SCHOLARONE™
Manuscripts

I, the Submitting Author has the right to grant and does grant on behalf of all authors of the Work (as defined in the below author licence), an exclusive licence and/or a non-exclusive licence for contributions from authors who are: i) UK Crown employees; ii) where BMJ has agreed a CC-BY licence shall apply, and/or iii) in accordance with the terms applicable for US Federal Government officers or employees acting as part of their official duties; on a worldwide, perpetual, irrevocable, royalty-free basis to BMJ Publishing Group Ltd ("BMJ") its licensees and where the relevant Journal is co-owned by BMJ to the co-owners of the Journal, to publish the Work in this journal and any other BMJ products and to exploit all rights, as set out in our licence.

The Submitting Author accepts and understands that any supply made under these terms is made by BMJ to the Submitting Author unless you are acting as an employee on behalf of your employer or a postgraduate student of an affiliated institution which is paying any applicable article publishing charge ("APC") for Open Access articles. Where the Submitting Author wishes to make the Work available on an Open Access basis (and intends to pay the relevant APC), the terms of reuse of such Open Access shall be governed by a Creative Commons licence – details of these licences and which Creative Commons licence will apply to this Work are set out in our licence referred to above.

Other than as permitted in any relevant BMJ Author's Self Archiving Policies, I confirm this Work has not been accepted for publication elsewhere, is not being considered for publication elsewhere and does not duplicate material already published. I confirm all authors consent to publication of this Work and authorise the granting of this licence.

For peer review only

Inequalities in Cardiovascular risks among Swedish adolescents – ABIS a prospective cohort study

Pär Andersson White[1, 2], Johnny Ludvigsson[2], Michael P. Jones[3], Tomas Faresjö[1]

[1]Dept. of Medicine and Health, Div. of Community Medicine, Linköping University, Sweden.

[2]Crown Princess Victoria Children´s Hospital and Div. of Pediatrics, Dept. of Clinical and Experimental Medicine, Linköping University, Sweden.

[3]Psychology Department, Macquarie University, North Ryde, NSW, 2109, Australia.

Corresponding author: Pär Andersson White, C/O T Faresjo, Dept. of Medicine and Health, Div. of Community Medicine, Linköping University, SE-581 83 Linköping Sweden, +4610-1030000, par.andersson.white@liu.se

Keywords: Socioeconomic status, Inequalities, Adolescent Overweight, Childhood overweight, Childhood obesity, Blood Pressure, Dyslipidemia, High-Density Lipoproteins, Low-Density Lipoproteins, ABIS

For peer review only - http://bmjopen.bmj.com/site/about/guidelines.xhtml

Abstract

OBJECTIVES: To investigate if Socioeconomic Status (SES) is predictive of cardiovascular risk factors among Swedish adolescents. Identify the most important SES variable for the development of each cardiovascular risk factor. Investigate at what age SES inequality in overweight and obesity occurs.

DESIGN: Longitudinal follow-up of a prospective birth cohort.

SETTING: All Babies in Southeast Sweden (ABIS) study includes data from children born between October 1997 and October 1999 in five counties of south east Sweden.

PARTICIPANTS: A regional ABIS-study subsample from three major cities of the region n=298 adolescents aged 16-18 years, and prospective data from the whole ABIS cohort for overweight and obesity status at the ages 2, 5, 8 and 12 years (n= 2998-7925).

OUTCOME MEASURES: Blood pressure above the hypertension limit, overweight/obesity according to the International Obesity Task Force (IOTF) definition, low high-density lipoproteins (HDL) or borderline-high low-density lipoproteins (LDL) according to National Cholesterol Education Program (NCEP) expert panel on cholesterol levels in children.

RESULTS: For three out of four cardiovascular risk outcomes (elevated blood pressure, low HDL and overweight/obesity) there were increased risk in one or more of the low SES groups ($p < 0.05$). The best predictor was parental occupational class (SEI) for elevated blood pressure (Area under the ROC curve 0.623), maternal educational level for overweight (Area under the ROC curve 0.641) and blue-collar city of residence for low HDL (Area under the ROC curve 0.641). SES related differences in overweight/obesity were found at age 2, 5 and 12 and for obesity at age 2, 5, 8, and 12 years (all $p<0.05$).

For peer review only - http://bmjopen.bmj.com/site/about/guidelines.xhtml

CONCLUSIONS: Even in a welfare state like Sweden, SES inequalities in cardiovascular risks are evident already in childhood and adolescence. Intervention programs to reduce cardiovascular risk based on social inequality should start early in life.

Strengths and limitations of this study:

- Data derives from a large prospective cohort representative for the general population.
- A standardized procedure for collection of clinical data.
- The Swedish National registers give us valid and reliable data on household income.
- The limited sample size of the follow–up group gives some restraints.

For peer review only

For peer review only - http://bmjopen.bmj.com/site/about/guidelines.xhtml

Introduction

Cardiovascular disease in adults has been shown to be one of the disease groups most correlated with socioeconomic inequalities in the world [1], and this has also been evident in Swedish adults [2]. Longitudinal studies have shown that risk factors for cardiovascular disease develop in childhood and that early signs of cardiovascular disease risks are visible in early adulthood [3-5]. The risk factors associated with cardiovascular disease include blood pressure, dyslipidemia and overweight. Of these risk factors, overweight and obesity are the ones that progress most consistently from childhood to adulthood [6]. Adolescent overweight has been shown to be a stronger predictor of cardiovascular mortality in adulthood than adult overweight [7].

Recent studies report lower scores than ideal in a range of cardiovascular health factors (blood pressure, cholesterol and glucose level below the American heart association's cut-offs) in European adolescents with low maternal education [8]. In Korea high income was protective against cardiovascular disease risk factors and in Finland low socioeconomic status (SES) measured by occupation of the father was associated with higher low-density lipoprotein (LDL) levels in boys and higher systolic blood pressure and higher percentage of smokers [9 10]. However, longitudinal data on the relationships between childhood SES and adolescent's cardiovascular health is still scarce.

An increase in physical activity has been shown to reduce the risk of being overweight and obese as a youth in Sweden and other European countries [11]. Studying the effect of diet on development of overweight and obesity in children and adolescents has yielded no clear conclusions on whether any specific foods are important for the development of obesity [12]. However, there is some evidence for intake of certain foods and development of adult

For peer review only - http://bmjopen.bmj.com/site/about/guidelines.xhtml

cardiovascular disease. The INTERHEART study found a positive association between intake of meat, salty foods, fried foods and development of cardiovascular disease and a negative association between intake of fruits and vegetables and cardiovascular disease risk [13].

Sweden currently ranks as 9th in the Organization for Economic Co-operation and Development (OECD) list of economic equality [14]. It has a comprehensive public health system with a number of interventions aimed at reducing social inequalities. These include universal and public financed healthcare equally accessible for all. The healthcare system provides free antenatal consulting and check-ups at Child Health Care Centers which covers advice on nutrition and health from professional nurses for all children at regular intervals up to school age. Other interventions include school-health check-ups and mandatory physical activity in schools and free school lunches for all children. Other support for families with children includes paid maternity leave for 480 days and heavily subsidized daycare [15 16].

Aims

The aims of this study were to determine whether SES is predictive of cardiovascular risk factors in Swedish adolescents and to identify which socioeconomic factors that were most important for the development of each risk factor. Further, using longitudinal data we aimed to shed light on at what ages preventive measures might be important for reducing SES differences in overweight and obesity.

Material and Methods

Study population

For peer review only

The All Babies in Southeast Sweden (ABIS) study is a prospective population-based cohort study, initially including 17 055 children born between October 1st, 1997 and October 1st 1999 in southeast Sweden in the counties of Östergötland, Jönköping, Kronoberg, Kalmar, and Blekinge. The ABIS region is inhabited by approx. 1.25 million out of Sweden's 10 million inhabitants[17]. The region incorporates three of the biggest cities of the country, Linköping (5th), Norrköping (9th) and Jönköping (10th), but also large rural areas, both farmland and forest areas. In terms of income inequality, the counties of southeast Sweden are placed close to the average of the country between the most unequal areas (the capital Stockholm) and the northern areas with the lowest income inequality[18]. This cohort of children has been followed from birth and are still being followed with respect to biological samples, questionnaires and register data. The initial aim of the ABIS study was to analyse the development of immune-mediated diseases including autoimmune diseases, type 1 and type 2 diabetes as well as cardiovascular disease, and the effect of different environmental and genetic factors.

We undertook a follow-up of a regional subsample of ABIS children and also made a questionnaire-based longitudinal analysis for the whole cohort. Inclusion criteria for the regional subsample was residence in one of the three major cities of Östergötland County. Out of n=578 children invited n=298 (51.6%) participated. The main reasons for non-participation among the invited sample were practical i.e. difficulties to arrange health check-ups for our research nurse at some schools that lacked school-nurse facilities. The study participants filled out a questionnaire about their lifestyle, diet and physical activity. A specially trained research nurse undertook all data collection including blood pressure measurement, questionnaire and collection of blood samples and measurement of anthropometric data.

For peer review only - http://bmjopen.bmj.com/site/about/guidelines.xhtml

Blood Pressure

Systolic and diastolic blood pressure was measured manually by the research nurse at the participant's school nursing offices. Study participants rested on a bed at least 5 minutes before measurement, two consecutive measurements of blood pressure were then taken in the sitting position and the mean value was calculated. Blood pressures were grouped as hyper- or normotensive according to the guidelines described in the fourth report on the diagnosis, evaluation, and treatment of high blood pressure in children and adolescents [19]. These guidelines define hypertension as an average systolic and/or diastolic blood pressure ≥95th percentile for gender, age and height percentile according to the Center of Disease Control and Preventions growth charts on three or more occasions [20].

Overweight/obesity

The participating adolescents' height (cm) and weight (kg) were measured by the research nurse. Data on weight and height were missing for 8 participants. Body mass index (BMI) was calculated as BMI = weight(kg)/height(m)^2 and dichotomized as overweight/obese or not, using the International obesity task force latest age and gender specific cut-offs defined by Cole et al [21 22]. The same procedure was then used for parental-reported height and weight of the child available from previous ABIS follow-ups at age 2, 5, 8 and 12 years.

Dyslipidemia

Blood samples for blood lipid analysis were collected from 242 (81.2 %) individuals and analyzed for plasma levels of LDL and HDL at the Department of clinical chemistry, Linköping University Hospital, according to accredited procedures. One sample was missing data on LDL after analysis. Test results were converted from SI units to mg/dL. Results were then

For peer review only - http://bmjopen.bmj.com/site/about/guidelines.xhtml

grouped as normal or low HDL cholesterol in accordance with the cut-off for low HDL cholesterol (<40 mg/dL) set by the Expert panel on integrated guidelines for cardiovascular health and risk reduction in children, which is based on the 10th percentile found by the National cholesterol educational program (NCEP) [6 23]. For LDL only 8 (3.3%) out of 241 had a level above the limit of high LDL (the 95th percentile in NCEP). As this number was insufficient for statistical analyses the cut-off of borderline high LDL (>110 mg/dL, the 75th percentile in NCEP) was chosen as indicator of increased LDL.

Socioeconomic variables

A five grade Socioeconomic Class (SEI) variable was constructed based on parents occupation and the occupational groups used by Statistics Sweden in their Swedish socioeconomic classification index[24]. The definitions for each class was; Class I Professionals (SEI 54-60), Class II Intermediate non-manual employees (SEI 44-46), Class III Assistant non-manual employees (SEI 33-36), Class IV Skilled workers (SEI 21-22) and Class V Unskilled workers (SEI 11-12). Occupations in SEI 76-87 which includes non-professional self-employees including farmers were given a Class I-V based on their educational attainment. The adolescents were classified according to the highest occupational class of either of their parents. Mother's educational level was derived from the ABIS birth questionnaire and grouped as primary/secondary education or post-secondary education/university. Family disposable income was derived from Statistics Sweden for the year 2012 and grouped into three groups; low income representing the bottom 20 % of household disposable incomes in the original cohort (available for n=16196), middle income representing the middle 20 to 80 percentiles of income and top income representing the top 20 percent of income.

For peer review only - http://bmjopen.bmj.com/site/about/guidelines.xhtml

The three included cities in this study represent different social history and different socioeconomic environments [25]. The participants from the city of Motala and Norrköping, are towns with a mainly industrial history, and were merged in the analysis and labelled as blue-collar cities. The city of Linköping has a history as a political and ecclesiastical administrative centre of the county, was labelled as white-collar city. Today this city has a workforce of mainly civil servants including high tech industries and one of Sweden's major universities. Previous studies of these cities has exhibit marked public health differences, including higher mortality in cardiovascular disease among adults in the blue-collar cities and difference in life-expectancy [26 27].

Covariates

Age was calculated using the date of answering the questionnaire and the participants birth date. Height was measured at follow-up by a research nurse. Ethnicity was based on the question "Are you (the mother) born in Sweden" and "Is the father born in Sweden". The adolescents were then grouped as having foreign ethnicity if one of the parents was born outside of Sweden. A cardiovascular risk diet index (CRD) was constructed based on the dietary components most associated with myocardial infarction in the INTERHEART study [13]. These factors were: intake of salty foods one time per day or more, intake of deep-fried foods three times or more per week, intake of meat two or more times per day, intake of fruit less than once per day and intake of vegetables less than once per day. This score which has a range of 0 to 5 was then dichotomized into two groups Low CRD (0-1 items n=101) and High CRD (2 to 5 items n=138). Degree of physical activity was measured by the question "How many times per week do you do physical activity at least 30 min that is

For peer review only - http://bmjopen.bmj.com/site/about/guidelines.xhtml

strenuous enough to make you sweat". The answers were then grouped into two groups; 0-2 times a week, >2 times a week.

Patient and Public Involvement

Patients and public were not involved in the design of this study. The results will be disseminated to the study participants through the ABIS study homepage https://www.abis-studien.se/.

Statistical analysis

Data were analyzed using bivariate logistic regression for each cardiovascular risk factor. As analysis of missing values showed significant differences due to SES, adolescents with low maternal educational level had a significantly higher number of missing values in questionnaire data, multiple imputation was used for missing values to reduce this potential bias. Multiple imputation was employed as implemented in the SPSS statistics program. A Markov Chain Monte Carlo was employed with predictive models selected according to the measurement scale of each dependent variable specified in five imputation samples. A univariate logistic regression was then analyzed for each independent variable (SES variables and covariates) and Odds Ratios (OR) with 95% confidence interval for each dependent variable (elevated blood pressure, low-HDL, high-LDL and overweight/obesity at last follow-up) were calculated. For further multivariate analysis all four SES variables (maternal education, income, SEI, white/blue collar city) were included on a priori basis while other independent variables were chosen based on reaching statistical significance $p \leq 0.05$ or an effect size of OR <0.5 or >2.0 for categorical variables. For continuous variables; height and

For peer review only

age, was standardized and the OR for 1 standard deviation increase of <0.75 or >1.5 was used as selection criteria. This approach was adopted due to limited sample size and, in this context, effect size being as important from a public health perspective as statistical significance.

The degree of discrimination afforded by each socioeconomic variable was quantified by the area under the receiver operating characteristic (ROC) curve defined by taking the logistic model predicted probability of adverse health state as the test measure and the actual state as an imperfect reference standard. Values >0.5 indicate some discrimination with values close to 1.0 indicating excellent discrimination.

A hierarchical approach was adopted in which SES variables were first adjusted for other SES variables, then for other independent variables reaching the selection criteria and finally in three of the four outcomes (elevated blood pressure, low LDL and high LDL) for adolescent's overweight status.

Bivariate logistic regression was also used in the final analysis on the relationship between overweight/obesity and maternal educational level at four different time points. The first analysis used a cohort approach and included only subjects with complete data from all follow-ups (n = 1070). The second analysis used an available sample approach and included all subjects that had data for any specific age (n=2995-7925).

Statistical analysis was performed using Statistical Package for the Social Sciences v 23 (SPSS, Chicago, IL, USA).

Ethical considerations

For peer review only

For peer review only - http://bmjopen.bmj.com/site/about/guidelines.xhtml

The ABIS-study has been approved by the Research Ethics committees of the Faculty of Health Science at Linköping University and Lund university. Dnr 03-092, Dnr 99-227 and Dnr 99-321. All participants in the follow-up gave their informed consent as well as their caregivers consent for those below 18 years of age.

Results

The participants in our follow-up had a mean age of 16.05 (SD 0.9) years (n=136 males, n=162 females). They were distributed between the cities of Linköping n=160 (54%), Norrköping n=91 (30%) and Motala n=47 (16%). 24 (9.7 %) out of 248 were categorized as having foreign ethnicity. The invited subsample was similar to the original ABIS cohort in respect to a number of variables (birthweight, ethnicity, parental overweight and obesity, child overweight at age 2, 8 and 12 years). However, comparison of this ABIS follow-up with the original ABIS cohort showed a higher proportion of mothers with high education and families with high income and a lower proportion with overweight at 5 years. There was also a slight difference (p=0.052) in gender composition with boys being underrepresented in the subsample. Non-participation analysis also showed underrepresentation of children with foreign ethnicity, low maternal education and low household income (see supplementary table 1).

The total number of participants that were classified as having elevated blood pressure was 49/290 (16.9 %), see table 1. This was based on 34/290 participants (11.7%) with systolic blood pressures above the limit for hypertension and 17/290 (5.9 %) with diastolic blood pressure above the limit. Two individuals were above both systolic and diastolic hypertension limits. An HDL level below 40 mg/dL was found in 30 (12.4 %) out of 242

For peer review only - http://bmjopen.bmj.com/site/about/guidelines.xhtml

samples. For LDL 25/241 (10.4 %) were above the cut-off for borderline-high value (110 mg/dL).

Elevated blood pressure

All SES variables were associated with increased odds ratios (OR) for elevated blood pressure in the lowest SES groups compared with the highest, see table 2. Out of the four SES variables, the parental occupational class variable SEI had the highest area under the ROC curve, 0.623 compared to 0.537-0.578 for the other SES variables. The lowest social class had an OR of 4.37 (95% confidence interval 1.49, 12.81) compared to the highest class.

After adjustments for all other SES variables, physical activity and overweight status the lowest SEI class had an OR for elevated blood pressure of 4.95 (95% confidence interval 1.30, 18.89), see table 3.

Dyslipidemia

All indicators of low SES showed an increased odd ratio of having low HDL. Out of the four variables, white/blue-collar city was associated with the highest area under the ROC curve, 0.652 compared to 0.540-0.627 for the other SES variables. Blue-collar city was associated with an OR of 1.78 (95% confidence interval 0.74, 4.31) of low HDL. Low maternal education and low income were both associated with significantly increased risks of low HDL, low maternal education with an OR of 2.39 (95% confidence interval 1.10, 5.21) and low income with an OR of 3.94 (95% confidence interval 1.39, 11.14) for having low HDL.

After adjustment for all other SES variables, gender, CRD and overweight the OR for blue-collar city was reduced to 1.22 (95% confidence interval 0.46, 3.27).

For peer review only - http://bmjopen.bmj.com/site/about/guidelines.xhtml

For peer review only

For LDL the difference between low and high SES groups were small, no SES variable reached an OR above 2.0, and no variable reached statistical significance $p \leq 0.05$. The area under the ROC curve ranged from 0.573 for SEI to 0.511 for white/blue-collar city.

Overweight/obesity

In the regional subsample 46/290 (15.8 %) were overweight or obese, with 7 (2.4 %) obese. All four measures of low SES had an increased odds ratio of being overweight or obese. The best discriminator measured as area under the ROC curve for a single SES variable was 0.641 for maternal education. The only other variable that met the criteria for being included in the multivariate analysis was diet according to CRD.

After adjustment for all other SES variables and CRD maternal education had an odds ratio of 2.39 (95% confidence interval 1.08, 5.30) for being overweight.

Overweight and obesity inequality during childhood

Overweight and obesity differences related to maternal education were measured longitudinally at four different time-points. In the analysis on subjects with complete data (cohort approach) the children to low educated mothers´ had significantly increased odds of being overweight/obese at age 12, but not at age 2, 5 and 8. The analysis based on all available data (available sample approach) showed significant differences at age 2, 5 and 12 but not at age 8. For obesity there were significant differences at age 5 and 12 in the cohort approach, while the available sample approach showed significant differences at all ages, 2, 5, 8 and 12 years, see table 4.

For peer review only - http://bmjopen.bmj.com/site/about/guidelines.xhtml

Discussion

There was significantly increased risk of three out of four cardiovascular risk factors due to low SES in Swedish adolescents. Parental occupational class according to SEI was the most important factor for risk of elevated blood pressure. Although overweight was important for elevated blood pressure the independent importance of parental occupational class was further strengthened as it remained after adjustment for all other SES factors, physical activity and overweight. For other outcomes we found maternal education to be the best discriminator for overweight and white/blue collar city for low HDL (<40 mg/dL). Our results further show that socioeconomic differences in overweight and obesity are not only of importance in adolescents but also early childhood. This finding yields implications for preventive measures and suggests that these measures should start early in life.

In the Whitehall studies, occupational class and blood pressure were found to be closely associated in adults [28]. One finding of the Whitehall II study is that workers with low occupational grade have increased blood pressure during the working day, but no difference in resting blood pressure [29]. As blood pressure in this study was measured while the adolescents' were attending school it is possible that it represents the same kind of increase in response to school tasks in low SES adolescents as that seen during the work day in adult workers.

The impact of maternal education on child overweight has been shown in large multi cohort studies [30]. In addition to being a marker for SES like occupation and income the impact of maternal education could involve differences in knowledge. There is evidence that adolescents with mothers with good nutritional knowledge have a healthier diet [31]. High maternal education has also been associated with a higher degree of breakfast eating in

For peer review only - http://bmjopen.bmj.com/site/about/guidelines.xhtml

adolescents [32]. Further, low parental education has been associated with increased access to sugar sweetened drinks and low vegetable intake [33]. These factors could be part of the explanation of why maternal educational level is more important than other SES factors for overweight status in adolescents.

White/blue-collar city was the best discriminator of low HDL status. After adjustment for other SES variables the odds ratio for blue-collar cities was reduced to OR 1.35 (0.53, 3.48), adjusting for diet and overweight further decreased the OR to 1.22 (0.46, 3.27) indicating that most of the risk associated with blue-collar residence is due to differences in SES, diet and overweight. Thus, our results are in line with previous studies [25].

The main limitation of this study is the limited number of participants of the follow-up part (n=298). Even though the sample size of study is somewhat restrained we managed to capture SES related significant differences in cardiovascular risks. Our sample is quite representative concerning overweight status compared to the previous larger samples in ABIS and to other Swedish studies [34]. Although the number of low income families were similar in our invited subsample compared to the original cohort there was an overrepresentation of high maternal education and high income families, this most likely reflects the fact that the subsample is from a more urban population than the rest of the ABIS region and must be taken into consideration when interpreting the result. Among the invited, the number of adolescents with high maternal education at birth were higher among the participants of the study than in the non-participant group and the same was true for high income families. This is a common finding in longitudinal studies, but as previous studies have shown, the effect of this underrepresentation in low SES groups, if anything, is to underestimate inequality slightly in later follow-ups and have little effect on

For peer review only

For peer review only - http://bmjopen.bmj.com/site/about/guidelines.xhtml

the qualitative conclusion on inequality [35]. The number of adolescents with elevated blood pressure was quite high (16.9%) and as blood pressure was measured only at one visit to school by the research nurse some of the adolescents might have had White-coat rather than true hypertension [19]. However, other studies have shown similarly high values in other populations [36]. A recent study in a Swedish cohort also found higher average systolic blood pressure in the age group 14-16 years than was found in the fourth report on the diagnosis, evaluation, and treatment of high blood pressure in children and adolescents [37]. We could not rule out that White-coat hypertension might be related to socioeconomic factors but have no evidence of that.

The fact that the longitudinal data was based on parental-reported height and weight might affect the accuracy of our results. A German study found that parental reported data tends to overestimate the prevalence of overweight in young children and underestimate it in older children, however the same study found the impact of SES on this error to be small.[38] Thus, although the exact prevalence of overweight status in our study might differ slightly from the true prevalence the relative difference between SES groups should not.

Different socioeconomic variables can have different effects on health. It is a strength that we investigated not one but four dimensions of SES inequality. These dimensions seem to be of varying importance for cardiovascular risk outcomes. Thus our study is an example of the many layers of the social determinants of health that give rise to an individual's risk of disease [39]. Although income, maternal education and occupational status are correlated, it is not surprising that the strongest discriminators for cardiovascular risk factors are occupational class, blue collar city and educational level rather than income in this Swedish cohort, as income inequality still is low compared to most other countries [40].

For peer review only

The implication of adolescent SES differences on adult life cardiovascular health inequalities could be important. Longitudinal data from Finland have shown that cardiovascular risk factors such as elevated blood pressure, serum lipids and obesity tracks to adulthood, increasing the risk of adult abnormalities [41]. Data from the same cohort also shows that adolescent blood pressure and LDL cholesterol are risk factors of adult carotid intima media thickening independent of adult risk factors [42]. A general awareness in primary care is important of the potential risk of elevated blood pressure and other cardiovascular risks in low SES adolescents.

Sweden has for a long time been considered one of the more egalitarian countries in the world and has often been used for comparison in studies on health inequalities as a good example [43 44]. Yet during the last two decades this has started to change with economic inequality increasing faster than in other OECD countries and Sweden has gone from a top position in equality to now being the 9th country in income equality out of the 36 countries in OECD [14]. Previous studies have found that for health problems with higher prevalence in low SES groups, like cardiovascular disease in adults, both the over-all prevalence and absolute inequality between high and low SES groups increases with larger economic inequality [1 45]. Our finding that cardiovascular risks in children and adolescents have a SES gradient also in Sweden could have serious public health implication for the future, as the rapid rise of economic inequality might increase absolute inequalities and prevalence of cardiovascular risk factors in children and adolescents. Concerns for health inequalities have increased in recent years spurring the Swedish government to start a Commission for health equality with the aim of finding ways to decrease these inequalities [46].

Conclusion

For peer review only - http://bmjopen.bmj.com/site/about/guidelines.xhtml

Despite an extensive welfare system and public health interventions in Sweden, cardiovascular health inequality is evident among Swedish youths and children. Preventive health services and clinicians must be aware of the increased risk of cardiovascular risk factors in low SES children and adolescents and improve prevention programs in this group. Policies aimed at decreasing the evident inequalities in cardiovascular disease in adults should start early and include interventions already in childhood and adolescence.

For peer review only

For peer review only - http://bmjopen.bmj.com/site/about/guidelines.xhtml

Funding

The ABIS-study has been supported by Swedish Research Council (K2005-72X-11242-11A and K2008-69X-20826-01-4) and the Swedish Child Diabetes Foundation (Barndiabetesfonden), JDRF Wallenberg Foundation (K 98-99D-12813-01A), Medical Research Council of Southeast Sweden (FORSS) and the Swedish Council for Working Life and Social Research (FAS2004–1775) and Östgöta Brandstodsbolag. The Research and PhD studies Committee (FUN), Linköping University, Sweden (LiU-).

Competing interests

None declared.

Data sharing statement:

Data from the ABIS registry, including study protocols, report forms and consents may be obtained through requests to the PI and project coordinator Senior professor Johnny Ludvigsson email: Johnny.Ludvigsson@liu.se

Author Contributions

TF, JL and PAW were responsible for the study concept and design. JL is the principal investigator for ABIS. PAW did the statistical analysis with advice from MJ. All authors were responsible for analysis and interpretation of data. PAW drafted the manuscript and TF, JL and MJ undertook critical revision of the manuscript. PAW has full access to all of the data in the study, takes responsibility for the integrity of the data and accuracy of the data analysis and had the final responsibility to submit for publication.

For peer review only - http://bmjopen.bmj.com/site/about/guidelines.xhtml

References

1. Wilkinson RG, Pickett KE. Income inequality and socioeconomic gradients in mortality. *Am J Public Health* 2008;98(4):699-704. doi: 10.2105/AJPH.2007.109637
2. Toch-Marquardt M, Menvielle G, Eikemo TA, et al. Occupational class inequalities in all-cause and cause-specific mortality among middle-aged men in 14 European populations during the early 2000s. *PLoS One* 2014;9(9):e108072. doi: 10.1371/journal.pone.0108072
3. Berenson GS. Childhood risk factors predict adult risk associated with subclinical cardiovascular disease. The Bogalusa Heart Study. *Am J Cardiol* 2002;90(10C):3L-7L.
4. Mahoney LT, Burns TL, Stanford W, et al. Coronary risk factors measured in childhood and young adult life are associated with coronary artery calcification in young adults: the Muscatine Study. *Journal of the American College of Cardiology* 1996;27(2):277-84. [published Online First: 1996/02/01]
5. Puolakka E, Pahkala K, Laitinen TT, et al. Childhood Socioeconomic Status and Arterial Stiffness in Adulthood: The Cardiovascular Risk in Young Finns Study. *Hypertension* 2017;70(4):729-35. doi: 10.1161/HYPERTENSIONAHA.117.09718
6. Expert Panel on Integrated Guidelines for Cardiovascular H, Risk Reduction in C, Adolescents, et al. Expert panel on integrated guidelines for cardiovascular health and risk reduction in children and adolescents: summary report. *Pediatrics* 2011;128 Suppl 5:S213-56. doi: 10.1542/peds.2009-2107C
7. Must A, Jacques PF, Dallal GE, et al. Long-term morbidity and mortality of overweight adolescents. A follow-up of the Harvard Growth Study of 1922 to 1935. *N Engl J Med* 1992;327(19):1350-5. doi: 10.1056/NEJM199211053271904
8. Henriksson P, Henriksson H, Labayen I, et al. Correlates of ideal cardiovascular health in European adolescents: The HELENA study. *Nutrition, metabolism, and cardiovascular diseases : NMCD* 2018;28(2):187-94. doi: 10.1016/j.numecd.2017.10.018
9. Leino M, Porkka KV, Raitakari OT, et al. Influence of parental occupation on coronary heart disease risk factors in children. The Cardiovascular Risk in Young Finns Study. *Int J Epidemiol* 1996;25(6):1189-95.
10. Seo YG, Choi MK, Kang JH, et al. Cardiovascular disease risk factor clustering in children and adolescents: a prospective cohort study. *Archives of disease in childhood* 2018;103(10):968-73. doi: 10.1136/archdischild-2017-313226 [published Online First: 2018/04/14]
11. Janssen I, Katzmarzyk PT, Boyce WF, et al. Comparison of overweight and obesity prevalence in school-aged youth from 34 countries and their relationships with physical activity and dietary patterns. *Obes Rev* 2005;6(2):123-32. doi: 10.1111/j.1467-789X.2005.00176.x
12. Pate RR, O'Neill JR, Liese AD, et al. Factors associated with development of excessive fatness in children and adolescents: a review of prospective studies. *Obes Rev* 2013;14(8):645-58. doi: 10.1111/obr.12035
13. Iqbal R, Anand S, Ounpuu S, et al. Dietary patterns and the risk of acute myocardial infarction in 52 countries: results of the INTERHEART study. *Circulation* 2008;118(19):1929-37. doi: 10.1161/circulationaha.107.738716 [published Online First: 2008/10/22]
14. OECD. Key indicators on the distribution of household disposable income and poverty, 2007, 2015 and 2016 or most recent year. [Available from: http://www.oecd.org/social/soc/IDD-Key-Indicators.xlsx accessed 4 november 2019.
15. Wettergren B, Blennow M, Hjern A, et al. Child Health Systems in Sweden. *The Journal of pediatrics* 2016;177s:S187-s202. doi: 10.1016/j.jpeds.2016.04.055 [published Online First: 2016/09/27]
16. Försäkringskassan. When the child is born [Available from: https://www.forsakringskassan.se/privatpers/foralder/nar_barnet_ar_fott/ accessed 4 november 2019.

For peer review only - http://bmjopen.bmj.com/site/about/guidelines.xhtml

17. Statistics Sweden. Population in the country, counties and municipalities on 31 March, 2019 and population change in January–March 2019 [Available from: https://www.scb.se/en/finding-statistics/statistics-by-subject-area/population/population-composition/population-statistics/pong/tables-and-graphs/quarterly-population-statistics--municipalities-counties-and-the-whole-country/quarter-1-2019/ accessed 4 november 2019.
18. Statistics Sweden. Income and tax statistics [Available from: https://www.scb.se/en/finding-statistics/statistics-by-subject-area/household-finances/income-and-income-distribution/income-and-tax-statistics/ accessed 4 november 2019.
19. National High Blood Pressure Education Program Working Group on High Blood Pressure in C, Adolescents. The fourth report on the diagnosis, evaluation, and treatment of high blood pressure in children and adolescents. *Pediatrics* 2004;114(2 Suppl 4th Report):555-76.
20. Gibbs BG, Forste R. Socioeconomic status, infant feeding practices and early childhood obesity. *Pediatr Obes* 2014;9(2):135-46. doi: 10.1111/j.2047-6310.2013.00155.x
21. Cole TJ, Bellizzi MC, Flegal KM, et al. Establishing a standard definition for child overweight and obesity worldwide: international survey. *BMJ (Clinical research ed)* 2000;320(7244):1240-3.
22. Cole TJ, Lobstein T. Extended international (IOTF) body mass index cut-offs for thinness, overweight and obesity. *Pediatr Obes* 2012;7(4):284-94. doi: 10.1111/j.2047-6310.2012.00064.x
23. National Cholesterol Education Program (NCEP): highlights of the report of the Expert Panel on Blood Cholesterol Levels in Children and Adolescents. *Pediatrics* 1992;89(3):495-501.
24. Statistics Sweden. Socioekonomisk indelning [SEI], MIS 1982:4 SEI. [Available from: https://www.scb.se/contentassets/22544e89c6f34ce7ac2e6fefbda407ef/mis1982_4_sei.pdf/ accessed 4 november 2019.
25. Faresjo T, Rahmqvist M. Educational level is a crucial factor for good perceived health in the local community. *Scand J Public Health* 2010;38(6):605-10. doi: 10.1177/1403494810374676
26. Wennerholm C, Grip B, Johansson A, et al. Cardiovascular disease occurrence in two close but different social environments. *Int J Health Geogr* 2011;10:5. doi: 10.1186/1476-072X-10-5
27. Karlen J, Faresjo T, Ludvigsson J. Could the social environment trigger the induction of diabetes related autoantibodies in young children? *Scand J Public Health* 2012;40(2):177-82. doi: 10.1177/1403494811435491 [published Online First: 2012/02/09]
28. Ferrie JE, Shipley MJ, Davey Smith G, et al. Change in health inequalities among British civil servants: the Whitehall II study. *Journal of epidemiology and community health* 2002;56(12):922-6.
29. Steptoe A, Kunz-Ebrecht S, Owen N, et al. Socioeconomic status and stress-related biological responses over the working day. *Psychosomatic medicine* 2003;65(3):461-70. [published Online First: 2003/05/24]
30. Ruiz M, Goldblatt P, Morrison J, et al. Impact of Low Maternal Education on Early Childhood Overweight and Obesity in Europe. *Paediatr Perinat Epidemiol* 2016;30(3):274-84. doi: 10.1111/ppe.12285
31. Tabbakh T, Freeland-Graves JH. The home environment: A mediator of nutrition knowledge and diet quality in adolescents. *Appetite* 2016;105:46-52. doi: 10.1016/j.appet.2016.05.002 [published Online First: 2016/05/14]
32. Gebremariam MK, Henjum S, Hurum E, et al. Mediators of the association between parental education and breakfast consumption among adolescents : the ESSENS study. *BMC pediatrics* 2017;17(1):61. doi: 10.1186/s12887-017-0811-2 [published Online First: 2017/02/24]
33. Gebremariam MK, Lien N, Torheim LE, et al. Perceived rules and accessibility: measurement and mediating role in the association between parental education and vegetable and soft drink intake. *Nutrition journal* 2016;15(1):76. doi: 10.1186/s12937-016-0196-3 [published Online First: 2016/08/18]

For peer review only

34. Angbratt M, Ekberg J, Walter L, et al. Prediction of obesity from infancy to adolescence. *Acta Paediatr* 2011;100(9):1249-52. doi: 10.1111/j.1651-2227.2011.02326.x
35. Howe LD, Tilling K, Galobardes B, et al. Loss to follow-up in cohort studies: bias in estimates of socioeconomic inequalities. *Epidemiology* 2013;24(1):1-9. doi: 10.1097/EDE.0b013e31827623b1
36. Kagura J, Adair LS, Pisa PT, et al. Association of socioeconomic status change between infancy and adolescence, and blood pressure, in South African young adults: Birth to Twenty Cohort. *BMJ Open* 2016;6(3):e008805. doi: 10.1136/bmjopen-2015-008805
37. Krmar RT, Holtback U, Bergh A, et al. Oscillometric casual blood pressure normative standards for Swedish children using ABPM to exclude casual hypertension. *Am J Hypertens* 2015;28(4):459-68. doi: 10.1093/ajh/hpu182
38. Brettschneider AK, Ellert U, Schaffrath Rosario A. Comparison of BMI derived from parent-reported height and weight with measured values: results from the German KiGGS study. *International journal of environmental research and public health* 2012;9(2):632-47. doi: 10.3390/ijerph9020632 [published Online First: 2012/04/04]
39. Commission on Social Determinants of Health. CSDH Final Report: Closing the Gap in a Generation: Health Equity Through Action on the Social Determinant of Health. Geneva, World Health Organization, 2008.
40. OECD. In It Together: Why Less Inequality Benefits All. Paris, OECD Publishing, 2015. https://doi.org/10.1787/9789264235120-en/.
41. Juhola J, Magnussen CG, Viikari JS, et al. Tracking of serum lipid levels, blood pressure, and body mass index from childhood to adulthood: the Cardiovascular Risk in Young Finns Study. *The Journal of pediatrics* 2011;159(4):584-90. doi: 10.1016/j.jpeds.2011.03.021 [published Online First: 2011/04/26]
42. Raitakari OT, Juonala M, Kahonen M, et al. Cardiovascular risk factors in childhood and carotid artery intima-media thickness in adulthood: the Cardiovascular Risk in Young Finns Study. *Jama* 2003;290(17):2277-83. doi: 10.1001/jama.290.17.2277 [published Online First: 2003/11/06]
43. Leon DA, Vagero D, Olausson PO. Social class differences in infant mortality in Sweden: comparison with England and Wales. *BMJ (Clinical research ed)* 1992;305(6855):687-91.
44. Kunst AE, Mackenbach JP. The size of mortality differences associated with educational level in nine industrialized countries. *Am J Public Health* 1994;84(6):932-7.
45. Pickett KE, Wilkinson RG. Child wellbeing and income inequality in rich societies: ecological cross sectional study. *BMJ (Clinical research ed)* 2007;335(7629):1080. doi: 10.1136/bmj.39377.580162.55 [published Online First: 2007/11/21]
46. Lundberg O. The next step towards more equity in health in Sweden: how can we close the gap in a generation? *Scand J Public Health* 2018;46(22_suppl):19-27. doi: 10.1177/1403494818765702 [published Online First: 2018/06/05].

For peer review only - http://bmjopen.bmj.com/site/about/guidelines.xhtml

Table 1. Characteristics of adolescents by hypertension and overweight status.

	Normal blood pressure		Elevated blood pressure		Not overweight		Overweight	
	N	%	N	%	N	%	N	%
Total	240	83.0	49	17.0	244	84.1	46	15.9
Sex								
Boy	105	43.8	27	55.1	109	44.7	24	52.2
Girl	135	56.2	22	44.9	135	55.3	22	47.8
Ethnicity								
Swedish	213	89.9	44	91.7	218	90.5	40	88.9
Foreign ethnicity	24	10.1	4	8.3	23	9.5	5	11.1
Physical activity								
– times per week								
>2	138	67.6	31	83.8	149	72.0	21	60.0
0-2	66	32.4	6	16.2	58	28.0	14	40.0
CRD								
0-1 item	88	44.4	14	40.0	94	46.5	8	25.0
2-5 items	110	55.6	21	60.0	108	53.5	24	75.0
BMI group of adolescent								
Not OW	213	88.8	31	63.3				
OW/OB	27	11.2	18	36.7				
Income								
High	94	39.5	16	33.3	99	40.9	12	26.7
Middle	111	46.6	26	54.2	112	46.3	25	55.6
Low	33	13.9	6	12.5	31	12.8	8	17.8
Maternal education								
Tertiary	138	58.2	20	41.7	144	59.8	14	31.1
<Tertiary	99	41.8	28	58.3	97	40.2	31	68.9
SEI								
Class I	59	24.6	5	10.2	60	24.6	4	8.7

For peer review only - http://bmjopen.bmj.com/site/about/guidelines.xhtml

For peer review only

Class II-IV	131	54.6	25	51.0	130	53.3	27	58.7
Class V	50	20.8	19	38.8	54	22.1	15	32.6
City of residence								
White-collar	132	55	23	46.9	137	56.1	18	39.1
Blue-collar	108	45	26	53.1	107	43.9	28	60.9
	M	SD	M	SD	M	SD	M	SD
Age at follow-up (years)	16.02	0.90	16.12	1.04	16.02	0.92	16.18	0.94
Height (m)	1.72	0.09	1.73	0.10	1.73	0.09	1.71	0.09

BMI, Body Mass Index; *CRD*, Cardiovascular risk diet index; *OW/OB*, Overweight or obese; *SEI*, Swedish socioeconomic classification index.

For peer review only

Table 2. Univariate analysis with odds ratios for cardiovascular risk factors and areas under the ROC-curve for SES variables (n = 298).

	Elevated blood pressure		Low HDL		High LDL		Overweight	
	OR	CI	OR	CI	OR	CI	OR	CI
Age at follow-up (Z-years)	1.12	0.83,1.51	1.12	0.79,1.59	0.99	0.63,1.53	1.19	0.87,1.63
Height (Z-m)	1.24	0.91,1.64	1.41	0.85,2.33	1.26	0.85,1.88	0.82	0.59,1.14
Gender								
Girl	1.0		1.0		1.0		1.0	
Boy	1.61	0.86,3.04	2.78	1.09,7.09	1.31	0.57,3.00	1.38	0.73,2.60
Ethnicity								
Swedish	1.0		1.0		1.0		1.0	
Foreign	0.83	0.27,2.58	1.49	0.53,4.19	0.29	0.04,2.25	1.13	0.41,3.16
Physical activity								
– times/week								
>2	1.0		1.0		1.0		1.0	
0-2	0.39	0.15,1.05	0.93	0.41,2.26	0.68	0.28,1.67	1.60	0.72,3.55
CRD								
0-1 item	1.0		1.0		1.0		1.0	
2-5 items	1.12	0.49,2.61	2.41	0.95,6.15	1.41	0.63,3.16	2.80	1.06,7.41
BMI group of adolescent								
Not OW	1.0		1.0		1.0			
OW/OB	4.42	2.19,8.95	3.44	1.47,8.07	3.20	1.28,7.98		
Income								
(ROC)	0.537		0.627		0.545		0.580	
High	1.0		1.0		1.0		1.0	
Middle	1.35	0.68,2.67	0.88	0.36,2.16	1.08	0.42,2.77	1.90	0.91,3.97

For peer review only

For peer review only - http://bmjopen.bmj.com/site/about/guidelines.xhtml

Low	1.11	0.41,3.05	3.94	1.39,11.14	1.73	0.46,6.54	2.21	0.82,5.95
Maternal education (ROC)	0.578		0.610		0.564		0.641	
Tertiary	1.0		1.0		1.0		1.0	
<Tertiary	1.87	0.99,3.53	2.39	1.10,5.21	1.67	0.77,3.59	3.22	1.61,6.44
SEI								
(ROC)	0.623		0.540		0.573		0.602	
Class I	1.0		1.0		1.0		1.0	
Class II-IV	2.20	0.80,6.05	1.33	0.48,3.66	1.91	0.60,6.09	3.21	1.08,9.57
Class V	4.37	1.49,12.81	1.45	0.48,4.48	1.41	0.24,8.28	4.28	1.34,13.73
City of residence (ROC)	0.541		0.652		0.511		0.585	
White-collar	1.0		1.0		1.0		1.0	
Blue-collar	1.39	0.76,2.56	1.78	0.74,4.31	1.10	0.51,2.41	2.00	1.06,3.78

BMI, Body Mass Index; *CRD*, Cardiovascular risk diet index; *CI*, 95% confidence interval; *OR*, odds ratio; *OW/OB*, Overweight or obese; *ROC*, area under the receiver operating characteristic curve; *SEI*, Swedish socioeconomic classification index.

For peer review only

For peer review only - http://bmjopen.bmj.com/site/about/guidelines.xhtml

Table 3. Multivariate analysis with odds ratios for cardiovascular risk factors. Adjusted for SES and other explanatory variables (n = 298).

		Elevated blood pressure					
		Model 1		Model 2		Model 3	
		OR	CI	OR	CI	OR	CI
Income	High	1.00		1.00		1.00	
	Middle	0.85	0.39,1.85	0.85	0.38,1.89	0.75	0.32,1.75
	Low	0.60	0.20,1.81	0.69	0.22,2.12	0.60	0.17,1.96
Maternal education	Tertiary	1.00		1.00		1.00	
	<Tertiary	1.29	0.60,2.77	1.15	0.52,2.54	0.88	0.37,2.08
SEI	Class I	1.00		1.00		1.00	
	Class II-IV	2.21	0.77,6.34	2.25	0.78,6.52	2.09	0.70,6.27
	Class V	4.18	1.22,14.41	4.43	1,26,15.58	4.95	1.30,18.89
City of residence	White-collar	1.00		1.00		1.00	
	Blue-collar	1.12	0.58,2.14	1.18	0.61,2.30	1.05	0.52,2.13

		Low HDL (<0.40 mg/dL).					
		Model 1		Model 2		Model 3	
		OR	CI	OR	CI	OR	CI
Income	High	1.00		1.00		1.00	
	Middle	0.98	0.31,3.08	0.72	0.26,1.98	0.73	0.26,2.20
	Low	3.16	0.97,10.35	3.28	0.92,11.67	3.41	0.89,13.06
Maternal education	Tertiary	1.00		1.00		1.00	
	<Tertiary	2.56	0.99,6.62	2.29	0.86,6.09	2.00	0.73,5.50
SEI	Class I	1.00		1.00		1.00	
	Class II-IV	0.88	0.32,2.48	0.82	0.29,2.36	0.76	0.26,2.10
	Class V	0.53	0.14,2.07	0.41	0.10,1.73	0.41	0.09,1.75
City of residence	White-collar	1.00		1.00		1.00	
	Blue-collar	1.41	0.55,3.60	1.35	0.53,3.48	1.22	0.46,3.27

High LDL (>110 mg/dL)

For peer review only

		Model 1		Model 2		Model 3	
		OR	CI	OR	CI	OR	CI
Income	High	1.00		1.00		1.00	
	Middle	0.91	0.32,2.56	0.88	0.31,2.51	0.84	0.29,2.49
	Low	1.48	0.35,6.21	1.47	0.34,6.29	1.41	0.31,6.39
Maternal education	Tertiary	1.00		1.00		1.00	
	<Tertiary	1.86	0.70,4.93	1.81	0.68,4.79	1.58	0.58,4.32
SEI	Class I	1.00		1.00		1.00	
	Class II-IV	1.69	0.49,5.77	1.68	0.49,5.79	1.52	0.43,5.40
	Class V	0.92	0.10,8.56	0.97	0.10,9.06	0.90	0.09,9.54
City of residence	White-collar	1.00		1.00		1.00	
	Blue-collar	0.89	0.37,2.15	0.90	0.37,2.18	0.83	0.33,2.09

		Overweight status			
		Model 1		Model 2	
		OR	CI	OR	CI
Income	High	1.00		1.00	
	Middle	1.16	0.52,2.60	1.12	0.50,2.54
	Low	1.21	0.42,3.54	1.20	0.40,3.56
Maternal education	Tertiary	1.00		1.00	
	<Tertiary	2.63	1.20,5.78	2.39	1.08,5.30
SEI	Class I	1.00		1.00	
	Class II-IV	2.34	0.75,7.28	2.09	0.66,6.60
	Class V	1.90	0.52,6.98	1.63	0.43,6.25
City of residence	White-collar	1.00		1.00	
	Blue-collar	1.39	0.70,2.73	1.39	0.70,2.76

BMI, Body Mass Index; *CI*, 95% confidence interval; *OR*, odds ratio; *SEI*, Swedish socioeconomic classification index.

Model 1 includes all SES variables. Model 2 includes all SES and other variables excluding overweight status that reached the selection criteria in univariate analysis ($p \leq 0.05$ or OR <0.5 or >2.0 for categorical and <0.75 or >1.5 for continuous variables): physical activity for elevated blood pressure; gender and cardiovascular risk diet for low HDL; ethnicity for LDL; cardiovascular risk diet for overweight. Model 3 includes all SES, other explanatory variables and overweight status.

For peer review only - http://bmjopen.bmj.com/site/about/guidelines.xhtml

Table 4. Association between maternal education level at birth and child overweight or obesity at four ages.

Time point:	2 years	5 years	8 years	12 years
Cohort approach				
Age: mean (SD)	2.72 (0.28)	5.32 (0.30)	7.86 (0.34)	12.61 (0.87)
Sample size available	1070	1070	1070	1070
Overweight: n (%)	149 (13.9%)	146 (13.6%)	157 (14.7%)	168 (15.7%)
Obese: n (%)	19 (1.8%)	27 (2.5%)	21 (2.0%)	21 (2.0%)
Tertiary v < Tertiary education: overweight*	0.95 (0.67, 1.34)	1.42 (1.00, 2.03)	1.36 (0.96, 1.92)	1.81 (1.28, 2.55)
Tertiary v < Tertiary education: obesity*	0.41 (0.15, 1.08)	3.21 (1.29, 8.02)	1.46 (0.60, 3.56)	3.89 (1.30, 11.63)
Available sample approach				
Age: mean (SD)	2.73 (0.30)	5.34 (0.31)	7.86 (0.38)	12.40 (0.91)
Sample size available	7925	6602	2998	3337
Overweight: n (%)	1187 (15.0%)	1096 (16.6%)	446 (14.9%)	527 (15.8%)
Obese: n (%)	191 (2.4%)	273 (4.1%)	71 (2.4%)	75 (2.2%)
Tertiary v < Tertiary education: overweight*	1.18 (1.03, 1.34)	1.30 (1.13, 1.50)	1.16 (0.94, 1.42)	1.70 (1.39, 2.06)
Tertiary v < Tertiary education: obesity*	1.56 (1.12, 2.17)	1.52 (1.15, 1.99)	2.28 (1.31, 3.95)	2.16 (1.29, 3.61)

*Odds ratio (95% confidence interval)

Note: The cohort approach utilises only participants with all necessary data recorded at all four ages whereas the available sample approach uses any participant with all necessary data recorded on at least one age.

For peer review only - http://bmjopen.bmj.com/site/about/guidelines.xhtml

Supplementary table 1: Comparison of original ABIS cohort, invited subsample, participants and non-participants in the cardiovascular risk study.

	Original cohort N=16349		Invited to subsample N=578		Participants N=298		Non- participants N=240		p[1]	p[2]	p-[3]
	Mean/N	SD/%	Mean/N	SD/%	Mean/N	SD/%	Mean/N	SD/%			
Birthweight (g)	3578.6	560.8	3552.6	553.6	3554.0	574.7	3551.1	530.8	0.27	0.46	0.95
Gender									0.11	0.05	0.23
Boys	8473	51.9 %	279	48.5	136	46.1	143	46.1 %			
Girls	7866	48.1 %	296	51.5	159	53.9	137	48.9 %			
Ethnicity									0.09	0.50	0.01
Swedish	14235	89.3 %	492	87.1 %	266	90.5 %	226	83.4 %			
Other	1711	10.7 %	73	12.9 %	28	9.5 %	45	16.6 %			
Father ow/ob[a]									0.19	0.54	0.51
Not ow/ob	5384	54.0 %	254	56.4 %	146	57.3 %	108	57.3 %			
Overweight	4003	40.1 %	164	36.4 %	94	36.9 %	70	35.9 %			
Obese	586	5.9 %	32	7.1 %	15	5.9 %	17	8.7 %			
Mother ow/ob[a]									0.95	0.22	0.01
Not ow/ob	7310	70.0 %	328	69.6 %	191	72.9 %	137	65.6 %			

For peer review only

	Overweight	2372	22.7 %	107	22.7 %	59	22.5 %	48	23.0 %			
	Obese	759	7.3 %	36	7.6 %	12	4.6 %	24	11.5 %			
Overweight at 2 yrs										0.46	0.75	0.71
	Not ow/ob	6890	85.0 %	307	83.7 %	187	84.2 %	120	82.8 %			
	Ow/ob	1217	15.0 %	60	16.3 %	35	15.8 %	25	17.2 %			
Overweight at 5 yrs										0.35	<0.01	<0.01
	Not ow/ob	5624	83.3 %	287	85.2 %	187	90.3 %	100	76.9 %			
	Ow/ob	1125	16.7 %	50	14.8 %	20	9.7 %	30	23.1 %			
Overweight at 8 yrs										0.76	0.51	0.10
	Not ow/ob	2566	85.1 %	2566	85.1 %	139	86.9 %	50	78.1 %			
	Ow/ob	450	14.9 %	450	14.9 %	21	13.1 %	14	21.9 %			
Overweight at 12 yrs										0.63	0.20	0.08
	Not ow/ob	2836	84.2 %	197	85.3 %	155	87.6 %	42	77.8 %			
	Ow/ob	534	15.8 %	34	14.7 %	22	12.4 %	12	22,2 %			
Maternal education										< 0.01	< 0.01	<0.01
	High	5062	31.8 %	233	41.3 %	160	54.4 %	73	27.0 %			
	Low	10882	68.2 %	331	58.7 %	134	45.6 %	197	73.0 %	197	73.0 %	
Income										< 0.01	< 0.01	< 0.01
	High	3239	20.0 %	153	26.7 %	114	38.6 %	39	14.0 %			
	Middle	9718	60.0 %	301	52.5 %	141	47.8 %	160	57.6 %			
	Low	3239	20.0 %	119	20.8 %	40	13.6 %	79	28.4 %			

For peer review only

p-value[1]= Invited to follow-up vs original cohort, T-test or Chi2 as appropriate
p-value[2]= Participants in follow-up vs original cohort, T-test or Chi2 as appropriate
p-value[3]=Participants vs non-participants in follow-up, T-test or Chi2 as appropriate
[a] Parental height and weight self-reported at child age 1 year
T-test or Chi2 as appropriate

For peer review only

Reporting checklist for cohort study.

Based on the STROBE cohort guidelines.

Instructions to authors

Complete this checklist by entering the page numbers from your manuscript where readers will find each of the items listed below.

Your article may not currently address all the items on the checklist. Please modify your text to include the missing information. If you are certain that an item does not apply, please write "n/a" and provide a short explanation.

Upload your completed checklist as an extra file when you submit to a journal.

In your methods section, say that you used the STROBE cohort reporting guidelines, and cite them as:

von Elm E, Altman DG, Egger M, Pocock SJ, Gotzsche PC, Vandenbroucke JP. The Strengthening the Reporting of Observational Studies in Epidemiology (STROBE) Statement: guidelines for reporting observational studies.

		Reporting Item	Page Number
Title	#1a	Indicate the study's design with a commonly used term in the title or the abstract	1
Abstract	#1b	Provide in the abstract an informative and balanced summary of what was done and what was found	2
Background / rationale	#2	Explain the scientific background and rationale for the investigation being reported	4
Objectives	#3	State specific objectives, including any prespecified hypotheses	5
Study design	#4	Present key elements of study design early in the paper	6
Setting	#5	Describe the setting, locations, and relevant dates, including periods of recruitment, exposure, follow-up, and data collection	6-7

For peer review only

Eligibility criteria	#6a	Give the eligibility criteria, and the sources and methods of selection of participants. Describe methods of follow-up.	6-7
	#6b	For matched studies, give matching criteria and number of exposed and unexposed	n/a
Variables	#7	Clearly define all outcomes, exposures, predictors, potential confounders, and effect modifiers. Give diagnostic criteria, if applicable	7-10
Data sources / measurement	#8	For each variable of interest give sources of data and details of methods of assessment (measurement). Describe comparability of assessment methods if there is more than one group. Give information separately for for exposed and unexposed groups if applicable.	7-10
Bias	#9	Describe any efforts to address potential sources of bias	10
Study size	#10	Explain how the study size was arrived at	5
Quantitative variables	#11	Explain how quantitative variables were handled in the analyses. If applicable, describe which groupings were chosen, and why	7-10
Statistical methods	#12a	Describe all statistical methods, including those used to control for confounding	10
	#12b	Describe any methods used to examine subgroups and interactions	N/A
	#12c	Explain how missing data were addressed	10
	#12d	If applicable, explain how loss to follow-up was addressed	6-7
	#12e	Describe any sensitivity analyses	N/A
Participants	#13a	Report numbers of individuals at each stage of study—eg numbers potentially eligible, examined for eligibility, confirmed eligible, included in the study, completing follow-up, and analysed. Give information separately for for exposed and unexposed groups if applicable.	12-13
	#13b	Give reasons for non-participation at each stage	7
	#13c	Consider use of a flow diagram	N/A

For peer review only

Descriptive data	#14a	Give characteristics of study participants (eg demographic, clinical, social) and information on exposures and potential confounders. Give information separately for exposed and unexposed groups if applicable.	Table 1
	#14b	Indicate number of participants with missing data for each variable of interest	Table 1
	#14c	Summarise follow-up time (eg, average and total amount)	6
Outcome data	#15	Report numbers of outcome events or summary measures over time. Give information separately for exposed and unexposed groups if applicable.	12-13
Main results	#16a	Give unadjusted estimates and, if applicable, confounder-adjusted estimates and their precision (eg, 95% confidence interval). Make clear which confounders were adjusted for and why they were included	10-13
	#16b	Report category boundaries when continuous variables were categorized	8-10
	#16c	If relevant, consider translating estimates of relative risk into absolute risk for a meaningful time period	N/A
Other analyses	#17	Report other analyses done—e.g., analyses of subgroups and interactions, and sensitivity analyses	N/A
Key results	#18	Summarise key results with reference to study objectives	14
Limitations	#19	Discuss limitations of the study, taking into account sources of potential bias or imprecision. Discuss both direction and magnitude of any potential bias.	15-16
Interpretation	#20	Give a cautious overall interpretation considering objectives, limitations, multiplicity of analyses, results from similar studies, and other relevant evidence.	17-18
Generalisability	#21	Discuss the generalisability (external validity) of the study results	15-16
Funding	#22	Give the source of funding and the role of the funders for the present study and, if applicable, for the original study on which the present article is based	18

For peer review only

The STROBE checklist is distributed under the terms of the Creative Commons Attribution License CC-BY. This checklist was completed on 22. March 2019 using https://www.goodreports.org/, a tool made by the EQUATOR Network in collaboration with Penelope.ai

For peer review only
